# Evaluation of Digital Mental Health Technologies in the United States: Systematic Literature Review and Framework Synthesis

**DOI:** 10.2196/57401

**Published:** 2024-08-30

**Authors:** Julianna Catania, Steph Beaver, Rakshitha S Kamath, Emma Worthington, Minyi Lu, Hema Gandhi, Heidi C Waters, Daniel C Malone

**Affiliations:** 1 Costello Medical Boston, MA United States; 2 Costello Medical Cambridge United Kingdom; 3 Otsuka Pharmaceutical Development & Commercialization Inc Princeton, NJ United States; 4 Department of Pharmacotherapy Skaggs College of Pharmacy University of Utah Salt Lake City, UT United States

**Keywords:** mental health, mobile health, mHealth, digital health, digital therapeutics, systematic review, framework synthesis, mixed methods

## Abstract

**Background:**

Digital mental health technologies (DMHTs) have the potential to enhance mental health care delivery. However, there is little information on how DMHTs are evaluated and what factors influence their use.

**Objective:**

A systematic literature review was conducted to understand how DMHTs are valued in the United States from user, payer, and employer perspectives.

**Methods:**

Articles published after 2017 were identified from MEDLINE, Embase, PsycINFO, Cochrane Library, the Health Technology Assessment Database, and digital and mental health congresses. Each article was evaluated by 2 independent reviewers to identify US studies reporting on factors considered in the evaluation of DMHTs targeting mental health, Alzheimer disease, epilepsy, autism spectrum disorder, or attention-deficit/hyperactivity disorder. Study quality was assessed using the Critical Appraisal Skills Program Qualitative and Cohort Studies Checklists. Studies were coded and indexed using the American Psychiatric Association’s Mental Health App Evaluation Framework to extract and synthesize relevant information, and novel themes were added iteratively as identified.

**Results:**

Of the 4353 articles screened, data from 26 unique studies from patient, caregiver, and health care provider perspectives were included. Engagement style was the most reported theme (23/26, 88%), with users valuing DMHT usability, particularly alignment with therapeutic goals through features including anxiety management tools. Key barriers to DMHT use included limited internet access, poor technical literacy, and privacy concerns. Novel findings included the discreetness of DMHTs to avoid stigma.

**Conclusions:**

Usability, cost, accessibility, technical considerations, and alignment with therapeutic goals are important to users, although DMHT valuation varies across individuals. DMHT apps should be developed and selected with specific user needs in mind.

## Introduction

### Background

Digital health comprises a broad range of technologies, including mobile health, health information technology, wearable devices, and personalized medicine, which serve as tools to enhance health care delivery. Recently, several digital mental health (MH) therapeutics, a category of digital MH technologies (DMHTs), have received US Food and Drug Administration (FDA) approval to prevent, manage, or treat a medical disorder or disease based on evidence from superiority trials and compliance with technical guidelines [1,2]. However, most DMHTs, particularly apps, fall outside FDA jurisdiction because they are not intended to diagnose, treat, or prevent disease and because they are “low risk” in that they would not cause harm in the event of malfunction [3]. Due to this lack of regulatory framework, few DMHTs are supported by published efficacy studies. One study found that only 16% of MH apps recommended by college counseling centers were supported by efficacy studies published in peer-reviewed journals [4].

Nonetheless, many health care providers (HCPs) use MH apps in clinical practice. Up to 83% of behavioral health providers in a small study covering the Greater Boston area reported using apps as part of their clinical care, particularly mindfulness apps for patient anxiety management [5]. As many DMHTs are currently widely used in clinical practice without undergoing any formal assessment for quality or relevance, understanding how DMHTs should be assessed based on factors impacting their value from the perspective of key stakeholders, such as patients, caregivers, providers, payers, and employers, could improve the selection of DMHTs for use by patients, thereby increasing care quality and outcomes for those seeking MH support.

### Objective

To address identified gaps, a systematic literature review (SLR) was conducted using a published framework to synthesize emerging themes from mixed methods evidence in order to understand how digital health solutions, encompassing both digital therapeutics and direct-to-consumer digital health technologies, are valued, with a focus on MH disorders, Alzheimer disease, epilepsy, autism spectrum disorder (ASD), and attention-deficit/hyperactivity disorder (ADHD) in the United States.

## Methods

### Overview

The SLR was performed in accordance with a prespecified protocol and reported in line with the Enhancing Transparency in Reporting the Synthesis of Qualitative Research and PRISMA (Preferred Reporting Items for Systematic Reviews and Meta-Analyses) guidelines [6,7]. The protocol was not registered.

### Search Strategy

Electronic databases, encompassing MEDLINE (including MEDLINE In-Process, MEDLINE Daily, and MEDLINE Epub Ahead of Print); Embase; the Cochrane Library (including Cochrane Database of Systematic Reviews and Cochrane Central Register of Controlled Trials); PsycINFO; and the Health Technology Assessment Database, were selected in alignment with this SLR’s target indications and were searched on June 17, 2022. The search terms included combinations of free-text and Medical Subject Heading or Emtree terms related to indications of interest, DMHTs, and relevant outcomes or assessment types (eg, technology assessments and cost; Tables S1-S5 in Multimedia Appendix 1). Searches were limited to studies performed in the United States and to those published from 2017 onward.

Manual hand searches of gray literature, namely, the bibliographies of relevant SLRs identified from the electronic database searches and key conference proceedings (2019-2022), were performed to identify additional studies of relevance (Table S6 in Multimedia Appendix 1). The FDA website was also searched to identify factors involved in the FDA’s appraisal of relevant MH apps, which could supplement the factors identified in this SLR (Table S7 in Multimedia Appendix 1).

### Study Selection

Studies were included in the SLR if they met prespecified criteria defined using the SPIDER (Sample, Phenomenon of Interest, Design, Evaluation, Research type) framework, which is appropriate for mixed methods research questions. Eligible studies were published in the English language, were set in the United States, and reported quantitative or qualitative outcomes relating to the factors considered in the evaluation of DMHTs. Only studies published in 2017 or later were included because of the rapidly evolving research area. Eligible studies reported on MH, Alzheimer disease, epilepsy, ASD, or ADHD from user, payer, or employer perspectives (Table S8 in Multimedia Appendix 1). While the primary focus of the SLR was MH, neurological conditions were also of interest because their pathologies, symptoms, and treatment strategies can overlap with those of mental illnesses. Alzheimer disease, epilepsy, ASD, and ADHD were selected because they are highly researched and represent diverse types of neurological conditions.

The titles and abstracts of records were assessed for inclusion against these eligibility criteria by 2 independent reviewers, and discrepancies were resolved by consensus, with arbitration by a third reviewer if necessary. Full texts of potentially relevant articles were acquired and screened using the same methodology.

### Study Prioritization

Due to the large volume of the evidence identified, additional eligibility criteria were applied to prioritize primary research on theoretical DMHT valuation factors. In line with the thematic framework synthesis objective, *theoretical valuation factors* were defined as user or DMHT attributes that impact interaction with or perception of DMHTs; therefore, studies that reported only efficacy outcomes, such as mental illness symptom improvement, were deprioritized for full-text review. Secondary research was also deprioritized for full-text review. Studies that reviewed a select app against a framework and studies that reported only the outcomes specific to a select app were deprioritized for data extraction. For example, a study reporting the usability of a *specific* app’s features would have been deprioritized, while a study reporting what *types* of features increase MH app usability *in general* would not.

### Data Extraction

All relevant data were extracted into a prespecified Microsoft Excel grid, and a quality assessment was performed for each study. Studies that reported only qualitative data were assessed with the Critical Appraisal Skills Program Qualitative Studies Checklist. Studies that reported only quantitative data were evaluated with the Critical Appraisal Skills Program Cohort Study Checklist, and studies reporting both qualitative and quantitative data were evaluated with both checklists [8]. Data extractions and quality assessments were performed by a single individual for each study, with the information verified by a second independent individual. Discrepancies were resolved by consensus, with arbitration by a third individual if necessary.

### Framework Synthesis

A framework synthesis approach was undertaken to synthesize qualitative and quantitative data identified from the SLR. In line with the “best fit” framework synthesis approach, data were indexed deductively against an existing framework where possible, and novel themes were added inductively as needed [9,10]. The American Psychiatric Association (APA) Mental Health App Evaluation framework was considered the most appropriate framework to address the research objectives of this SLR because its key valuation themes were developed using psychiatrist and patient input, are broadly shared by other evaluation frameworks, are widely acknowledged in the literature, and have been described as durable and adaptable [11-13].

The APA model follows a hierarchical and chronological order whereby the evaluator moves through the framework using prompting questions (eg, “Does the app work offline?”). For this SLR, these questions were either thematically grouped into subthemes or left as prompting questions, as appropriate. The framework was therefore ultimately adapted into 3 levels: themes, subthemes, and more granular valuation criteria. It should be emphasized that this SLR did not aim to formally develop an updated framework to be used in practice by HCPs and their patients but rather was used to form a theoretical basis for understanding DMHT valuation factors, for which novel themes were expected to emerge.

A data-based convergent approach was used to synthesize quantitative and qualitative data [14]. Data were initially indexed deductively against the prespecified themes within the data collection instrument and then further synthesized within Docear [15], a mind-map software used to organize and connect data and concepts. Indexing was performed by 1 reviewer and checked by a second independent reviewer. New themes and subthemes that emerged from the literature through inductive coding were added post hoc to the thematic framework, with all extracted data then considered against both the prespecified and novel themes. The evidence identified for each theme was synthesized narratively, taking into consideration the context and design of each study.

## Results

### Included Studies

A total of 4974 records were retrieved from the electronic databases. Of the 3374 (67.83%) unique records identified following deduplication across the databases, 2891 (85.68%) were excluded based on the eligibility criteria, and an additional 456 (13.52%) were deprioritized because they were not directly related to the topic of interest for this SLR. Excluded and deprioritized full texts are listed in Tables S9 and S10 in Multimedia Appendix 1, respectively. Therefore, 27 (0.54%) articles were included from the electronic database searches. In addition, 1 article reporting on the same study as an already-included conference abstract was identified during supporting targeted searches and included as a supplementary record, resulting in a total of 28 articles reporting on 26 unique studies (Figure S1 in Multimedia Appendix 1). No relevant FDA appraisals were identified in the supplementary search.

Of the 26 included studies, 8 (31%) were quantitative, 12 (46%) were qualitative, and 6 (23%) used a mixed methods approach. While 5 (19%) studies assessed prospective cohorts, 22 (85%) used a cross-sectional approach, including 1 (4%) study that contained both a prospective cohort and a cross-sectional cohort (Table 1). All studies (26/26, 100%) investigated a user perspective, with none specifically investigating payer or employer perspectives. Only 1 (4%) study, which examined ingestible sensor pills and smart pill dispensers to track adherence, investigated a DMHT that was not an app [16].

**Table 1:** Summary of included study characteristics and outcomes.

Study (author, year)	Design^a^	Perspective and population^a^	Objectives	Data collection methods^a^
Afra et al [17], 2018	Cross-sectional, quantitative	Patients with epilepsy who were regular smartphone users recruited from the University of Utah Adult Comprehensive Epilepsy Clinic (N=40)	To develop a drug-device combination product using an app in combination with antiseizure medications as an epilepsy treatment	Custom survey
Beard et al [18], 2019	Cross-sectional, quantitative	Patients treated at a partial hospitalization program located in a nonprofit, insurance-based psychiatric hospital; diagnoses included MDD^b^, BD^c^, anxiety, OCD^d^, stress-related disorders, and psychotic disorders (N=322)	To characterize general smartphone app and social media use in an acute transdiagnostic psychiatric sample with high smartphone ownership, characterize current engagement and interest in the use of smartphone apps to support MH^e^, and test demographic and clinical predictors of smartphone use	Custom survey
Borghouts et al [19], 2022	Cross-sectional, mixed methods	General users^f^: members of the Center on Deafness Inland Empire, comprised people with lived experience as members of the deaf or hard-of-hearing community (N=10)	To investigate the MH needs of the deaf or hard-of-hearing community and how MH apps might support these needs	Custom survey; focus group
Boster and McCarthy [20], 2018	Cross-sectional, qualitative	Speech-language pathologists experienced in augmentative and alternative communication using a device in children with ASD^g^ recruited through social media and professional listserves (N=8) Parents (caregivers) of children with ASD recruited through national organizations (N=5)	To gain insight from speech-language pathologists and parents of children with ASD regarding appealing features of augmentative and alternative communication apps	Focus groups; poll questions
Buck et al [21], 2021a	Cross-sectional, quantitative	Caregivers of young adult family members who experienced early psychosis (onset before age 35) recruited through HCP^h^ referrals or ads (N=43)	To assess caregivers’ interest in an array of specific potential mHealth^i^ functions to guide the development of mHealth for caregivers of young adults with early psychosis	Custom survey
Buck et al [22], 2021b	Cross-sectional, quantitative	Users: young adults (aged 18-30 years) with a diagnosis of a psychotic disorder or self-reported history of psychotic symptoms recruited through HCP referrals or ads (N=77)	To understand the needs, interests, and preferences of young adults with early psychosis regarding mHealth by surveying interest in mHealth features and delivery modalities and by collecting information about their digital and web-based behaviors	Custom survey
Carpenter-Song et al [23], 2018	Prospective cohort, qualitative	Patients at a community MH center (N=15)	To examine current practices and orientations toward technology among consumers in 3 mental health settings in the United States	Semistructured interviews
Casarez et al [24], 2019	Cross-sectional, qualitative	Caregivers: spouses or partners of patients with BD recruited from a local outpatient psychiatry clinic or psychiatric hospital (N=13)	To explore how the well-being of spouses and partners of patients with BD can be improved through mHealth technology	Focus groups; minimally structured, open-ended individual interviews
Connolly et al [25], 2018	Cross-sectional, qualitative	Patients: US military veterans (aged 18-70 years) who screened positive for PTSD^j^, alcohol use disorder, or MDD during the previous year at 9 community-based VA^k^ outpatient clinics (N=66)	To examine veterans’ attitudes toward smartphone apps and to assess whether openness toward this technology varies by age or rurality	Semistructured interviews informed by the State of the Art Access Model
Cummings et al [26], 2019	Cross-sectional, qualitative	Parents and grandparents with children or grandchildren (caregivers) enrolled in a public health insurance program who received ≥2 months of ADHD^l^ treatment at 4 safety-net clinics (N=37) Administrators at the same clinics (N=41)	To examine stakeholder perspectives regarding whether mHealth tools can improve MH treatment for low-income youth with ADHD in safety-net settings and what functions would improve treatment	Focus groups (caregivers) and interviews (HCPs and staff), both semistructured and including open-ended questions and targeted probes
Dinkel et al [27], 2021	Cross-sectional, qualitative	Adult patients (aged ≥19 years) with a current or prior diagnosis of depression recruited during medical visits from 2 integrated primary care clinics (N=17) HCPs and staff at the same clinics (N=15)	To explore patient and clinic-level perceptions of the use of depression self-management apps within an integrated primary care setting	Semistructured focus groups; semistructured interviews
Forma et al [16], 2022	Cross-sectional, quantitative	Caregivers of patients with BD, MDD, or schizophrenia who believed their patients had adherence issues to second-generation oral atypical antipsychotic medication (N=184)	To assess caregivers’ preferences and willingness to pay for digital (ingestible sensor pill, medication containers with electronic monitoring, mobile apps, and smart pill dispensers) and nondigital (medication diary and simple pill organizer) tools	Custom discrete choice experiment survey
Hoffman et al [5], 2019	Prospective interventional, mixed methods	HCPs (N=24) in a routine primary care behavioral health setting who reported their own and patients’ (sample size not reported) MH app use and feedback; patient conditions included anxiety, stress, depression, and substance use	To test the feasibility of using mHealth apps to augment integrated primary care services, solicit feedback from patients and providers to guide implementation, and develop an MH app toolkit for system-wide dissemination	Custom survey
Huberty et al [28], 2022	Cross-sectional (current Calm (Calm.com, Inc) users) and prospective interventional (nonusers of Calm, HCPs), qualitative	General users^f^: patients with cancer and survivors of cancer with smartphones, some of whom were current subscribers of Calm, a meditation app (N=17) HCPs, staff, and not-for-profit partners in cancer care with smartphones (N=10)	To develop a mobile meditation app prototype specifically designed for patients with cancer and survivors of cancer	Custom surveys; focus groups
Kern et al [29], 2018	Cross-sectional, quantitative	General users^f^: students from a midwestern university with smartphones (N=721)	To investigate the potential usefulness of MH apps and attitudes toward using them	Custom survey
Knapp et al [30], 2021	Prospective cohort, qualitative	Clinical staff members who provide behavioral health care for children and adolescents with conditions, including ADHD and depression, at a large community service organization in a midwestern state (N=37)	To learn about considerations and perspectives of community behavioral HCPs on incorporating digital tools into their clinical care for children and adolescents	Focus groups
Kornfield et al [31], 2022	Prospective cohort, qualitative	Users: participants with at least moderate levels of depression or anxiety symptoms on the PHQ-9^m^ or GAD-7^n^ questionnaires, but without serious mental illnesses (eg, BD, schizophrenia), who were not receiving formal care and recruited upon completing free web-based MH self-screening surveys hosted by Mental Health America (N=28)	To investigate how digital technologies can engage young adults in self-managing their MH outside the formal care system	Web-based asynchronous discussion; synchronous web-based design workshop
Lipschitz et al [32], 2019	Cross-sectional, quantitative	Users: veterans enrolled in care at the VA Boston Healthcare System diagnosed with an anxiety disorder (including OCD), unipolar depressive disorder, or PTSD and who had at least 1 encounter in the local primary care clinic (N=149)	To assess patients’ interest in mHealth interventions for MH, to identify whether provider endorsement would impact interest, to determine reasons for nonuse of mHealth interventions for MH, and to identify what mHealth content or features are of most interest to patients	Custom survey
Mata-Greve et al [33], 2021	Cross-sectional, mixed methods	General users^f^: essential workers during the COVID-19 pandemic or workers who were unemployed or furloughed because of the COVID-19 pandemic, recruited from a web-based research platform (N=1987)	To document psychological stress, to explore DMHT^o^ use in response to COVID-19–related stress, to explore the usability and user burden of DMHTs, and to explore which aspects and features of DMHTs were seen as necessary for managing stress during a pandemic by having participants design their own ideal DMHTs	Survey combining custom and validated measures (System Usability Scale, Use Burden Scale)
Melcher et al [34], 2022 and Melcher and Torous [4], 2020	Cross-sectional, mixed methods	General users^f^: college students aged 18-25 years, recruited through social media and word of mouth (N=100)	To examine why college students show poor engagement with MH apps and how apps may be adapted to suit this population	Custom survey; interviews
Schueller et al [35], 2018	Cross-sectional, mixed methods	General users^f^: smartphone owners recruited from a research registry (N=827)	To understand where users search for MH apps, what aspects of MH apps they find appealing, and what factors influence their decisions to use MH apps	Custom survey; focus group interviews
Schueller et al [36], 2021	Cross-sectional, qualitative	General users^f^: participants who had used an app that allowed them to track their mood, feelings, or mental well-being for ≥2 weeks, recruited from a research registry (N=22)	To understand motivations for and experiences in using mood-tracking apps from people who used them in real-world contexts	Semistructured interviews
Stiles-Shields et al [37], 2017	Cross-sectional, qualitative	General users^f^: participants recruited from web-based postings; approximately equal numbers of participants were above and below the criteria for a referral for psychotherapy for depression (N=20)	To identify the barriers to the use of a mobile app to deliver treatment for depression and to provide design implications on the basis of identified barriers	Card sorting task
Storm et al [38], 2021	Cross-sectional, qualitative	Patients with diagnoses of schizophrenia, schizoaffective disorder, BD, or persistent MDD in active treatment at a community MH center (N=17) Peer support specialists at the same center (N=15)	To identify stakeholders’ perspectives on partnering to inform the software development life cycle of a smartphone health app intervention for people with serious mental illness	Semistructured interviews
Torous et al [39], 2018	Cross-sectional, quantitative	Outpatients attending psychiatric clinics; one clinic primarily treated mood and anxiety disorders, and the other primarily treated psychotic disorders (N=185)	To understand how individuals with mental illness use their mobile phones by exploring their access to mobile phones and their use of MH apps	Custom survey
Zhou and Parmanto [40], 2020	Cross-sectional, mixed methods	Users: participants with mild or moderate depression with local privacy concerns when using MH apps, recruited from a research registry (N=40)	To determine user preferences among the several privacy protection methods used in current mHealth apps and the reasons behind those preferences	Custom survey; interview

^a^Only information relevant to this systematic literature review is reported in this table.

^b^MDD: major depressive disorder.

^c^BD: bipolar disorder.

^d^OCD: obsessive-compulsive disorder.

^e^MH: mental health.

^f^General users are participants who were not necessarily diagnosed with indications of interest.

^g^ASD: autism spectrum disorder.

^h^HCP: Health care provider.

^i^mHealth: mobile health.

^j^PTSD: posttraumatic stress disorder.

^k^VA: Veterans Affairs.

^l^ADHD: attention-deficit/hyperactivity disorder.

^m^PHQ-9: Personal Health Questionnaire-9.

^n^GAD-7: Generalized Anxiety Disorder-7.

^o^DMHT: digital mental health technology.

Most frequently, studies focused on indications for mood, anxiety, or psychotic disorders (15/26, 58%), with other indications of focus including ADHD (2/26, 8%), ASD (1/26, 4%), and epilepsy (1/26, 4%). No relevant studies focused on Alzheimer disease were identified.

A total of 8 (31%) studies assessed the perspectives toward DMHTs of general population participants who were not necessarily diagnosed with relevant conditions [19,28,29,33-37]. Of these populations, several were identified as having an increased risk of MH conditions, such as patients with cancer [28], college students [29,34], deaf or hard-of-hearing individuals [19], and people who were unemployed or furloughed during the COVID-19 pandemic [33]. In addition, 1 (4%) study included a mix of patients who were above and below the referral criteria for psychotherapy for depression [37].

### Thematic Analysis

#### Overview

Evidence was identified for all 5 themes included in the APA framework: engagement style (23/26, 88%), background and accessibility (16/26, 62%), privacy and security (13/26, 50%), therapeutic goal (12/26, 46%), and clinical foundation (8/26, 31%; Table 2). Five novel criteria were identified and added to the framework post hoc, 1 each under engagement style (forgetting or feeling unmotivated to use DMHTs) and privacy and security (personal image and stigma) and 3 under background and accessibility (willingness to pay, insurance restrictions, and cost savings compared with professional care).

**Table 2:** Studies reporting on each theme, subtheme, and criterion.

Subtheme			Criteria (study reference)
**Engagement style**			
	Short-term usability	Ease of use [5,25,34,35] Available engagement styles [20-22,24,25,30,31,34,37]	
	Long-term usability	Alignment of app with needs and priorities [5,16-22,24,26,28-34,36-39] Forgot or unmotivated to use^a^ [5,25,31,37]	
	Customizability	No further stratification [20,24,28,31,32,34,35]	
**Background and accessibility**			
	Technical	Offline functionality [19,23,25,27,30,37] Compatibility with different operating systems^b^ Accessibility [5,19,25,27-29,32,35,38]	
	Business model^b^	Funding sources or conflicts of interest^b^	
	Costs	Additional or hidden costs [26,37] Willingness to paya [16,25,34,35] Insurance restrictions^a^ [20] Cost savings compared with professional care^a^ [29] No further stratification [25-27,34]	
	Medical claims^b^	Specific medical claims^b^ Trustworthiness of source^b^	
	Stability	Frequency of software updates [35,37]	
**Privacy and security**			
	No specific subtheme	No further stratification [34,36,37]	
	Data collection and storage	Ability to opt out of data collection or delete data^b^ Data storage location^b^ Security associated with collection, use, and transmission of sensitive data (including personal health information) [5,19,27,29,32]	
	Privacy policy	Transparency and accessibility of privacy policy [34,35,38] Declaration of data use and purpose [34] Data sharing with third parties [25] Systems to respond to potential harms or safety concernsb	
	Personal health information	Description of use of personal health information [34] Personal image and stigmaa [5,25,29,40]	
	Security measures	Security systems used [30,35,40]	
**Clinical foundation**			
	Impressions of use	Accuracy and relevancy of app content [25,26] Alignment in app appearance and its claimed purpose^b^	
	User feedback	Evidence of specific benefit from user feedback or user research studies [27,35] Validation of app usability and feasibility^b^	
	Clinical validity	Supporting sources or references for use cases of the app [34,35] Evidence of specific benefit [27,34-36] Evidence of effectiveness or efficacy [32,34,37] Clarity in functional scope^b^	
**Therapeutic goal**			
	Clinically actionable	Positive change or skill acquisition [5,18,26-28,30,31,34,36] Ease of sharing and interpretation of data [25-27,30]	
	Therapeutic alliance	Possibility for collaboration with an HCPc [5,21,22,27,34,36] The therapeutic alliance between patient and HCP [5,26]	
	Data ownership, access, and export^b^	User ownership of data^b^ Opportunity for sharing of data with electronic medical records and other data tools (Apple HealthKit, Fitbit)^b^ Opportunity for use with a provider and ability to export or transfer data^b^	

^a^Novel findings that emerged from this systematic literature review.

^b^These subthemes and criteria were included in the American Psychiatric Association’s framework but were not reported on by studies included in this systematic literature review.

^c^HCP: health care provider.

#### Theme 1: Engagement Style

Engagement style was the most reported theme, with evidence identified from 23 (88%) of the 26 studies. Engagement style encompasses how and why users do or do not interact with DMHTs. The long-term usability subtheme was reported by 96% (22/23) of studies, short-term usability by 12 (52%) studies, and customizability by 7 (30%) studies. Findings from short- and long-term usability subthemes were highly interconnected.

A total of 4 studies reported that ease of use promoted short-term DMHT engagement. In the study by Schueller et al [35], 89.6% of a general population of smartphone users reported ease of use for MH apps as “important” or “very important,” and users qualitatively reported dislike of “overwhelming,” difficult-to-navigate apps. In addition, users valued apps that were “simplistic” [34], fit into their daily schedules, and were available when needed (eg, during acute symptom experiences) [5,25]. Select supporting qualitative data are presented in Table 3.

**Table 3:** Select key quotes identified for systematic literature review findings.

Subtheme and criteria: findings				Key quotes
**Engagement style**				
	**Short-term usability**			
		Ease of use	“I like short exercises. I can use them in different places.” [Patient in routine behavioral health care] [5] “Whenever I have one of those outbursts and frustration, I can just open it up, say ‘Okay, what’s my first step?’” [Male veteran, aged 26 years] [25]	
		Available engagement styles: use of animation and visuals	“They love badges. And decorating their avatars, like getting a new hat...So, they’re very motivated to get through their modules when they get to earn something at the end.” [Pediatric behavioral health clinician] [30] “It could become visually distracting—children preferring the animation rather than actually creating genuine, communicative messages.” [Caregiver or speech-language pathologist for children with ASD^a^] [20]	
	**Long-term usability**			
		Alignment of app with needs and priorities: gamification	“I’ve seen some kid clients come alive because they’re excited because they wanna beat their score. And just helping them like, ‘How do you have to communicate? You have to keep talking. You have to keep going.’ It’s helped with that.” [Pediatric behavioral health clinician] [30]	
		Alignment of app with needs and priorities: anxiety management	“App features that could help to reduce anxiety, for example, guided meditation, breathing exercises, or positive affirmation [may be] useful.” [Community MH^b^ center peer support specialist] [38] “Stuff that’s purely motivational...can feel alienating if I’m depressed...but focusing on something specific, like doing a breathing exercise...would be cool.” [Patient with anxiety or depression] [31]	
		Alignment of app with needs and priorities: tracking mood, symptoms, or sleep	“They [the adolescent] can bring it up on their phone...and we look at just is she daily fluctuating? If so, what happened during that day?” [Pediatric behavioral health clinician] [30] “I don’t know...if he’s good or he’s getting better or worse or anything like that. Just everything being simple in one place, and just hit a couple of buttons and not have to write anything down will be very good.” [Caregiver of a child with ADHD^c^] [26]	
		Alignment of app with needs and priorities: social media–like features	“I like hearing other people’s stories and what they did, and it kind of helps me feel a little better. And I kind of like bounce off it and do what they did and try these new things that they’re doing.” [User with anxiety or depression] [31]	
		Alignment of app with needs and priorities: peer support and chat functions	“Incorporating lived experiences into a [smartphone] app and organize the [intervention] process to address lived experience because that’s what it’s all about.” [Community MH center peer support specialist] [38] “So maybe the bipolar individual also has access to the same app and then so they talk to each other...That way when I get home, I know ahead of time, it was an okay day today...Or if it was not a good day ok, so I know that I need to come in a little more reserved.” [Spouse of an individual with BD^d^] [24]	
		Forgot or unmotivated to use^e^	“For someone who may be severely depressed, or someone who needs help, [writing messages] is almost like hard to do. Because if they’re having a hard time motivating or encouraging themselves, they might not feel like this is something they could do.” [User with anxiety or depression] [31] “I notice a good number of patients mentions they did not continue using in home. [...] Maybe because this area is still new for patients?” [Routine behavioral health care staff] [5] “[My son] has one of those crazy little phones that you can do everything with. I just don’t have an interest.” [Female veteran aged 57 years] [25]	
**Background and accessibility**				
	**Technical considerations**			
		Accessibility: mobility barriers	“They can’t figure out why my hands shake so bad...so trying to use a smartphone [is frustrating]...I don’t have a whole lot of feeling in my hands.” [Male veteran aged 40 years] [25]	
		Accessibility: technical literacy	“I haven’t gotten acclimated to a smartphone yet...the technology is kind of difficult to navigate.” [Male veteran aged 66 years] [25]	
		Offline functionality: internet and mobile data access as a barrier to use	“[A young person’s smartphone] is normally one of the first things that get taken away if they do have a bad day. So, this is the thing you can use when you’re having a bad day to calm down, but then mom and dad won’t let you use it because you had a bad day.” [Pediatric behavioral health clinician] [30] “There have been times I think people have suggested, ‘Check this app out, check that app out,’ and for the most part I don’t think I have...I do only have so much data.” [Patient receiving psychiatric care] [23]	
	**Costs**			
		Willingness to pay^e^	“If they don’t have the free trial and they want money, I’m not even gonna look at it. I’m not gonna pay for something before I’ve gotten the chance to see if it’s gonna work for me or not.” [General user, smartphone owner] [35] “...[T]hey gave the option to pay $50.00 a year. And I did that, because I liked the idea of what they were trying to do.” [General user, smartphone owner] [35]	
**Privacy and security**				
	**Data collection and storage**			
		Security associated with collection, use, and transmission of sensitive data (including personal health information)	“I’m worried about my data.” [Patient in routine behavioral health care] [5] “Any apps that terms and conditions you’re forfeiting your information as soon as you click to that to anything so and I’m not worried about getting identity theft.” [Patient with current or prior depression diagnosis] [27]	
	**Privacy policy**			
		Transparency and accessibility of privacy policy	“To use a smartphone app with a client I would want to make sure it’s secure before going any further.” [Community MH center peer support specialist] [38]	
	**Personal health information**			
		Personal image and stigma^e^	“I worry about my virtual image. I’d feel more comfortable using an app from CHA^f^ that is protected in the same way my EMR^g^ is protected.” [Patient in routine behavioral health care] [5]	
	**Security measures**			
		Security systems used	“The app doesn’t read as something like, My Personal Diary...it reads as something that you might just pass by if you don’t know what its intention is, which can be good for teenagers who are afraid of people looking into their stuff.” [Pediatric behavioral health clinician] [30]	
**Therapeutic goal**				
	**Clinically actionable**			
		Positive change or skill acquisition: apps that impart skills and encourage positive change, in an easy way	“Great way to have patients practice exercises between sessions; both provider and patient happy to have concrete tool.” [Routine behavioral health care staff] [5] “I almost wonder, like, if you logged in, what would you like to address today, like, symptom management versus stress...You almost need, like, an emergency toolkit and then you almost need, like, your day-to-day stuff.” [HCP^h^ in cancer care] [28]	
		Ease of sharing and interpretation of data: increase of engagement and symptom reporting	“[I] feel like sometimes I’ll give parents follow up things to do while I’m not there, and they’ll forget about it throughout the week, but because they’re on their phone or whatever so much throughout the week, I feel like we could send them reminders or this is what we need to do before the next week. I think that that would encourage them to be more engaged, at least in the process.” [Pediatric behavioral health clinician] [30]	
	**Therapeutic alliance**			
		Therapeutic alliance between patient and HCP	“Sometimes I think my training in behavioral medicine allows me to create a different tool with the patient that is more specific to them.” [Routine behavioral health care staff] [5]	
**Clinical foundation**				
	**Clinical validity**			
		Evidence of specific benefit: HCP recommendations	“My doctor tells me to use an app, I’m probably going to use it.” [Patient with current or prior depression diagnosis] [27]	
		Evidence of specific benefit: increased usage if supported by research, academic institution, or reputable professional society	“I would trust an app supported by my university more than a random app I found online.” [General user, college student] [34] “I think it would be helpful, too, to have like the American Psychiatric Association or something, one of those, the licensure bodies or whatever—if they had official recommendations or backing.” [General user, smartphone owner] [35]	

^a^ASD: autism spectrum disorder.

^b^MH: mental health.

^c^ADHD: attention-deficit/hyperactivity disorder

^d^BD: bipolar disorder.

^e^Novel criteria identified by this systematic literature review.

^f^CHA: Cambridge Health Alliance.

^g^EMR: electronic medical record.

^h^HCP: health care provider.

Users valued DMHT features that aligned with their needs and priorities, as reflected by findings within the long-term usability subtheme. Across 9 studies, quantitative and qualitative findings demonstrated high interest in anxiety management features such as relaxation tools, breathing exercises, and mindfulness or meditation activities, and 10 studies identified interest in mood, symptom, or sleep tracking (Tables 3 and 4). While most studies (24/26, 92%) focused on MH, patients with epilepsy also reported high interest in features to record seizure dates and types [17]. Importantly, users in 2 studies emphasized the need for developers to tailor DMHTs to the needs and priorities of the target population (Table 3) [28,31]. Relatedly, mixed attitudes were reported toward positive affirmations and words of encouragement, with many users expressing interest but others emphasizing the value of a human component to DMHTs or cautioning against blanket encouragement and automated messages that could feel insincere [19,25,31].

**Table 4:** Quantitative evidence related to anxiety management and mood, symptoms, or sleep tracking features.

Features, study, perspective, and finding					Patients, n (%)		Likert score, mean (SD)
**Anxiety management**							
	**Buck et al** **[22], 2021b**						
		**Young adults with early psychosis**					
			Interest in skill practices for managing stress and improving mood	64 (84.2)		3.30 (0.98)^a^	
			Interest in skill practices for relaxation	57 (76)		3.09 (1.12)^a^	
			Interest in information about relaxation exercises	59 (77.6)		3.00 (1.16)^a^	
			Interest in information about healthy sleep practices	56 (73.7)		2.93 (1.15)^a^	
			Interest in mindfulness or meditation practices	44 (59.4)		2.61 (1.34)^a^	
	**Afra et al [17], 2018**						
		**Patients with epilepsy**					
			Interest in music to help seizure control	—^b^ (75)		—	
			Interest in relaxing music that may help alleviate stress	— (68)		—	
			Interest in relaxing imagery that may help alleviate stress	— (40)		—	
			Interest in drawing or writing while listening to music	— (35)		—	
			Interest in practicing mindfulness	— (63)		—	
	**Torous et al [39], 2018**						
		**Outpatients attending a private psychiatric clinic**					
			Comfort level for mindfulness and therapy	—		3.75^c^	
		**Outpatients attending a state psychiatric clinic**					
			Comfort level for mindfulness and therapy	—		3.17^c^	
	**Beard et al [18], 2019**						
		**Patients in a partial hospitalization program in a psychiatric hospital**					
			Current use of an MH^d^ app with the primary purpose being mindfulness or meditation	— (71)		—	
	**Mata-Greve et al [33], 2021**						
		**Workers furloughed during COVID-19**					
			Most frequently endorsed mindfulness tools as a feature when provided options to build their own app	687 (67.8)		—	
		**Essential workers employed during COVID-19**					
			Most frequently endorsed mindfulness tools as a feature when provided options to build their own app	584 (60)		—	
		**Nondistressed essential workers employed or workers furloughed during COVID-19**					
			Most frequently endorsed mindfulness tools as a feature when provided options to build their own app	305 (61.4)		—	
		**Distressed essential workers employed or workers furloughed during COVID-19**					
			Most frequently endorsed mindfulness tools as a feature when provided options to build their own app	966 (65.3)		—	
	**Hoffman et al [5], 2019**						
		**Staff in a routine primary care behavioral health setting**					
			The ability to manage mood, anxiety, or substance use through the use of DMHTs^e^ was seen as a benefit of incorporating DMHTs into clinical care	13 (57)		—	
**Symptom, mood, or sleep trackers**							
	**Kern et al [29], 2018**						
		**General population of college students**					
			Willingness to use an MH app to track mood or anxiety	41 (10.3)		—	
	**Afra et al [17], 2018**						
		**Patients with epilepsy**					
			Interest in a diary to record the date of seizures	— (85)		—	
			Interest in a digital diary to record the type of seizure	— (73)		—	
			Interest in digital diary to log the missed dosages of their medications	— (78)		—	
	**Lipschitz et al [32], 2019**						
		**Veterans with anxiety, MDD^f^, or PTSD^g^**					
			Interested in progress monitoring (track mood, stress, anxiety, or PTSD symptoms)	95 (63.8)		—	
		**Subgroup of smartphone owners**					
			Interested in progress monitoring (track mood, stress, anxiety, or PTSD symptoms)	80 (67.2)		—	
	**Buck** **et al** **[22], 2021b**						
		**Young adults with early psychosis**					
			Interest in a feature to set and track goals	60 (78)		3.10 (1.05)^a^	
			Interest in a feature to track symptoms over time	70 (90.9)		3.44 (0.90)^a^	
			Interest in a feature to track changes in progress toward goals	66 (86.9)		3.37 (0.86)^a^	
			Interest in a feature to track wellness behaviors (eg, steps or activity)	48 (64.9)		2.86 (1.22)^a^	
	**Beard et al [18], 2019**						
		**Patients in a partial hospitalization program in a psychiatric hospital**					
			Current use of an MH app with the primary purpose being mood tracking	— (10)		—	
			Willingness to use an MH app daily to monitor condition	262 (81)		—	
		**Subgroup with higher education**					
			Willingness to use an MH app daily to monitor condition	— (85)		—	
		**Subgroup with lower education**					
			Willingness to use an MH app daily to monitor condition	— (77)		—	
	**Mata-Greve et al [33], 2021**						
		**Workers furloughed during COVID-19**					
			Most frequently endorsed symptom tracking (tracking sleep or mood) as a feature when provided options to build their app	605 (59.7)		—	
		**Essential workers employed during COVID-19**					
			Most frequently endorsed symptom tracking (tracking sleep or mood) as a feature when provided options to build their app	555 (57)		—	
		**Nondistressed essential workers employed or workers furloughed during COVID-19**					
			Most frequently endorsed symptom tracking (tracking sleep or mood) as a feature when provided options to build their app	270 (54.3)		—	
		**Distressed essential workers employed or workers furloughed during COVID-19**					
			Most frequently endorsed symptom tracking (tracking sleep or mood) as a feature when provided options to build their own app	890 (60.2)		—	
	**Torous et al [39], 2018**						
		**Outpatients attending a private psychiatric clinic**					
			Comfort level for in-app symptom surveys	—		3.50^c^	
		**Outpatients attending a state psychiatric clinic**					
			Comfort level for in-app symptom surveys	—		3.11^c^	
		**Outpatients attending a private psychiatric clinic**					
			Comfort level for passive call or text monitoring	—		2.32^c^	
		**Outpatients attending a state psychiatric clinic**					
			Comfort level for passive call or text monitoring	—		2.39^c^	
		**Outpatients attending a private psychiatric clinic**					
			Comfort level for passive GPS monitoring	—		2.31^c^	
		**Outpatients attending a state psychiatric clinic**					
			Comfort level for passive GPS monitoring	—		2.78^c^	

^a^A 5-point Likert scale (0-4) was used.

^b^Not available.

^c^A 5-point Likert scale (1-5) was used.

^d^MH: mental health.

^e^DMHT: digital mental health technology.

^f^MDD: major depressive disorder.

^g^PTSD: posttraumatic stress disorder.

Both patients and caregivers expressed interest in psychoeducational content that aligned with their needs and priorities. When surveyed, >60% of veterans with anxiety or major depressive disorder (MDD), patients with epilepsy, young adults with psychosis, and essential and furloughed workers during the COVID-19 pandemic expressed interest in relevant psychoeducational content [17,22,32,33]. In contrast, only 4% of college students in another study reported using an MH app for information about MH, although an MH diagnosis was not required for study participation [29].

Caregivers of young adults with psychosis, caregivers of children with ADHD, and spouses and partners of people with bipolar disorder (BD) were all interested in information related to caring for the individual with the given disorder, such as information on psychological and pharmacological treatments, symptoms and symptom changes, and the MH system [21,24,26]. Comparatively smaller, but still notable, proportions of caregivers of patients with psychosis were interested in caregiver-focused information; for instance, 62% to 69% were interested in relaxation exercises, stress and mood management, and community events for caregivers, while 85% to 90% were interested in the aforementioned patient-focused information [21].

Information delivery–style preference was captured under the short-term usability subtheme. One study in young adults with psychosis and another study with their caregivers revealed that delivering information in a variety of formats was important; when presented with nonmutually exclusive options, >50% of both populations were interested in text content, video content, audio content, and discussion boards [21,22].

Social interaction promoted long-term engagement. Qualitatively, 3 studies found that users valued learning about similar experiences from others via social media–like features, which normalized their experiences and could provide new symptom management strategies (Table 3) [28,31,36]. Similarly, 67% of both young adults with psychosis and deaf or hard-of-hearing survey participants (N=9) reported interest in peer support via chat features [19,22]. However, a comparatively smaller proportion of veterans with anxiety or MDD (48.3% of the full cohort and 51.3% of the smartphone user subgroup) were interested in peer support [32].

Overall, users endorsed social features to support their MH. In the study by Casarez et al [24], spouses and partners of people with BD likewise desired features to communicate with other caregivers and also emphasized that DMHTs could facilitate conversation and understanding with patients, a sentiment echoed by peer support specialists by Storm et al [38] (Table 3). However, one oncology HCP cautioned that similar to support groups, “very strict guidelines of what is said” should be implemented to manage potential risks from shared social media–like content, although little additional context was provided [28].

Spouses and partners of people with BD also suggested both in-app information on accessing professional resources and direct counseling for the patient at times when other support might be inaccessible [24]. More than half of all workers, employed or unemployed during the COVID-19 pandemic, likewise endorsed links to resources, counseling, and crisis support as DMHT features, and 81.6% of young adults with psychosis endorsed a feature to communicate with professional experts [22,33]. Importantly, compared with patients attending public clinics, patients attending private psychiatric clinics expressed a higher comfort level for in-app communication with HCPs, suggesting demographic differences in the valuation of access to professional support through DMHTs [39].

A total of 9 studies reported an interest in DMHT reminders and notifications. Across 3 studies, >70% of patients or caregivers were interested in appointment reminders [17,21,22]. In addition, 73% and 68% of patients with epilepsy reported interest in reminders for medication refills and adherence, respectively [17]. Beyond apps, caregivers of patients with MDD, BD, and schizophrenia preferred an ingestible pill sensor that tracked medication adherence, physical activity, mood, and rest 9.79 (95% CI 4.81-19.9), 7.47 (95% CI 3.81-14.65), and 6.71 (95% CI 3.29-13.69) times more than a nondigital pill organizer, respectively [16]. Qualitatively, patients and caregivers also appreciated reminders, especially if reasonably timed or delivered via text messages [27,31].

Short-term DMHT engagement was also supported by games and graphics, which could communicate information in an accessible way [24], provide tools for stress management [17,33], and be used therapeutically with children [20,30]. However, some HCPs and caregivers expressed concerns that graphics and games may be distracting for certain children (Table 3) [20].

In a novel finding, 3 studies reported forgetfulness or lack of motivation as an influence on DMHT engagement. In some cases, disuse was related to stress, other MH symptoms, or poor technical literacy (Table 3) [5,25,31]. In contrast, “forgetting to use” DMHTs and “lack of motivation” were perceived as relatively small barriers to use in the study by Stiles-Shields et al [37].

The third subtheme under engagement style was customizability, which was generally valued by users; 70.9% of a general population of smartphone users noted customization was an important factor [35]. Similarly, 9.4% of all surveyed veterans and 10.9% of those with smartphones reported disliking a prior DMHT due to a lack of personalization [32]. Users specifically wanted to be able to opt out of irrelevant features, customize audiovisual and design elements, add personal notes to tracked mood data, and provide ongoing feedback to facilitate personalization [20,24,28,31,34].

#### Theme 2: Background and Accessibility

A total of 16 (62%) studies reported findings related to DMHT background and accessibility, which considers the developer of the DMHT, as well as functionality and accessibility. Of these, 12 (75%) studies reported on the technical considerations subtheme, 9 (56%) on costs, and 2 (13%) on stability.

Under technical considerations, 9 studies assessed diverse accessibility concerns. Broadly, Storm et al [38] emphasized that DMHTs should be developed in consideration of patients’ social, cognitive, and environmental needs to avoid overwhelming users. Specifically, 2 studies reported language as a barrier. Deaf or hard-of-hearing participants recommended visual content presentation, such as videos and icons, alongside text and American Sign Language translations where possible [19]. Similarly, when discussing English-only apps, 1 provider stated as follows: “language is a barrier for some [patients]” [5]. Mobility issues related to MH symptoms or other conditions and technical literacy, such as difficulties remembering passwords and navigating smartphones or apps, created accessibility barriers as well (Table 3) [5,25,27,28]. Additional concerns included apps that restricted use based on geographic location [19], user difficulty in finding relevant, useful apps [32], and limited mobile device memory for downloading apps [5,19].

Offline functionality, reported by 6 studies, was also captured under the technical considerations subtheme. A majority (5/9, 56%) of participants included in the study by Borghouts et al [19] expressed concern about their mobile data plans when using their devices. Correspondingly, “availability of Wi-Fi” was noted as a top barrier to the use of apps for depression by Stiles-Shields et al [37], and several veterans in another study reported that home Wi-Fi connectivity facilitated app use by eliminating cellular data fees [25,37]. Quotes from patients and HCPs echoed the concern about apps without offline functionality (Table 3) [23,30].

Data fees were also captured under the costs subtheme, with hidden or additional costs described as a barrier to app use by 2 studies [26,37]. Parents of children with ADHD reported that difficulty paying phone bills could result in their phones being shut off, limiting DMHT use; one MH clinic administrator stated as follows: “We often encounter parents’ phones being shut off because they haven’t paid their bill...If the app were free or low cost, I imagine it could be very helpful” [26]. In addition to hidden costs, this quote identifies up-front app costs as a barrier. Quantitatively, more than half of a general population of surveyed college students expressed that cost was a top concern for the use of MH apps [34]. Qualitative findings from 2 additional studies likewise identified cost as a barrier to DMHT use [25,27].

Three novel cost attributes were identified by this SLR: willingness to pay, insurance restrictions, and cost savings compared with professional care. Four studies, 3 of which focused on apps, explored willingness to pay for DMHTs from a user perspective. Willingness to pay varied based on user preference; some surveyed college students and smartphone users among general populations valued free apps due to financial restrictions or uncertainty around app effectiveness, although 1 student commented that the quality of free trials might be inferior [34,35]. Some smartphone users also voiced a limit on how much they would be willing to spend for an app subscription (Table 3) [35]. Forma et al [16] found that caregivers were willing to pay US $255.04 (95% CI US $123.21-US $386.86) more per month for a pill with an ingestible sensor that tracked medication adherence, physical activity, and rest and could connect to an app that also collected self-reported mood data. Moreover, the caregivers were willing to pay US $124.50 (95% CI US $48.18-US $200.81) more per month for an app-connected pill organizer alone than for a nondigital pill organizer [16]. In contrast, some veterans expressed total disinterest in paid apps, with 1 user citing poor technical literacy (“don’t have the knowledge”) in addition to cost as affecting willingness to pay [25].

In another novel finding, a speech-language pathologist working with children with ASD preferred a single app including multiple features over separate apps for particular features due to insurance restrictions: “I agree that teaching Apps should be an in-App feature versus their own app because sometimes insurance doesn’t allow us to open the iPads purchased through insurance” [20]. Although no further detail was provided for this finding, it suggests that there may be restrictions on the use of other apps on devices purchased under insurance, which may have implications for DMHT use in formal care settings due to the lack of financial support.

In a third novel cost-related finding, a small number of participants from a general population of students (3.6%) in one study preferred using an MH app to seeing an MH professional due to cost savings [29].

A total of 13% (2/16) of studies reported on the subtheme of app stability and technical difficulties, with crashes and poor display quality decreasing DMHT value [35,37]. Participants in the study by Schueller et al [35] reported that technical difficulties were often an issue for apps developed by medical institutions, which might be effective and safe but less usable than apps from other developers.

#### Theme 3: Privacy and Security

A total of 13 (50%) out of 26 studies reported findings related to the privacy and security theme, which covered the use and protection of user data by DMHTs. Subthemes were reported relatively equally: data collection and storage (5/13, 38%), personal health information (PHI; 5/13, 38%), privacy policies (4/13, 31%), general privacy (3/13, 23%), and security measures (3/13, 23%).

Quantitative and qualitative findings on general privacy (ie, evidence not categorized under any specific subtheme), the data collection and storage subtheme, and the privacy policies subtheme revealed heterogeneous concerns (Table 3). A total of 74% of a general population of college students reported privacy as a top concern for MH apps, although further details on the specific area of concern were unclear [34]. In the study by Stiles-Shields et al [37], participants were highly concerned with data access but less so with general privacy. Echoing the concerns about data collection and storage, 59.1% of veterans with anxiety or MDD in 1 study were concerned about in-app PHI protection [32]; however, a qualitative study in veterans with posttraumatic stress disorder, alcohol use disorder, or MDD reported that a relatively small number of participants expressed privacy concerns. In the latter study, reasons for the concerns included distrust in Veterans Affairs, belief that digital data are inherently not confidential, and fear of phone hacking [25]. From an HCP perspective, none of the surveyed behavioral health HCPs agreed with the statement “My patients are concerned about data security,” despite multiple patients within the same study reporting privacy concerns [5].

Still, privacy policies were important overall, with 70.5% of smartphone MH app users rating having a privacy policy as “very important” or “important” [35]. Melcher et al [34] found that although users valued data protection, some reported a lack of awareness about data privacy, and others were concerned about obscure privacy policies and PHI use. As noted in the data collection and storage subtheme, veteran concerns about government use of PHI were heterogeneous [25].

A novel valuation factor not included in the APA framework related to user concern with PHI privacy and security regarding MH diagnoses and MH app use is a desire to upkeep their personal image or avoid stigma (Table 3) [5,25,29,40]. For instance, 21.1% of a general college student population preferred MH app use to seeing an MH professional due to anonymity or reduced stigma [29]. One participant in a study of Veterans Affairs health service users described access to professional care via MH apps as convenient because they could avoid disclosing their use of MH services to explain leaving work early for an appointment [25].

In line with the overarching concern about PHI privacy and security, users valued app security measures. Schueller et al [35] reported that 74.2% of users rated data encryption as “important” or “very important.” Users in another study perceived the level of privacy protection as the highest for apps using a combination of a generic app name (ie, not reflecting the indicated MH disorder); easily hidden modules; and secure, user-authenticated web portals for making module changes [40]. Behavioral health clinic staff echoed the importance of discreet MH app names (Table 3) [30].

#### Theme 4: Therapeutic Goal

There were 12 (46%) studies that reported on the factors relating to the integration of DMHTs with users’ therapeutic goals. The clinical actionability and therapeutic alliance subthemes were reported by 83% (10/12) and 58% (7/12) of studies, respectively.

A total of 9 studies reported the value of clinically actionable insights from apps where the users could acquire and practice new skills to make positive changes in their lives (Table 3). For instance, patient and caregiver app users reported interests in “daily tips,” “new ideas,” and “solutions or recommendations” for symptom management [26,27,36]. Furthermore, an app that could serve as a resource for multiple management strategies was preferable [26,28,31]. Quantitatively, 4% of patients receiving acute treatment in a partial hospitalization program for MH conditions, including mood and psychotic disorders, reported that the primary purpose of their DMHT use was therapy skills practice [18]. HCPs similarly appreciated that DMHTs could facilitate patients practicing skills outside of formal treatment sessions [5]. In particular, clinicians from a youth behavioral health clinic noted that DMHTs might be especially beneficial for young users because they could be conveniently and discreetly incorporated into their daily lives [30].

Users valued easy data sharing with clinicians, particularly for mood- or symptom-tracking features, which could improve communication and the accuracy of symptom reporting during clinical visits [5,25-27,34,36]. For instance, 53% of a general college student population believed that the potential to share information with their clinician was “one of the top benefits” of using DMHTs [34]. In addition, many HCPs reported active use or interest in the use of DMHTs in clinical practice to facilitate asynchronous communication and increase patient engagement with treatments outside of formal appointments; however, some preferred traditional care strategies for their personalization and flexibility (Table 3) [5,26,30].

#### Theme 5: Clinical Foundation

A total of 8 (31%) studies reported findings related to the clinical foundation of DMHTs, that is, their utility and appropriateness for patients. Clinical validity was the most reported subtheme, with evidence identified from 6 (75%) studies; 2 (25%) studies reported on the user feedback subtheme and 2 (25%) on the impressions of use subtheme, which captured users’ perceptions of app content as accurate and relevant.

Across subthemes, users valued evidence of DMHT benefit or efficacy from various sources. A total of 71.8% of surveyed veterans said that they would use a DMHT if they “saw proof that it worked” for their MH conditions [32]. Similarly, among the 811 general population participants surveyed, 69.5% ranked direct research evidence as “important” or “very important” for DMHT, and 66.8% ranked indirect research evidence the same [35]. Qualitative data identified recommendations from HCPs or academic institutions, as well as evidence of DMHT benefit from publications or research studies, as specific sources for clinically valid evidence of benefits (Table 3) [27,34,35].

In addition to academic and professional support, the user feedback subtheme captured user interest in whether DMHTs were beneficial for peers or recommended by other trusted individuals. Patients with depression reported that other users’ experiences influenced their app use, with one user wanting to know “...if other people had success using it” [27]. Quantitatively, user ratings and user reviews were ranked as “important” or “very important” factors in DMHT use by 59.4% and 58.7% of the general population participants, respectively [35].

### Quality Assessment

The risk of bias was overall moderate. Of the 14 studies including quantitative components, only 1 (7%) used relevant validated outcome measurement instruments [33]; all others used custom questionnaires. Of the 18 studies with qualitative components, 4 (22%) were at risk of selection bias due to participants being exclusively recruited using web-based postings and research registries [33-35,37], and only 1 (6%) considered the relationship between researcher and participant when interpreting the results [36]. Full quality assessments for qualitative and quantitative study components can be found in Tables S11 and S12 in Multimedia Appendix 1, respectively.

## Discussion

### Principal Findings

This SLR aimed to identify and synthesize qualitative and quantitative evidence on how DMHTs are valued by users, payers, and employers in the United States. Evidence from users with or without diagnosed relevant disorders, caregivers, and HCPs was captured across a wide range of demographics. No study reported evaluating an app from a payer or employer perspective. Furthermore, all but one included study focused on mobile apps.

No relevant appraisals of DMHTs were identified from the FDA website searches; however, 8 relevant FDA approval labels or notifications for MH apps or guidance documents for industry and FDA staff were identified. The content of these materials overlapped with some valuation factors identified in this SLR, including evidence of clinical efficacy and safety, app maintenance, and privacy and security.

Engagement style, although not covered by the FDA materials, was the most reported theme by the studies included in this SLR and was found to overlap heavily with other themes. Engagement may be a key consideration for app developers, as app user retention can be low: 1 study showed that >90% of users had abandoned free MH apps within 30 days of installation [41]. Engagement is also a key clinical concern in terms of DMHT efficacy; one meta-analysis of 25 studies showed that increased use of DMHT modules was significantly associated with positive outcomes regardless of the target MH condition [42]. The findings of this SLR may therefore be informative to both DMHT designers and HCPs who integrate DMHTs into clinical care by providing insight on DMHT valuation and thus how use and benefit can be improved. For instance, users valued DMHTs that were easy to use and aligned with their needs and priorities, particularly through features that supported their therapeutic goals. In addition, content presented through multiple delivery modes, such as both text and visuals, promoted engagement as well as accessibility.

However, engagement and feature preference varied across populations. For instance, DMHT valuation was affected by technical literacy, which may relate to user demographics; in this SLR, veterans repeatedly emphasized technical literacy as a barrier to DMHT use [25]. Similarly, offline functionality may be more important for some users. Although 85% of the total United States population owns smartphones, only 59% of Medicare beneficiaries have access to a smartphone with a wireless plan. Moreover, beneficiaries who are older, less educated, disabled, or Black or Hispanic have even lower digital access [43,44]. These findings emphasize the importance of customizability and suggest that app development and selection in the clinical setting should consider the demographics of the target population, particularly in relation to ease of use and offline functionality.

Background and accessibility findings also identified up-front and hidden costs as barriers to DMHT use, with the willingness to pay varying among individuals. This has important implications for app development, considering that many MH apps currently on the market are direct-to-consumer sales and require out-of-pocket payment. App developers often take this approach as it does not require the accumulation of formal evidence of clinical benefit for FDA approval [45], but it may present a financial barrier to use for consumers.

Privacy and security, reported by 13 (50%) out of 26 studies, was a prevalent theme, with users primarily concerned with data and PHI security within apps. This finding reflects wider research; a 2019 review of 116 depression-related apps retrieved from iTunes and Google Play stores in 2017 found that only 4% of the identified apps had acceptable transparency in privacy and security, with many completely lacking a privacy policy [46]. Similarly, 39% of MH apps recommended by college counseling centers had no privacy policy, and of those with a policy, 88% collected user data, and 49% shared that data with third parties [4]. Most evidence identified in this SLR under this theme, as well as findings previously published in the wider literature, focuses on these remote privacy risks. However, local privacy concerns are also important to users. In particular, inconspicuous naming and the ability to hide sensitive modules within MH apps were rated as highly important by both patients and HCPs to maintain user privacy. Users emphasized a desire to avoid the stigma associated with mental illness, which was also reflected by the findings in the engagement style theme: more young adults with psychosis were more interested in in-app messaging with other patients in psychosis recovery (67.1%) than a provider and family member together (47.3%) or their personal support network (59.8%) [22]. Similarly, youths were interested in apps that could be used discreetly in school or other public settings to avoid potential MH stigma. This is a key, novel finding of this SLR, considering that many app or DMHT components on the market are named after their target disorder.

The use of DMHTs to achieve therapeutic goals was discussed from patient, caregiver, and HCP perspectives, all of which valued DMHTs that had evidence of efficacy, presented clinically actionable information, and facilitated patient-clinician relationships. Of the 5 studies that explored how HCPs value DMHTs in clinical practice, 2 (40%) were restricted to the oncology or ASD settings and were not readily generalizable to wider MH settings [20,28]. In other studies, providers reported interest in using DMHTs to facilitate asynchronous communication with patients and their caregivers, promote patient skill practice, and improve care for children through the use of games and visuals [26,30]. However, while HCPs overall believed that DMHTs improved care, some believed that their clinical training allowed for care personalization beyond what DMHTs could provide. Feature customizability and receipt of input from HCPs and users during app development and testing may be a way to mitigate these concerns, as well as concerns about safety and efficacy, as many available apps do not appropriately address user health concerns [47].

Findings additionally suggested that training and resources on DMHTs would be beneficial to ensure that HCPs were equipped to integrate DMHTs into their practices [5]. Collaboration between DMHT specialists and HCPs, along with a shift from randomized controlled trials to effectiveness-implementation hybrid trials, may be a way to streamline the integration of DMHTs into clinical care and provide more training and resources for HCPs [30,48].

### Strengths

This review followed a prespecified protocol and used systematic methods in line with the York Centre for Reviews and Dissemination guidelines [49] to conduct an exhaustive search of the literature, identifying evidence relevant to the review objectives from multiple databases and supplementary sources. The framework synthesis approach allowed for the inclusion and analysis of both qualitative and quantitative data, providing a detailed picture of not only what DMHT features users value but *why* they value them, especially in areas where valuation varies across patient demographics. In addition, the APA framework is a robust model created with patient and HCP input that incorporates key valuation themes broadly shared by other frameworks and widely acknowledged in the literature [11-13].

### Limitations

Methodological limitations should be considered when interpreting the findings of this SLR. Only publications in English and in United States populations were included. As perceptions of value are influenced by factors including cultures, laws, and health care settings, the findings of this SLR should not be generalized to other countries. For instance, trust in HCPs and rates of longstanding relationships between patients and primary care providers are lower in the United States than in many European nations [50,51], which could impact the type of support users want from DMHTs (ie, engagement style) or interest in DMHT integration with therapeutic goals.

In addition to the prespecified eligibility criteria, deprioritization strategies were implemented due to the large volume of the identified evidence, and this may have resulted in missing relevant articles. In particular, the deprioritization of secondary research and opinion pieces likely led to the exclusion of relevant discussion around payer perspectives and reimbursement, for which no evidence was included in this SLR. Furthermore, although unlikely, there may have been reporting biases in the included studies due to missing results, which this SLR was not able to assess.

This SLR identified no evidence for 3 subthemes included in the APA framework: business model (background and accessibility), which covers DMHT funding sources and potential sources of conflict, medical claims (background and accessibility), which examines whether DMHTs claim to be medical and the trustworthiness of their creators, and data ownership, access, and export (therapeutic goal), which includes sharing data with eHealth records or wellness devices (eg, Apple HealthKit [Apple Inc], Fitbit [Google LLC]). The valuation of these subthemes should be evaluated in future research.

### Conclusions

In summary, app usability, cost, accessibility and other technical considerations, and alignment with therapeutic goals were the most reported valuation factors identified by this SLR. Many studies also reported user preference for apps that incorporated privacy and security features that provided protection from stigma. However, individual DMHTs and their features are valued differently across individuals based on demographics and personal preferences. MH apps should be developed and selected with these specific user needs in mind. Feature customizability and input from users and HCPs during development may improve app usability and clinical benefit.

## Appendix

Multimedia Appendix 1 Electronic database and supplementary search terms, systematic literature review eligibility criteria, publications excluded or deprioritized at full-text review, quality assessments of included studies, and the PRISMA (Preferred Reporting Items for Systematic Reviews and Meta-Analyses) flow diagram of the identified publications.

Multimedia Appendix 2 PRISMA checklist.

## Abbreviations

ADHD: attention-deficit/hyperactivity disorder
APA: American Psychiatric Association
ASD: autism spectrum disorder
BD: bipolar disorder
DMHT: digital mental health technology
FDA: Food and Drug Administration
HCP: health care provider
MDD: major depressive disorder
MH: mental health
PHI: personal health information
PRISMA: Preferred Reporting Items for Systematic Reviews and Meta-Analyses
SLR: systematic literature review
SPIDER: Sample, Phenomenon of Interest, Design, Evaluation, Research type
