# Supplementary material for: Evaluation of Digital Mental Health Technologies in the United States: Systematic Literature Review and Framework Synthesis
Source: JMIR Ment Health. 2024 Aug 30;11:e57401. doi: 10.2196/57401 (PMC11399741; doi:10.2196/57401)
Supplement: Multimedia Appendix 1 [file mental_v11i1e57401_app1.docx]

Evaluation of Digital Mental Health Technologies in the United States: Systematic Literature Review and Framework Synthesis

Supplementary Material Table of Contents

[Supplementary Table 1. Search terms for MEDLINE (searched via Ovid SP) 2](#_Toc138681690)

[Supplementary Table 2. Search terms for Embase (searched via Ovid SP) 4](#_Toc138681691)

[Supplementary Table 3. Search terms for the PsycINFO (searched via APA PsycNET) 6](#_Toc138681692)

[Supplementary Table 4. Search terms for the Cochrane Library (searched simultaneously via the Wiley platform) 8](#_Toc138681693)

[Supplementary Table 5. Search terms for use in the HTAD (searched using the INAHTA platform) 10](#_Toc138681694)

[Supplementary Table 6. Search terms used for congress websites 12](#_Toc138681695)

[Supplementary Table 7. Search terms for hand searches of the FDA website 15](#_Toc138681696)

[Supplementary Table 8. Eligibility criteria for the SLR 16](#_Toc138681697)

[Supplementary Table 9. Publications excluded at the full text review stage in the SLR 18](#_Toc138681698)

[Supplementary Table 10. Publications deprioritized at the full text review stage in the SLR 34](#_Toc138681699)

[Supplementary Table 11. Quality assessment of quantitative components of included studies using the CASP cohort study appraisal checklist 41](#_Toc138681700)

[Supplementary Table 12. Quality assessment of qualitative components of included studies using the CASP qualitative appraisal checklist 43](#_Toc138681701)

[Supplementary Figure 1. PRISMA flow diagram for the publications identified in the SLR](#_Toc138681701) 45

Supplementary Table 1. Search terms for MEDLINE (searched via Ovid SP)

| **Term group** | **#** | **Searches** | **Results**  **17^th^ June 2022** |
| --- | --- | --- | --- |
| Assessment | 1 | *Technology assessment, biomedical/ or *checklist/ or *consensus/ or *consumer behavior/ or *patient acceptance of health care/ or *consumer satisfaction/ or *patient preference/ or *decision making/ | 107101 |
|  | 2 | (assessment or checklist or framework or consensus or valu* or apprais* or evaluat* or cost* or rating* or criteria* or regulation or preference* or acceptance* or satisf* or perspective* or consum* or payer* or employer*).ti,ab,kf. | 9397037 |
|  | 3 | (Willingness to pay or WTP).ti,ab,kf. | 7981 |
|  | 4 | or/1-3 | 9444836 |
| Digital Health | 5 | *mobile applications/ | 8197 |
|  | 6 | (mobile health or mhealth or m-health or ehealth or e-health or app or apps or (digital adj3 (health* or medicine or intervention or platform or therap*)) or ((mobile or cell phone or smartphone or smart phone or android or iphone or web*) adj2 application*)).ti,ab,kf. | 68456 |
|  | 7 | or/5-6 | 69693 |
| CNS and Mental Health | 8 | exp psychotic disorders/ or exp "schizophrenia spectrum and other psychotic disorders"/ | 158724 |
|  | 9 | (schizo$ or psychotic or psychosis).ti,ab,kf. | 197514 |
|  | 10 | exp "bipolar and related disorders"/ | 43849 |
|  | 11 | ((bipolar adj3 disorder$) or depress$).ti,ab,kf. | 548914 |
|  | 12 | (mania$ or manic$).ti,ab,kf. | 21457 |
|  | 13 | exp Depressive Disorder/ or exp Depression/ | 245250 |
|  | 14 | ((major or clinical or melancholic or catatonic or atypical or unipolar or disorder$ or episod$) adj2 depress$).ti,ab,kf. | 88004 |
|  | 15 | (unipolar disorder or MDD).ti,ab,kf. | 16762 |
|  | 16 | Stress Disorders, Post-Traumatic/ | 38745 |
|  | 17 | (PTSD or ((posttraumatic or post-traumatic) adj stress)).ti,ab,kf. | 45630 |
|  | 18 | exp Mood Disorders/ or exp personality disorders/ or borderline personality disorder/ | 171820 |
|  | 19 | exp anxiety disorders/ | 87016 |
|  | 20 | (AXIS-I or AXIS-1 or mood disorder$ or personality disorder$ or borderline personality or BPD or anxiety disorder$ or obsessive compulsive$ or OCD or GAD).ti,ab,kf. | 110239 |
|  | 21 | Alzheimer disease/ | 110462 |
|  | 22 | (Alzheimer* or Alzheimer disease*).ti,ab,kf. | 173543 |
|  | 23 | exp epilepsy/ | 121811 |
|  | 24 | epilep*.kw. | 28107 |
|  | 25 | Attention Deficit Disorder with Hyperactivity/ | 33020 |
|  | 26 | (attention deficit disorder or attention deficit hyperactive disorder or ADHD).ti,ab,kf. | 31061 |
|  | 27 | Autism spectrum disorder/ or autistic disorder/ or child development disorders, pervasive/ or asperger syndrome/ | 43987 |
|  | 28 | (autis* or pervasive development* disorder* or asperger* syndrome).ti,ab,kf. | 61306 |
|  | 29 | exp mental health/ or stress, psychological/ | 179972 |
|  | 30 | (mental health* or well being* or wellbeing or stress*).ti,ab,kf. | 1260478 |
|  | 31 | or/8-30 | 2416147 |
| US | 32 | exp United States/ | 1435578 |
|  | 33 | (USA or United States or America$ or New York or Los Angeles or Chicago or Houston or Phoenix or Philadelphia or San Diego or Austin or Jacksonville or San Francisco or Columbus or Fort Worth or Indianapolis or Charlotte or Seattle or Denver or Washington or Boston or El Paso or Detroit or Nashville or Memphis or Portland or Oklahoma or Las Vegas or Louisville or California or Texas or Florida or Pennsylvania or Illinois or Ohio or Georgia or Carolina or Michigan or New Jersey or Virginia or Washington or Arizona or Massachusetts or Tennessee or Indiana or Missouri or Maryland or Wisconsin or Colorado or Minnesota or Alabama or Louisiana or Kentucky or Oregon or Oklahoma or Connecticut or Utah or Iowa or Nevada or Arkansas or Mississippi or Kansas or Nebraska or Idaho or Hawaii or New Hampshire or Maine or Montana or Rhode Island or Delaware or Dakota or Alaska or District of Columbia or Vermont or Wyoming).ti,ab,jw,in. | 8906505 |
|  | 34 | or/32-33 | 9360449 |
|  | 35 | exp africa/ or exp antarctic regions/ or exp arctic regions/ or exp asia/ or exp europe/ or exp oceania/ | 2892322 |
|  | 36 | 34 not 35 | 8995128 |
| Standard Exclusion Filter | 37 | exp animals/ not exp humans/ | 5018578 |
|  | 38 | (comment or editorial or case reports or historical article).pt. | 3990659 |
|  | 39 | (case stud* or case report*).ti. | 348790 |
|  | 40 | or/37-39 | 8998760 |
| Combination | 41 | 4 and 7 and 31 and 36 | 3382 |
|  | 42 | 41 not 40 | 3006 |
|  | 43 | limit 42 to yr=2017-current | 2058 |

**Databases:** Ovid MEDLINE(R) and Epub Ahead of Print, In-Process, In-Data-Review & Other Non-Indexed Citations and Daily 1946 to June 16, 2022.
**Abbreviations:** CNS, central nervous system; US, United States.

Supplementary Table 2. Search terms for Embase (searched via Ovid SP)

| **Term group** | **#** | **Searches** | **Results**  **17^th^ June 2022** |
| --- | --- | --- | --- |
| Assessment | 1 | *Biomedical technology assessment/ or *checklist/ or *consensus/ or *consumer attitude/ or *patient attitude/ or *consumer satisfaction/ or *patient preference/ or *decision making/ | 117049 |
|  | 2 | (assessment or checklist or framework or consensus or valu* or apprais* or evaluat* or cost* or rating* or criteria* or regulation or preference* or acceptance* or satisf* or perspective* or consum* or payer* or employer*).ti,ab,kf. | 12382852 |
|  | 3 | Willingness to pay/ | 1844 |
|  | 4 | (Willingness to pay or WTP).ti,ab,kf. | 12065 |
|  | 5 | or/1-4 | 12435059 |
| Digital Health | 6 | *mobile application/ | 9012 |
|  | 7 | ((digital adj3 (health* or medicine or intervention or platform or therap*)) or mobile health or mhealth or m-health or ehealth or e-health or app or apps or ((mobile or cell phone or smartphone or smart phone or android or iphone or web*) adj2 application*)).ti,ab,kf. | 87886 |
|  | 8 | or/6-7 | 89183 |
| CNS and Mental Health | 9 | exp psychosis/ or exp schizophrenia spectrum disorder/ | 304821 |
|  | 10 | (schizo$ or psychotic or psychosis).ti,ab,kf. | 257601 |
|  | 11 | exp bipolar disorder/ | 71457 |
|  | 12 | ((bipolar adj3 disorder$) or depress$).ti,ab,kf. | 736463 |
|  | 13 | (mania$ or manic$).ti,ab,kf. | 28674 |
|  | 14 | exp depression/ | 550673 |
|  | 15 | ((major or clinical or melancholic or catatonic or atypical or unipolar or disorder$ or episod$) adj2 depress$).ti,ab,kf. | 121947 |
|  | 16 | (unipolar disorder or MDD).ti,ab,kf. | 24789 |
|  | 17 | posttraumatic stress disorder/ | 71784 |
|  | 18 | (PTSD or ((posttraumatic or post-traumatic) adj stress)).ti,ab,kf. | 58403 |
|  | 19 | exp mood disorder/ or exp personality disorder/ or borderline state/ | 640925 |
|  | 20 | exp anxiety disorder/ | 285172 |
|  | 21 | (AXIS-I or AXIS-1 or mood disorder$ or personality disorder$ or borderline personality or BPD or anxiety disorder$ or obsessive compulsive$ or OCD or GAD).ti,ab,kf. | 156302 |
|  | 22 | Alzheimer disease/ | 226887 |
|  | 23 | (Alzheimer* or Alzheimer disease*).ti,ab,kf. | 238575 |
|  | 24 | exp epilepsy/ | 258510 |
|  | 25 | epilep*.ti,ab,kf. | 220234 |
|  | 26 | attention deficit disorder/ | 67689 |
|  | 27 | (attention deficit disorder or attention deficit hyperactive disorder or ADHD).ti,ab,kf. | 44756 |
|  | 28 | autism/ or asperger syndrome/ | 79461 |
|  | 29 | (autis* or pervasive development* disorder* or asperger* syndrome).ti,ab,kf. | 79189 |
|  | 30 | exp mental health/ or wellbeing/ or mental stress/ | 349717 |
|  | 31 | (mental health* or well being* or wellbeing or stress*).ti,ab,kf. | 1575534 |
|  | 32 | or/9-31 | 3352627 |
| US | 33 | exp United States/ | 1312092 |
|  | 34 | (USA or United States or America$ or New York or Los Angeles or Chicago or Houston or Phoenix or Philadelphia or San Diego or Austin or Jacksonville or San Francisco or Columbus or Fort Worth or Indianapolis or Charlotte or Seattle or Denver or Washington or Boston or El Paso or Detroit or Nashville or Memphis or Portland or Oklahoma or Las Vegas or Louisville or California or Texas or Florida or Pennsylvania or Illinois or Ohio or Georgia or Carolina or Michigan or New Jersey or Virginia or Washington or Arizona or Massachusetts or Tennessee or Indiana or Missouri or Maryland or Wisconsin or Colorado or Minnesota or Alabama or Louisiana or Kentucky or Oregon or Oklahoma or Connecticut or Utah or Iowa or Nevada or Arkansas or Mississippi or Kansas or Nebraska or Idaho or Hawaii or New Hampshire or Maine or Montana or Rhode Island or Delaware or Dakota or Alaska or District of Columbia or Vermont or Wyoming).ti,ab,kf,in,ad. | 11685483 |
|  | 35 | or/33-34 | 12119632 |
|  | 36 | exp africa/ or exp antarctica/ or exp arctic/ or exp asia/ or exp europe/ or exp "arctic and antarctic"/ or exp oceanic regions/ | 3258536 |
|  | 37 | 35 not 36 | 11599567 |
| Standard Exclusion Filter | 38 | ("conference abstract" or "conference review").pt. | 4441068 |
|  | 39 | limit 38 to yr="1974-2018" | 3401805 |
|  | 40 | exp animals/ not exp humans/ | 4967131 |
|  | 41 | editorial.pt. | 729476 |
|  | 42 | editorial/ or case report/ | 3447094 |
|  | 43 | (case stud$ or case report$).ti. | 425207 |
|  | 44 | or/39-43 | 11430163 |
| Combination | 45 | 5 and 8 and 32 and 37 | 4669 |
|  | 46 | 45 not 44 | 3010 |
|  | 47 | limit 46 to yr=2017-current | 2077 |

**Database:** Embase 1974 to 2022 June 16.
**Abbreviations:** CNS, central nervous system; US, United States.

Supplementary Table 3. Search terms for the PsycINFO (searched via APA PsycNET)

| **Term group** | **#** | **Search logic** | **Terms** | **Search in field** | **Results**  **17^th^ June 2022** |
| --- | --- | --- | --- | --- | --- |
| Digital Health | 1 | - | mobile applications | MeSH | - |
|  | 2 | - | ((digital NEAR/3 (health* OR medicine OR intervention OR platform OR therap*)) OR “mobile health” OR mhealth OR m-health OR ehealth OR e-health OR app OR apps OR ((mobile OR “cell phone” OR smartphone OR “smart phone” OR android OR iphone OR web*) NEAR/2 application*)) | Abstract | - |
|  | 3 | - | ((digital NEAR/3 (health* OR medicine OR intervention OR platform OR therap*)) OR “mobile health” OR mhealth OR m-health OR ehealth OR e-health OR app OR apps OR ((mobile OR “cell phone” OR smartphone OR “smart phone” OR android OR iphone OR web*) NEAR/2 application*)) | Keywords | - |
|  | 4 | OR | Combined Total | - | 22313 |
| CNS and Mental Health | 5 | - | “psychotic disorders” OR "schizophrenia spectrum and other psychotic disorders" | MeSH | - |
|  | 6 | - | schizo* OR psychotic OR psychosis | Abstract | - |
|  | 7 | - | schizo* OR psychotic OR psychosis | Keywords | - |
|  | 8 | - | "bipolar and related disorders" | MeSH | - |
|  | 9 |  | (bipolar NEAR/3 disorder*) OR depress* | Abstract | - |
|  | 10 | - | (bipolar NEAR/3 disorder*) OR depress* | Keywords | - |
|  | 11 |  | mania* OR manic* | Abstract | - |
|  | 12 | - | mania* OR manic* | Keywords | - |
|  | 13 | - | “depressive disorder” OR depression | MeSH | - |
|  | 14 | - | (major OR clinical OR melancholic OR catatonic OR atypical OR unipolar OR disorder* OR episode*) NEAR/2 depress* | Abstract | - |
|  | 15 | - | (major OR clinical OR melancholic OR catatonic OR atypical OR unipolar OR disorder* OR episode*) NEAR/2 depress* | Keywords | - |
|  | 16 |  | “unipolar disorder” OR MDD | Abstract | - |
|  | 17 | - | “unipolar disorder” OR MDD | Keywords | - |
|  | 18 | - | “Stress Disorders, Post-Traumatic” | MeSH | - |
|  | 19 | - | PTSD OR ((posttraumatic OR post-traumatic) NEAR/3 stress) | Abstract | - |
|  | 20 | - | PTSD OR ((posttraumatic OR post-traumatic) NEAR/3 stress) | Keywords | - |
|  | 21 | - | “mood disorders” OR “personality disorders” OR “borderline personality disorder” | MeSH | - |
|  | 22 | - | "anxiety disorder” | MeSH | - |
|  | 23 |  | “AXIS-I” OR “AXIS-1” OR “mood disorder*” OR “personality disorder*” OR “borderline personality” OR BPD OR “anxiety disorder*” OR “obsessive compulsive*” OR OCD OR GAD | Abstract | - |
|  | 24 | - | “AXIS-I” OR “AXIS-1” OR “mood disorder*” OR “personality disorder*” OR “borderline personality” OR BPD OR “anxiety disorder*” OR “obsessive compulsive*” OR OCD OR GAD | Keywords | - |
|  | 25 | - | “Alzheimer disease” | MeSH | - |
|  | 26 |  | Alzheimer* OR “Alzheimer disease*” | Abstract | - |
|  | 27 | - | Alzheimer* OR “Alzheimer disease*” | Keywords | - |
|  | 28 | - | Epilepsy | MeSH | - |
|  | 29 | - | epilep* | Abstract | - |
|  | 30 | - | epilep* | Keywords | - |
|  | 31 | - | “attention deficit disorder with hyperactivity" | MeSH | - |
|  | 32 | - | “attention deficit disorder” OR “attention deficit hyperactive disorder” OR ADHD | Abstract | - |
|  | 33 | - | “attention deficit disorder” OR “attention deficit hyperactive disorder” OR ADHD | Keywords | - |
|  | 34 | - | “Autism spectrum disorder” or “autistic disorder” or “child development disorders, pervasive” or “asperger syndrome” | MeSH | - |
|  | 35 | - | autis* or “pervasive development* disorder*” or “asperger* syndrome” | Abstract | - |
|  | 36 | - | autis* or “pervasive development* disorder*” or “asperger* syndrome” | Keywords | - |
|  | 37 | - | “Mental health” OR “stress, psychological” | MeSH | - |
|  | 38 | - | “mental health*” OR “well being*” OR stress* | Abstract | - |
|  | 39 | - | “mental health*” OR “well being*” OR stress* | Keywords | - |
|  | 40 | OR | Combined Total | - | 250413 |
| Combination | 41 | AND | Combined Total | - | 282 |

**Abbreviations:** CNS, central nervous system.

Supplementary Table 4. Search terms for the Cochrane Library (searched simultaneously via the Wiley platform)

| **Term group** | **#** | **Searches** | **Results**  **17^th^ June 2022** |
| --- | --- | --- | --- |
| Assessment | 1 | [mh ^"Technology assessment, biomedical"] or [mh ^checklist] or [mh ^consensus] or [mh ^"consumer behavior"] or [mh ^"patient acceptance of health care"] or [mh ^"patient preference"] or [mh ^"decision making"] | 7612 |
|  | 2 | (assessment or checklist or framework or consensus or valu* or apprais* or evaluat* or cost* or rating* or criteria* or regulation or preference* or acceptance* or satisf* or perspective* or consum* or payer* or employer*):ti,ab,kw | 1056876 |
|  | 3 | ("Willingness to pay" or WTP):ti,ab,kw | 1686 |
|  | 4 | {OR #1-#3} | 1057596 |
| Digital Health | 5 | [mh ^"mobile applications"] | 1070 |
|  | 6 | ((digital near/3 (health* or medicine or intervention or platform or therap*)) or "mobile health" or mhealth or m-health or ehealth or e-health or app or apps or ((mobile or "cell phone" or smartphone or "smart phone" or android or iphone or web*) near/2 application*)):ti,ab,kw | 13116 |
|  | 7 | #5 or #6 | 13116 |
| CNS and Mental Health | 8 | [mh "psychotic disorders"] or [mh "schizophrenia spectrum and other psychotic disorders"] | 9802 |
|  | 9 | (schizo* or psychotic or psychosis):ti,ab,kw | 24799 |
|  | 10 | [mh "bipolar and related disorders"] | 2874 |
|  | 11 | ((bipolar near/3 disorder*) or depress*):ti,ab,kw | 99100 |
|  | 12 | (mania* or manic*):ti,ab,kw | 3447 |
|  | 13 | [mh "depressive disorder"] or [mh "depression"] | 23248 |
|  | 14 | ((major or clinical or melancholic or catatonic or atypical or unipolar or disorder* or episod*) near/2 depress*):ti,ab,kw | 26005 |
|  | 15 | ("unipolar disorder" or MDD):ti,ab,kw | 4108 |
|  | 16 | [mh ^"Stress Disorders, Post-Traumatic"] | 3099 |
|  | 17 | (PTSD or (("posttraumatic or post-traumatic") next stress)):ti,ab,kw | 5033 |
|  | 18 | [mh "mood disorders"] or [mh "personality disorders"] or [mh "anxiety disorders"] | 21500 |
|  | 19 | (AXIS-I or "AXIS I" or AXIS-1 or "AXIS 1" or (mood NEXT disorder*) or (personality NEXT disorder*) or "borderline personality" or BPD or (anxiety NEXT disorder*) or (obsessive NEXT compulsive*) or OCD or GAD):ti,ab,kw | 22816 |
|  | 20 | [mh ^"Alzheimer disease"] | 3752 |
|  | 21 | (Alzheimer* or (Alzheimer disease*)):ti,ab,kw | 12399 |
|  | 22 | [mh "epilepsy"] | 2584 |
|  | 23 | epilep*:ti,ab,kw | 8824 |
|  | 24 | [mh ^"attention deficit disorder with hyperactivity"] | 3030 |
|  | 25 | ("attention deficit disorder" or "attention deficit hyperactive disorder" or ADHD):ti,ab,kw | 6322 |
|  | 26 | [mh ^"Autism spectrum disorder"] or [mh ^"child development disorders, pervasive"] or [mh ^"asperger syndrome"] or [mh ^"autistic disorder"] | 2021 |
|  | 27 | (autis* or (pervasive NEXT development* NEXT disorder*) or (asperger* NEXT syndrome)):ti,ab,kw | 4410 |
|  | 28 | [mh "Mental health"] or [mh ^"stress, psychological"] | 8012 |
|  | 29 | ((mental NEXT health*) or (well NEXT being*) or wellbeing or stress*):ti,ab,kw | 102355 |
|  | 30 | {OR #8-#29} | 231367 |
| US | 31 | [mh "United States"] | 20631 |
|  | 32 | (USA or "United States" or America* or "New York" or "Los Angeles" or Chicago or Houston or Phoenix or Philadelphia or "San Diego" or Austin or Jacksonville or "San Francisco" or Columbus or "Fort Worth" or Indianapolis or Charlotte or Seattle or Denver or Washington or Boston or "El Paso" or Detroit or Nashville or Memphis or Portland or Oklahoma or "Las Vegas" or Louisville or California or Texas or Florida or Pennsylvania or Illinois or Ohio or Georgia or Carolina or Michigan or "New Jersey" or Virginia or Washington or Arizona or Massachusetts or Tennessee or Indiana or Missouri or Maryland or Wisconsin or Colorado or Minnesota or Alabama or Louisiana or Kentucky or Oregon or Connecticut or Utah or Iowa or Nevada or Arkansas or Mississippi or Kansas or Nebraska or Idaho or Hawaii or "New Hampshire" or Maine or Montana or "Rhode Island" or Delaware or Dakota or Alaska or "District of Columbia" or Vermont or Wyoming) | 431124 |
|  | 33 | #31 or #32 | 431223 |
|  | 34 | [mh Africa] or [mh "antarctic regions"] or [mh "arctic regions"] or [mh asia] or [mh Europe] or [mh oceania] | 66466 |
|  | 35 | #33 not #34 | 422535 |
| Combination | 36 | #4 and #7 and #30 and #35 | 663 |
|  | 37 | #36 with Cochrane Library publication date Between Jan 2017 and Jul 2022, in Cochrane Reviews, Cochrane Protocols | 17 |
|  | 38 | #36 with Publication Year from 2017 to 2022, in Trials | 511 |

**Databases:** CDSR Issue 6 of 12, June 2022; CENTRAL Issue 5 of 12, May 2022.
**Abbreviations:** CNS, central nervous system; US, United States.

Supplementary Table 5. Search terms for use in the HTAD (searched using the INAHTA platform)

| **Term group** | **#** | **Searches** | **Results**  **17^th^ June 2022** |
| --- | --- | --- | --- |
| Assessment | 1 | "Technology assessment, biomedical"[mh] or checklist[mh] or consensus[mh] or "consumer behavior"[mh] or "patient acceptance of health care"[mh] or "consumer satisfaction"[mh] or "patient preference"[mh] or "decision making"[mh] | 480 |
|  | 2 | (assessment or checklist or framework or consensus or valu* or apprais* or evaluat* or cost* or rating* or criteria* or regulation or preference* or acceptance* or satisf* or perspective* or consum* or payer* or employer*) | 11415 |
|  | 3 | "Willingness to pay" or WTP | 84 |
|  | 4 | #1 or #2 or #3 | 11470 |
| Digital Health | 5 | "mobile applications"[mh] | 4 |
|  | 6 | (digital AND (health* or medicine or intervention or platform or therap*)) or "mobile health" or mhealth or m-health or ehealth or e-health or app or apps or ((mobile or cell phone or smartphone or "smart phone" or android or iphone or web*) AND application*) | 12257 |
|  | 7 | #5 or #6 | 12257 |
| CNS and Mental Health | 8 | "psychotic disorders"[mhe] or "schizophrenia spectrum and other psychotic disorders"[mhe] | 106 |
|  | 9 | (schizo* or psychotic or psychosis) | 136 |
|  | 10 | "bipolar and related disorders"[mhe] | 33 |
|  | 11 | ((bipolar AND disorder*) or depress*) | 437 |
|  | 12 | (mania* or manic*) | 22 |
|  | 13 | "depressive disorder"[mhe] or depression[mhe] | 208 |
|  | 14 | ((major or clinical or melancholic or catatonic or atypical or unipolar or disorder* or episode*) AND depress*) | 267 |
|  | 15 | (unipolar disorder or MDD) | 9 |
|  | 16 | "Stress Disorders, Post-Traumatic"[mh] | 35 |
|  | 17 | (PTSD or ((posttraumatic or post-traumatic) AND stress)) | 53 |
|  | 18 | "mood disorders"[mhe] or "personality disorders"[mhe] or "borderline personality disorder"[mh] | 164 |
|  | 19 | "anxiety disorders"[mhe] | 55 |
|  | 20 | (AXIS-I or AXIS-1 or "AXIS I" or "AXIS 1" or "mood disorder*" or "personality disorder*" or "borderline personality" or BPD or "anxiety disorder*" or "obsessive compulsive*" or OCD or GAD) | 72 |
|  | 21 | "Alzheimer disease"[mh] | 101 |
|  | 22 | (Alzheimer* or "Alzheimer disease*") | 117 |
|  | 23 | Epilepsy[mhe] | 85 |
|  | 24 | epilep* | 140 |
|  | 25 | "attention deficit disorder with hyperactivity"[mh] | 62 |
|  | 26 | ("attention deficit disorder" or "attention deficit hyperactive disorder" or ADHD) | 64 |
|  | 27 | "Autism spectrum disorder"[mh] or "autistic disorder"[mh] or "child development disorders, pervasive"[mh] or "asperger syndrome"[mh] | 78 |
|  | 28 | (autis* or "pervasive development* disorder*" or "asperger* syndrome") | 90 |
|  | 29 | "Mental health"[mhe] or "stress, psychological"[mh] | 181 |
|  | 30 | ("mental health*" or wellbeing or "well being*" or stress*) | 535 |
|  | 31 | #8 or #9 or #10 or #11 or #12 or #13 or #14 or #15 or #16 or #17 or #18 or #19 or #20 or #21 or #22 or #23 or #24 or #25 or #26 or #27 or #28 or #29 or #30 | 1478 |
| US | 32 | "United States"[mhe] | 55 |
|  | 33 | (USA or "United States" or America* or "New York" or "Los Angeles" or Chicago or Houston or Phoenix or Philadelphia or "San Diego" or Austin or Jacksonville or "San Francisco" or Columbus or "Fort Worth" or Indianapolis or Charlotte or Seattle or Denver or Washington or Boston or "El Paso" or Detroit or Nashville or Memphis or Portland or Oklahoma or "Las Vegas" or Louisville or California or Texas or Florida or Pennsylvania or Illinois or Ohio or Georgia or Carolina or Michigan or "New Jersey" or Virginia or Washington or Arizona or Massachusetts or Tennessee or Indiana or Missouri or Maryland or Wisconsin or Colorado or Minnesota or Alabama or Louisiana or Kentucky or Oregon or Oklahoma or Connecticut or Utah or Iowa or Nevada or Arkansas or Mississippi or Kansas or Nebraska or Idaho or Hawaii or "New Hampshire" or Maine or Montana or "Rhode Island" or Delaware or Dakota or Alaska or "District of Columbia" or Vermont or Wyoming) | 4498 |
|  | 34 | #32 or #33 | 4508 |
| Combination | 35 | #4 and #7 and #31 and #34 | 109 |
|  | 36 | #35 filter: year 2017-2022 | 29 |

**Database:** CNS, central nervous system; HTAD, Health Technology Assessment Database; INAHTA, International Network of Agencies for Health Technology Assessment; US, United States.

Supplementary Table 6. Search terms used for congress websites

| **Conference** | **Link** | **Search strategy** | **Results** | **Number included** | **Number included following deprior-itization** |
| --- | --- | --- | --- | --- | --- |
| HTAi 2021 | [International Journal of Technology Assessment in Health Care: Volume 37 - Innovation through HTA \| Cambridge Core](https://www.cambridge.org/core/journals/international-journal-of-technology-assessment-in-health-care/issue/innovation-through-hta/B3DCFCC483ECA7B62B82BB8F4697654F) | Titles were screened, with further reviewing based on relevant titles | 95 | 0 | 0 |
| HTAi 2020 | [International Journal of Technology Assessment in Health Care: Volume 36 - Attaining, Maintaining, and Sustaining Healthcare Systems in a Changing World: The Role of HTA \| Cambridge Core](https://www.cambridge.org/core/journals/international-journal-of-technology-assessment-in-health-care/issue/257D8FF2EF0B5DC748A9203974C04F6E?sort=canonical.position%3Aasc&pageNum=1&searchWithinIds=257D8FF2EF0B5DC748A9203974C04F6E&productType=JOURNAL_ARTICLE&template=cambridge-core%2Fjournal%2Farticle-listings%2Flistings-wrapper&hideArticleJournalMetaData=true&displayNasaAds=false) | Titles were screened, with further reviewing based on relevant titles | 113 | 0 | 0 |
| HTAi 2019 | [International Journal of Technology Assessment in Health Care: Volume 35 - HTA Beyond 2020: Ready for a New Decade? \| Cambridge Core](https://www.cambridge.org/core/journals/international-journal-of-technology-assessment-in-health-care/issue/9B9F255EC7042C57EBAB04AF08AEC1C3) | Titles were screened, with further reviewing based on relevant titles | 261 | 2 | 0 |
| SDMH Annual Meeting 2022 | [Center for Open Science (OSF)](https://osf.io/meetings/SDMH2022) | Titles were screened, with further reviewing based on relevant titles | 58 | 7 | 0 |
| APA 2022 | NA | Search terms were searched in turn using the Control + F function: digital | 29 | 0 | 0 |
|  |  | Search terms were searched in turn using the Control + F function: application | 29 | 0 |  |
|  |  | Search terms were searched in turn using the Control + F function: app (whole words only) | 2 | 0 |  |
|  |  | Search terms were searched in turn using the Control + F function: apps (whole words only) | 2 | 0 |  |
| APA 2021 | NA | Search terms were searched in turn using the Control + F function: digital | 16 | 0 | 0 |
|  |  | Search terms were searched in turn using the Control + F function: application | 36 | 0 |  |
|  |  | Search terms were searched in turn using the Control + F function: app (whole words only) | 5 | 0 |  |
|  |  | Search terms were searched in turn using the Control + F function: apps (whole words only) | 4 | 0 |  |
| APA 2019 | NA | Search terms were searched in turn using the Control + F function: digital | 28 | 0 | 0 |
|  |  | Search terms were searched in turn using the Control + F function: application | 28 | 0 |  |
|  |  | Search terms were searched in turn using the Control + F function: app (whole words only) | 37 | 0 |  |
|  |  | Search terms were searched in turn using the Control + F function: apps (whole words only) | 22 | 0 |  |
| DIA 2022 | [DIA 2022 Poster Presentations](https://www.diaglobal.org/en/flagship/dia-2022/program/about-our-offerings/posters/poster-presentations) | Search terms were searched using the search bar: digital | 0 | 0 | 0 |
|  |  | Search terms were searched using the search bar: application | 1 | 0 |  |
|  |  | Search terms were searched using the search bar: app (whole words only) | 6 | 0 |  |
|  |  | Search terms were searched using the search bar: apps (whole words only) | 0 | 0 |  |
| DIA 2021 | [DIA 2021 Poster Presentations](https://www.diaglobal.org/en/flagship/dia-2021/program/about-our-offerings/posters/poster-presentations) | Search terms were searched using the search bar: digital | 2 | 0 | 0 |
|  |  | Search terms were searched using the search bar: application | 1 | 0 |  |
|  |  | Search terms were searched using the search bar: app (whole words only) | 6 | 0 |  |
|  |  | Search terms were searched using the search bar: apps (whole words only) | 0 | 0 |  |
| DIA 2020 | [DIA 2020 Poster Presentations](https://www.diaglobal.org/en/flagship/dia-2020/program/about-our-offerings/posters/poster-presentations) | Search terms were searched using the search bar: digital | 2 | 0 | 0 |
|  |  | Search terms were searched using the search bar: application | 2 | 0 |  |
|  |  | Search terms were searched using the search bar: app (whole words only) | 8 | 0 |  |
|  |  | Search terms were searched using the search bar: apps (whole words only) | 0 | 0 |  |
| ASCP 2022 | [ASCP Meetings - ASCP - American Society of Clinical Psychopharmacology (ascpp.org)](https://ascpp.org/ascp-meetings/) | Search terms were searched using the search bar: digital | 13 | 0 | 0 |
|  |  | Search terms were searched using the search bar: application | 13 | 0 |  |
|  |  | Search terms were searched using the search bar: app (whole words only) | 3 | 0 |  |
|  |  | Search terms were searched using the search bar: apps (whole words only) | 0 | 0 |  |
| ASCP 2021 | [ASCP Meetings - ASCP - American Society of Clinical Psychopharmacology (ascpp.org)](https://ascpp.org/ascp-meetings/) | Search terms were searched using the search bar: digital | 2 | 0 | 0 |
|  |  | Search terms were searched using the search bar: application | 2 | 0 |  |
|  |  | Search terms were searched using the search bar: app (whole words only) | 8 | 0 |  |
|  |  | Search terms were searched using the search bar: apps (whole words only) | 0 | 0 |  |
| ASCP 2020 | [ASCP Meetings - ASCP - American Society of Clinical Psychopharmacology (ascpp.org)](https://ascpp.org/ascp-meetings/) | Search terms were searched using the search bar: digital | 5 | 0 | 0 |
|  |  | Search terms were searched using the search bar: application | 4 | 0 |  |
|  |  | Search terms were searched using the search bar: app (whole words only) | 1 | 0 |  |
|  |  | Search terms were searched using the search bar: apps (whole words only) | 0 | 0 |  |

**Abbreviations:** APA, American Psychiatric Association; ASCP, American Society for Clinical Psychiatry; DIA, Drug Information Association; HTAi, Health Technology Assessment International; NA, not applicable; SDMH, Society for Digital Mental Health.

Supplementary Table 7. Search terms for hand searches of the FDA website

| **Terms** |
| --- |
| mHealth |
| digital health |
| mobile health |
| mental health app |

Supplementary Table 8. Eligibility criteria for the SLR

| **Category** | **Inclusion Criteria** | **Exclusion Criteria** |
| --- | --- | --- |
| Sample | Target audience for DMHT:   - Payers - Employers - Consumers | - DMHT with target audience other than payers, employers, or consumers |
| Phenomenon of interest | - Factors considered in the evaluation of DMHT for mental health and CNS indications, to include digital therapeutics (e.g. ingestible pill monitors), mobile health (e.g. applications), and digital devices (e.g. wearables) | - Factors considered in the evaluation of telehealth solutions or telemedicine technologies - Studies only reporting app-specific outcomes^a^ - Studies only reporting efficacy outcomes for DMHT^a^ - Studies only reviewing apps against a particular framework^a^ |
| Design | Any, including:   - Interventional studies - Observational studies - Focus group reports - Vignette studies - Guidelines - Evaluation frameworks - Survey or interview studies - HTAs | - Studies not reporting any outcomes of relevance listed - Mixed-methods research - Reviews |
| Evaluation | Including, but not limited to:   - Quantitative outcomes - Outputs from relevant quantitative scales of useability and employer or consumer preference for DMHT features - Quantitative measures of effectiveness - Quantitative measures of willingness to pay - Quantitative measures of privacy and security - Qualitative outcomes - Qualitative descriptions of DMHT usability, simplicity, and reliability - Qualitative descriptions of employer or consumer preferences for DMHT features - Qualitative descriptions of privacy and security - Qualitative descriptions of DMHT accessibility - Qualitative considerations of ethical aspects | - Studies not reporting any outcomes of relevance listed |
| Research type | Any, including:   - Qualitative research - Quantitative research - Mixed-methods research | - Secondary research, including systematic and narrative reviews, guidelines, and opinion articles^a^ |
| Other considerations | - Date limits: - Journal articles (and other full publication types, such as guidelines) published from 2017 onwards - Conference abstracts from 2019 onwards - Studies based in the US - Abstract or full-text in English | - Journal articles published prior to 2017 - Abstracts from conferences conducted prior to 2019 - Non-US studies - Non-English publications - DMHT for animals |

**Footnotes:** ^a^These eligibility criteria were added during abstract or full-text review due to the large volume of evidence in order to prioritize the most relevant studies for inclusion.
**Abbreviations:** CNS, central nervous system; DMHT, digital mental health technologies; HTA, health technology assessment; SLR, systematic literature review; US, United States.

Supplementary Table 9. Publications excluded at the full text review stage in the SLR

| **#** | **Reference** | **Reason for exclusion** |
| --- | --- | --- |
| 1 | Abhulimen S, Hirsch A. Quantifying the economic impact of a digital self-care behavioral health platform on Missouri Medicaid expenditures. Journal of Medical Economics 2018;21(11):1084-1090. | Non-relevant outcome |
| 2 | Abraham J, Johns J, Hibbert S, et al. Pmh53 Collective Monitoring of Adhd Symptoms and Behaviors Via Mobile Application (Akl-X01): An Evaluation of Caregiver-Reported Usability, Value, and Engagement. Value in Health 2019;22(Supplement 2):S235. | Non-relevant outcome |
| 3 | Abrantes AM, Blevins CE, Battle C, et al. Fit&sober app: Integrating fitbit into a smartphone app to help increase physical activity during early recovery from alcohol use disorder. Alcoholism: Clinical and Experimental Research 2019;43(Supplement 1):318A. | Non-relevant digital therapeutic |
| 4 | Abrantes AM, Blevins CE, Battle CL, et al. Developing a Fitbit-supported lifestyle physical activity intervention for depressed alcohol dependent women. Journal of Substance Abuse Treatment 2017;80:88-97. | Non-relevant digital therapeutic |
| 5 | Actrn. An internet-delivered, evidenced-based treatment program for mental health and alcohol use in Older Australians. https://trialsearch.who.int/Trial2.aspx?TrialID=ACTRN12620000366954 2020. | Non-US language/non-US geography/non-human study |
| 6 | Actrn. I bet theres an app for that: Using mental health apps to manage anxiety and depression. https://trialsearch.who.int/Trial2.aspx?TrialID=ACTRN12619001302145 2019. | Non-US language/non-US geography/non-human study |
| 7 | Actrn. Pilot randomised controlled trial of a real time, personalised, transdiagnostic smartphone intervention targeting repetitive negative thinking in young people with depression and anxiety. https://trialsearch.who.int/Trial2.aspx?TrialID=ACTRN12621001701819 2021. | Non-US language/non-US geography/non-human study |
| 8 | Adam A, Jain A, Pletnikova A, et al. Use of a Mobile App to Augment Psychotherapy in a Community Psychiatric Clinic: Feasibility and Fidelity Trial. JMIR Formative Research 2020;4:Non-US language/non-US geography/non-human study 7722. | Non-relevant digital therapeutic |
| 9 | Ahuvia IL, Sung JY, Dobias ML, et al. College student interest in teletherapy and self-guided mental health supports during the COVID-19 pandemic. Journal of American college health : J of ACH 2022:1-7. | Non-relevant outcome |
| 10 | Aizenstros A, Bakker D, Hofmann SG, et al. Engagement with smartphone-delivered behavioural activation interventions: a study of the MoodMission smartphone application. Behavioural and cognitive psychotherapy 2021;49(5):569-581. | Non-US language/non-US geography/non-human study |
| 11 | Albritton T, Ford KL, Elsbernd K, et al. Implementing a peer advocate mental health digital intervention program for Ohio youth: Descriptive pilot study. JMIR Mental Health 2021;8(4) (no pagination). | Non-relevant digital therapeutic |
| 12 | Alfaro AJ, Carlson C, Mehta PS, et al. Geri-mobile health: Feasibility of a VA mental health mobile apps training program for older veterans. Journal of the American Geriatrics Society 2021;69(SUPPL 1):S48. | Non-relevant digital therapeutic |
| 13 | Alinia P, Sah RK, McDonell M, et al. Associations Between Physiological Signals Captured Using Wearable Sensors and Self-reported Outcomes Among Adults in Alcohol Use Disorder Recovery: Development and Usability Study. JMIR Formative Research 2021;5:Non-relevant study design7891. | Non-relevant digital therapeutic |
| 14 | Almklov E, Afari N, Floto E, et al. Post-9/11 Veteran Satisfaction With the VA eScreening Program. Military medicine 2020;185(3-4):519-529. | Non-relevant digital therapeutic |
| 15 | Anton MT, Greenberger HM, Andreopoulos E, et al. Evaluation of a Commercial Mobile Health App for Depression and Anxiety (AbleTo Digital+): Retrospective Cohort Study. JMIR Formative Research 2021;5:Non-relevant study design7570. | Non-relevant outcome |
| 16 | Anton MT, Ridings LE, Hanson R, et al. Hybrid type 1 randomized controlled trial of a tablet-based application to improve quality of care in child mental health treatment. Contemporary Clinical Trials 2020;94 (no pagination). | Non-relevant outcome |
| 17 | Arean PA, Friedman EC, Pratap A, et al. Using Real-world Data for Decision Support: Recommendations from a Primary Care Provider Survey. The Permanente journal 2021;25. | Non-relevant digital therapeutic |
| 18 | Arevian AC, O'Hora J, Rosser J, et al. Patient and Provider Cocreation of Mobile Texting Apps to Support Behavioral Health: Usability Study. JMIR mHealth and uHealth 2020;8(7):e12655. | Non-relevant outcome |
| 19 | Austin SF, Frosig A, Buus N, et al. Service User Experiences of Integrating a Mobile Solution (IMPACHS) Into Clinical Treatment for Psychosis. Qualitative health research 2021;31(5):942-954. | Non-US language/non-US geography/non-human study |
| 20 | Austin SF, Jansen JE, Petersen CJ, et al. Mobile app integration into dialectical behavior therapy for persons with borderline personality disorder: Qualitative and quantitative study. JMIR Mental Health 2020;7(6) (no pagination). | Non-US language/non-US geography/non-human study |
| 21 | Bankole A, Anderson MS, Homdee N, et al. BESI: Behavioral and Environmental Sensing and Intervention for Dementia Caregiver Empowerment-Phases 1 and 2. American Journal of Alzheimer's Disease and other Dementias 2020;35. | Non-relevant outcome |
| 22 | Baseman J, Revere D, Baldwin LM. A Mobile Breast Cancer Survivorship Care App: Pilot Study. JMIR Cancer 2017;3:Non-US language/non-US geography/non-human study 4. | Non-relevant digital therapeutic |
| 23 | Bell IH, Thompson A, Valentine L, et al. Ownership, Use of, and Interest in Digital Mental Health Technologies among Clinicians and Young People across a Spectrum of Clinical Care Needs: Cross-sectional Survey. JMIR Mental Health 2022;9(5) (no pagination). | Non-US language/non-US geography/non-human study |
| 24 | Benda NC, Alexopoulos GS, Marino P, et al. The Age Limit Does Not Exist: A Pilot Usability Assessment of a SMS-Messaging and Smartwatch-Based Intervention for Older Adults with Depression. AMIA ... Annual Symposium Proceedings/AMIA Symposium 2020;2020:213-222. | Non-relevant digital therapeutic |
| 25 | Ben-Zeev D, Razzano LA, Pashka NJ, et al. Cost of mHealth Versus Clinic-Based Care for Serious Mental Illness: Same Effects, Half the Price Tag. Psychiatric Services 2021;72:448-451. | Non-relevant outcome |
| 26 | Bergerot CD, Malhotra J, Bergerot PG, et al. Prospective assessment of a smartphone-app based mindfulness program for patients with metastatic renal cell carcinoma (mRCC). Journal of Clinical Oncology. Conference 2022;40. | Non-US language/non-US geography/non-human study |
| 27 | Bernecker SL, Banschback K, Santorelli GD, et al. A Web-Disseminated Self-Help and Peer Support Program Could Fill Gaps in Mental Health Care: Lessons From a Consumer Survey. JMIR Mental Health 2017;4:Non-relevant outcome. | Non-relevant digital therapeutic |
| 28 | Bondar J, Babich Morrow C, Gueorguieva R, et al. Clinical and Financial Outcomes Associated With a Workplace Mental Health Program Before and During the COVID-19 Pandemic. JAMA network open 2022;5(6):Non-relevant study design216349. | Non-relevant audience |
| 29 | Borosund E, Ehlers SL, Clark MM, et al. Digital stress management in cancer: Testing StressProffen in a 12-month randomized controlled trial. Cancer 2022;128(7):1503-1512. | Non-relevant outcome |
| 30 | Borosund E, Ehlers SL, Varsi C, et al. Results from a randomized controlled trial testing StressProffen; an application-based stress-management intervention for cancer survivors. Cancer Medicine 2020;9(11):3775-3785. | Non-US language/non-US geography/non-human study |
| 31 | Borosund E, Mirkovic J, Clark MM, et al. A Stress Management App Intervention for Cancer Survivors: Design, Development, and Usability Testing. JMIR Formative Research 2018;2:Non-US language/non-US geography/non-human study 9. | Non-US language/non-US geography/non-human study |
| 32 | Borosund E, Mirkovic J, Clark MM, et al. StressProffen: developing and pilot testing a stress management intervention app for patients with cancer. Psycho-oncology 2017;26:94‐. | Non-US language/non-US geography/non-human study |
| 33 | Borosund E, Varsi C, Ehlers SL, et al. Cancer survivor feedback during the RCT of an app based stress management program: Stressproffen. Psycho-Oncology 2020;29(Supplement 1):84. | Non-US language/non-US geography/non-human study |
| 34 | Borrione L, Cirillo PC, Aparicio LV, et al. A study protocol for an ongoing multi-arm, randomized, double-blind, sham-controlled clinical trial with digital features, using portable transcranial electrical stimulation and internet-based behavioral therapy for major depression disorders: The PSYLECT study. Expert Review of Neurotherapeutics 2022:1-11. | Non-US language/non-US geography/non-human study |
| 35 | Boskovic D, Liberman J, Wiggins E, et al. Early experience with aripiprazole tablets with sensor: patient characteristics, utilization, and cost from real-world data. Journal of Managed Care and Specialty Pharmacy 2022;28(3-A Supplement):S42. | Non-relevant outcome |
| 36 | Bostock S, Crosswell AD, Prather AA, et al. Mindfulness on-the-go: Effects of a mindfulness meditation app on work stress and well-being. Journal of Occupational Health Psychology 2019;24:127‐138. | Non-relevant outcome |
| 37 | Bostrom K, Borosund E, Varsi C, et al. Digital Self-Management in Support of Patients Living With Chronic Pain: Feasibility Pilot Study. JMIR Formative Research 2020;4:Non-relevant study design3893. | Non-US language/non-US geography/non-human study |
| 38 | Bouchard LC, Yanez B, Dahn JR, et al. Brief report of a tablet-delivered psychosocial intervention for men with advanced prostate cancer: Acceptability and efficacy by race. Translational Behavioral Medicine 2019;9(4):629-637. | Non-relevant outcome |
| 39 | Brassil KJ, Patterson J, Allison M, et al. Comorbidities and patient-reported outcomes among individuals with cancer enrolled in a digital health coaching program. Journal of Clinical Oncology. Conference 2020;38. | Non-relevant outcome |
| 40 | Breitenstein SM, Brager J, Ocampo EV, et al. Engagement and Adherence With ezPARENT, an mHealth Parent-Training Program Promoting Child Well-Being. Child maltreatment 2017;22(4):295-304. | Non-relevant outcome |
| 41 | Brewer JA, Roy A, Deluty A, et al. Can mindfulness mechanistically target worry to improve sleep disturbances? Theory and study protocol for app-based anxiety program. Health Psychology 2020;39(9):776-784. | Non-relevant outcome |
| 42 | Bricker JB, Watson NL, Heffner JL, et al. A Smartphone App Designed to Help Cancer Patients Stop Smoking: Results From a Pilot Randomized Trial on Feasibility, Acceptability, and Effectiveness. JMIR Formative Research 2020;4:Non-US language/non-US geography/non-human study 6652. | Non-relevant digital therapeutic |
| 43 | Brook R, Sax MJ, Carlisle JA, et al. Pns111 Survey Results of Contemporary U.S. Health Plan Policies: New Challenges, Digital Technologies, Value-Based Contracting, and Comparative Effectiveness Research. Value in Health 2019;22(Supplement 2):S304. | Non-relevant outcome |
| 44 | Buis LR, McCant FA, Gierisch JM, et al. Understanding the Effect of Adding Automated and Human Coaching to a Mobile Health Physical Activity App for Afghanistan and Iraq Veterans: Protocol for a Randomized Controlled Trial of the Stay Strong Intervention. JMIR Research Protocols 2019;8:Non-US language/non-US geography/non-human study 2526. | Non-relevant digital therapeutic |
| 45 | Bush NE, Smolenski DJ, Denneson LM, et al. A Virtual Hope Box: Randomized controlled trial of a smartphone app for emotional regulation and coping with distress. Psychiatric Services 2017;68(4):330-336. | Non-relevant outcome |
| 46 | Businelle MS, Garey L, Gallagher MW, et al. An Integrated mHealth App for Smoking Cessation in Black Smokers With Anxiety: Protocol for a Randomized Controlled Trial. JMIR Research Protocols 2022;11:Non-relevant digital solution 8905. | Non-relevant outcome |
| 47 | Camacho E, Torous J. Interest and readiness for digital mental health in coordinate specialty care for early course psychosis: A survey study of 42 programs in 30 states. Early Intervention in Psychiatry 2021;15(5):1243-1255. | Non-relevant outcome |
| 48 | Caplan S, Sosa Lovera A, Veloz Comas E, et al. A Mobile App to Prevent Depression Among Low-Income Primary Care Patients in the Dominican Republic: Sociocultural Adaptations. Journal of Transcultural Nursing 2020;31:413-424. | Non-US language/non-US geography/non-human study |
| 49 | Carlson C, Kuhn E, Nazem S, et al. Mobile intervention for depression benefits middle aged and older adults. Journal of the American Geriatrics Society 2020;68(SUPPL 1):S255-S256. | Non-relevant outcome |
| 50 | Carpenter-Song E, Acquilano SC, Noel V, et al. Individualized Intervention to Support Mental Health Recovery Through Implementation of Digital Tools into Clinical Care: Feasibility Study. Community mental health journal 2022;58(1):99-110. | Non-relevant outcome |
| 51 | Carullo PC, Ungerman EA, Metro DG, et al. The impact of a smartphone meditation application on anesthesia trainee well-being. Journal of Clinical Anesthesia 2021;75 (no pagination). | Non-relevant outcome |
| 52 | Casey Orr L, Graham AK, Mohr DC, et al. Engagement and clinical improvement among older adult primary care patients using a mobile intervention for depression and anxiety: case studies. JMIR mental health 2020;7. | Non-relevant study design |
| 53 | Cavazos-Rehg P, Min C, Fitzsimmons-Craft EE, et al. Parental consent: A potential barrier for underage teens' participation in an mHealth mental health intervention. Internet Interventions 2020;21 (no pagination). | Non-relevant outcome |
| 54 | Champion L, Economides M, Chandler C. The efficacy of a brief app-based mindfulness intervention on psychosocial outcomes in healthy adults: A pilot randomised controlled trial. PLoS ONE 2018;13(12) (no pagination). | Non-relevant digital therapeutic |
| 55 | Chan WW, Fitzsimmons-Craft EE, Smith AC, et al. The Challenges in Designing a Prevention Chatbot for Eating Disorders: Observational Study. JMIR Formative Research 2022;6:Non-relevant study design8003. | Non-relevant outcome |
| 56 | Cheung EO, Addington EL, Bassett SM, et al. A Self-Paced, Web-Based, Positive Emotion Skills Intervention for Reducing Symptoms of Depression: Protocol for Development and Pilot Testing of MARIGOLD. JMIR Research Protocols 2018;7:Non-US language/non-US geography/non-human study 0494. | Non-relevant outcome |
| 57 | Cheung K, Ling W, Karr CJ, et al. Evaluation of a recommender app for apps for the treatment of depression and anxiety: An analysis of longitudinal user engagement. Journal of the American Medical Informatics Association 2018;25(8):955-962. | Non-relevant outcome |
| 58 | Chu A, Rose TM, Gundrum DA, et al. Evaluating the effects of a mindfulness mobile application on student pharmacists' stress, burnout, and mindfulness. American Journal of Health-System Pharmacy 2022;79(8):656-664. | Non-relevant outcome |
| 59 | Chung AH, Gevirtz RN, Gharbo RS, et al. Pilot Study on Reducing Symptoms of Anxiety with a Heart Rate Variability Biofeedback Wearable and Remote Stress Management Coach. Applied psychophysiology and biofeedback 2021;46(4):347-358. | Non-relevant outcome |
| 60 | Church D, Stapleton P, Sabot D. App-Based Delivery of Clinical Emotional Freedom Techniques: Cross-Sectional Study of App User Self-Ratings. JMIR mHealth and uHealth 2020;8(10):Non-US language/non-US geography/non-human study 8545. | Non-relevant outcome |
| 61 | Cibrian FL, Monteiro E, Ankrah E, et al. Parents' perspectives on a smartwatch intervention for children with ADHD: Rapid deployment and feasibility evaluation of a pilot intervention to support distance learning during COVID-19. PLoS ONE 2021;16(10 October) (no pagination). | Non-relevant outcome |
| 62 | Cochran A, Hoel S, Victory A, et al. A pilot micro-randomized trial for optimizing the delivery of mobile ACT-inspired interventions. Bipolar Disorders 2021;23(SUPPL 1):19. | Non-relevant outcome |
| 63 | Cochran J, Fang H, Gallo CL, et al. P.0508 Correlation between patient engagement with a digital medicine system and clinical symptom improvement. European Neuropsychopharmacology 2021;53(Supplement 1):S374. | Non-relevant outcome |
| 64 | Cohen EA, Skubiak T, Hadzi Boskovic D, et al. Phase 3b Multicenter, Prospective, Open-label Trial to Evaluate the Effects of a Digital Medicine System on Inpatient Psychiatric Hospitalization Rates for Adults With Schizophrenia. Journal of Clinical Psychiatry 2022;83:11. | Non-relevant outcome |
| 65 | Cohen KA, Schleider JL. Adolescent dropout from brief digital mental health interventions within and beyond randomized trials. Internet Interventions 2022;27 (no pagination). | Non-relevant audience |
| 66 | Cohen KA, Stiles-Shields C, Winquist N, et al. Traditional and Nontraditional Mental Healthcare Services: Usage and Preferences Among Adolescents and Younger Adults. The journal of behavioral health services & research. 2021;20. | Non-relevant outcome |
| 67 | Coifman KG, Disabato DD, Seah THS, et al. Boosting positive mood in medical and emergency personnel during the COVID-19 pandemic: Preliminary evidence of efficacy, feasibility and acceptability of a novel online ambulatory intervention. Occupational and Environmental Medicine 2021;78(8):541-547. | Non-relevant outcome |
| 68 | Cook SH, Wood EP, Mirin N, et al. A Mindfulness-Based Intervention to Alleviate Stress From Discrimination Among Young Sexual and Gender Minorities of Color: Protocol for a Pilot Optimization Trial. JMIR Research Protocols 2022;11:Non-relevant digital solution 5593. | Non-US language/non-US geography/non-human study |
| 69 | Cosgrove V, Gliddon E, Berk L, et al. Implications of MoodSwings 2.0 for teen-focused eHealth applications. Bipolar Disorders 2019;21(Supplement 1):43. | Non-relevant outcome |
| 70 | Cox CE, Hough C, Jones D, et al. Effect of a self-directed mobile app mindfulness program for ICU survivors: a pilot RCT. American journal of respiratory and critical care medicine 2018;197. | Non-US language/non-US geography/non-human study |
| 71 | Cox CE, Olsen MK, Gallis JA, et al. Optimizing a self-directed mobile mindfulness intervention for improving cardiorespiratory failure survivors' psychological distress (LIFT2): Design and rationale of a randomized factorial experimental clinical trial. Contemporary clinical trials 2020;96:106119. | Non-relevant outcome |
| 72 | Craig SL, Leung VWY, Pascoe R, et al. Affirm online: Utilising an affirmative cognitive-behavioural digital intervention to improve mental health, access, and engagement among LGBTQA+ youth and young adults. International Journal of Environmental Research and Public Health 2021;18(4):1-18. | Non-relevant digital therapeutic |
| 73 | Dahl K, Starr-Glass L. Mindfulness-based virtual reality app for youths on dialysis: Lessons learned from a pilot study. Hemodialysis International 2019;23(1):A23. | Non-relevant outcome |
| 74 | Dahne J, Lejuez CW, Kustanowitz J, et al. Moodivate: A self-help behavioral activation mobile app for utilization in primary care-Development and clinical considerations. International Journal of Psychiatry in Medicine 2017;52(2):160-175. | Non-relevant outcome |
| 75 | Davidson S, Fletcher S, Wadley G, et al. A Mobile Phone App to Improve the Mental Health of Taxi Drivers: Single-Arm Feasibility Trial. JMIR mHealth and uHealth 2020;8(1):Non-US language/non-US geography/non-human study 3133. | Non-US language/non-US geography/non-human study |
| 76 | Davis NO, Bower J, Kollins SH. Proof-of-concept study of an at-home, engaging, digital intervention for pediatric ADHD. PLoS ONE 2018;13(1) (no pagination). | Non-relevant outcome |
| 77 | de Chantal PL, Chagnon A, Cardinal M, et al. Evidence of User-Expert Gaps in Health App Ratings and Implications for Practice. Frontiers in Digital Health 2022;4:765993. | Non-relevant outcome |
| 78 | Desrosiers A, Schafer C, Esliker R, et al. mHealth-Supported Delivery of an Evidence-Based Family Home-Visiting Intervention in Sierra Leone: Protocol for a Pilot Randomized Controlled Trial. JMIR Research Protocols 2021;10:Non-relevant study design5443. | Non-US language/non-US geography/non-human study |
| 79 | Dietvorst E, Legerstee JS, Vreeker A, et al. The Grow It! app-longitudinal changes in adolescent well-being during the COVID-19 pandemic: a proof-of-concept study. European Child and Adolescent Psychiatry. 2022. | Non-US language/non-US geography/non-human study |
| 80 | DiNardo MM, Greco C, Phares AD, et al. Effects of an integrated mindfulness intervention for veterans with diabetes distress: a randomized controlled trial. BMJ open diabetes research & care 2022;10. | Non-relevant outcome |
| 81 | DiRenzo DD, Hunt C, Sibinga EM, et al. Feasibility and acceptability of using a meditation app in adults with rheumatic disease. Explore: The Journal of Science & Healing 2021;09:09. | Non-relevant digital therapeutic |
| 82 | Dogrucu A, Perucic A, Isaro A, et al. Moodable: On feasibility of instantaneous depression assessment using machine learning on voice samples with retrospectively harvested smartphone and social media data. Smart Health 2020;17 (no pagination). | Non-relevant outcome |
| 83 | Dopke CA, McBride A, Babington P, et al. Development of Coaching Support for LiveWell: A Smartphone-Based Self-Management Intervention for Bipolar Disorder. JMIR Formative Research 2021;5:Non-relevant study design5810. | Non-relevant digital therapeutic |
| 84 | Doty MS, Chen HY, Ajishegiri O, et al. 251 Mindful meditation for anxiety in individuals admitted to the antepartum unit: a randomized controlled trial. American Journal of Obstetrics and Gynecology 2021;224(2 Supplement):S166. | Non-relevant outcome |
| 85 | Drks. Efficacy and acceptability of the Internet-based self-help program "Lenio" for individuals with chronic pain and depressive symptoms: a randomized controlled trial. https://trialsearch.who.int/Trial2.aspx?TrialID=DRKS00026722 2021. | Non-US language/non-US geography/non-human study |
| 86 | Dvorsky MR, Spiess MI. 42.2 Development and Usability of Advanced Tools for Organization Management: A Mobile Platform to Promote Adolescents' Engagement in Treatment for Adhd. Journal of the American Academy of Child and Adolescent Psychiatry 2020;59(10 Supplement):S330. | Non-relevant outcome |
| 87 | Economides M, Martman J, Bell MJ, et al. Improvements in Stress, Affect, and Irritability Following Brief Use of a Mindfulness-based Smartphone App: A Randomized Controlled Trial. Mindfulness 2018;9:1584-1593. | Non-relevant outcome |
| 88 | Egger HL, Verduin TL, Robinson S, et al. 13.5 the Wonder of It All: Early Childhood Digital Health. Journal of the American Academy of Child and Adolescent Psychiatry 2019;58(10 Supplement):S319. | Non-relevant outcome |
| 89 | Egger HL, Verduin TL, Robinson S, et al. 5.6 Children's Digital Mental Health: A Design and Ethical Framework. Journal of the American Academy of Child and Adolescent Psychiatry 2019;58(10 Supplement):S139. | Non-relevant outcome |
| 90 | Elbogen EB, Dennis PA, Van Voorhees EE, et al. Cognitive Rehabilitation with Mobile Technology and Social Support for Veterans with TBI and PTSD: A Randomized Clinical Trial. Journal of Head Trauma Rehabilitation 2019;34(1):1-10. | Non-relevant outcome |
| 91 | Epstein JN, Lutz J, DeLoss D, et al. 5.13 EXPLORING ENGAGEMENT AS A FACTOR FOR EFFICACY WITH AKL-T01, A HOME-BASED DIGITAL THERAPEUTIC. Journal of the American Academy of Child and Adolescent Psychiatry 2020;59:S153‐. | Non-relevant digital therapeutic |
| 92 | Faro JM, Mattocks KM, Nagawa CS, et al. Physical activity, mental health, and technology preferences to support cancer survivors during the COVID-19 pandemic: Cross-sectional study. JMIR Cancer 2021;7(1) (no pagination). | Non-relevant digital therapeutic |
| 93 | Fitzsimmons-Craft EE, Taylor CB, Newman MG, et al. Harnessing mobile technology to reduce mental health disorders in college populations: A randomized controlled trial study protocol. Contemporary Clinical Trials 2021;103 (no pagination). | Non-relevant outcome |
| 94 | Ford Ii JH, Dodds D, Hyland J, et al. Evaluating the Impact of Music & Memory's Personalized Music and Tablet Engagement Program in Wisconsin Assisted Living Communities: Pilot Study. 24058297 2019;2:Non-US language/non-US geography/non-human study 1599. | Non-relevant digital therapeutic |
| 95 | Forman-Hoffman VL, Nelson BW, Ranta K, et al. Significant reduction in depressive symptoms among patients with moderately-severe to severe depressive symptoms after participation in a therapist-supported, evidence-based mobile health program delivered via a smartphone app. Internet Interventions 2021;25 (no pagination). | Non-relevant digital therapeutic |
| 96 | Fortuna K, Barr P, Goldstein C, et al. Application of Community-Engaged Research to Inform the Development and Implementation of a Peer-Delivered Mobile Health Intervention for Adults With Serious Mental Illness. Journal of Participatory Medicine 2019;11:Non-US language/non-US geography/non-human study 2380. | Non-relevant audience |
| 97 | Fortuna KL, Aschbrenner KA, Lohman MC, et al. Smartphone Ownership, Use, and Willingness to Use Smartphones to Provide Peer-Delivered Services: Results from a National Online Survey. Psychiatric Quarterly 2018;89:947-956. | Non-relevant outcome |
| 98 | Fortuna KL, Lohman MC, Gill LE, et al. Adapting a Psychosocial Intervention for Smartphone Delivery to Middle-Aged and Older Adults with Serious Mental Illness. American Journal of Geriatric Psychiatry 2017;25:819-828. | Non-relevant study design |
| 99 | Franco OH, Calkins ME, Giorgi S, et al. Evidence for feasibility of mobile health and social media-based interventions for early psychosis and clinical high risk. medRxiv. 2022;01. | Non-relevant digital therapeutic |
| 100 | Frenkel A, Winsberg M. In this paper we report the results of a pragmatic retrospective study aiming to test the effectiveness of a mobile phone delivered ACT based AI conversational coaching platform. In the on-boarding measurement half of all participants reported a WHO-5 score of 40 or less, well below the cut-off point of 50, and an indicator of poor well-being. However, on the last measurement recorded for each user half of all participants indicated a score of 52 and above, indicating a change for the better and an overall good well-being. These results highlight the great potential ACT-based mobile apps can have for improving users' daily well-being. JMIR Formative Research 2022;20:20. | Non-relevant outcome |
| 101 | Fulford D, Gard DE, Mueser KT, et al. Preliminary outcomes of an ecological momentary intervention for social functioning in schizophrenia: Pre-post study of the motivation and skills support app. JMIR Mental Health 2021;8(6) (no pagination). | Non-relevant outcome |
| 102 | Fuller-Tyszkiewicz M, Richardson B, Klein B, et al. A Mobile App-Based Intervention for Depression: End-User and Expert Usability Testing Study. JMIR Mental Health 2018;5:Non-relevant outcome4. | Non-US language/non-US geography/non-human study |
| 103 | Ghahramani F, Wang J. Intention to Adopt mHealth Apps Among Informal Caregivers: Cross-Sectional Study. JMIR mHealth and uHealth 2021;9(3):Non-relevant study design4755. | Non-relevant digital therapeutic |
| 104 | Golden EA, Zweig M, Danieletto M, et al. A Resilience-Building App to Support the Mental Health of Health Care Workers in the COVID-19 Era: Design Process, Distribution, and Evaluation. JMIR Formative Research 2021;5:Non-relevant study design6590. | Non-relevant outcome |
| 105 | Gould CE, Loup J, Kuhn E, et al. Technology use and preferences for mental health self-management interventions among older veterans. International Journal of Geriatric Psychiatry 2020;35(3):321-330. | Non-relevant digital therapeutic |
| 106 | Gowarty MA, Kung NJ, Maher AE, et al. Perceptions of Mobile Apps for Smoking Cessation Among Young People in Community Mental Health Care: Qualitative Study. JMIR Formative Research 2020;4:Non-US language/non-US geography/non-human study 9860. | Non-relevant digital therapeutic |
| 107 | Grande D, Luna Marti X, Merchant RM, et al. Consumer Views on Health Applications of Consumer Digital Data and Health Privacy Among US Adults: Qualitative Interview Study. Journal of Medical Internet Research 2021;23:Non-relevant study design9395. | Non-relevant digital therapeutic |
| 108 | Guo Y, Yang F, Hu F, et al. Existing Mobile Phone Apps for Self-Care Management of People With Alzheimer Disease and Related Dementias: Systematic Analysis. 24058297 2020;3:Non-US language/non-US geography/non-human study 5290. | Non-relevant study design |
| 109 | Gutner CA, Pedersen ER, Drummond SPA. Going direct to the consumer: Examining treatment preferences for veterans with insomnia, PTSD, and depression. Psychiatry Research 2018;263:108-114. | Non-relevant outcome |
| 110 | Heilemann MV, Martinez A, Soderlund PD. A Mental Health Storytelling Intervention Using Transmedia to Engage Latinas: Grounded Theory Analysis of Participants' Perceptions of the Story's Main Character. Journal of medical Internet research 2018;20(5):Non-US language/non-US geography/non-human study 0028. | Non-relevant outcome |
| 111 | Heron KE, Romano KA, Braitman AL. Mobile technology use and mHealth text message preferences: an examination of gender, racial, and ethnic differences among emerging adult college students. Began with 2015 2019;5:2. | Non-relevant digital therapeutic |
| 112 | Hoel S, Victory A, Sagorac Gruichich T, et al. A Mixed-Methods Analysis of Mobile ACT Responses From Two Cohorts. Frontiers in Digital Health 2022;4:869143. | Non-relevant digital therapeutic |
| 113 | Huberty J, Eckert R, Larkey L, et al. Smartphone-Based Meditation for Myeloproliferative Neoplasm Patients: Feasibility Study to Inform Future Trials. JMIR Formative Research 2019;3:Non-US language/non-US geography/non-human study 2662. | Non-relevant outcome |
| 114 | Huberty J, Vranceanu AM, Carney C, et al. Characteristics and Usage Patterns Among 12,151 Paid Subscribers of the Calm Meditation App: Cross-Sectional Survey. JMIR mHealth and uHealth 2019;7(11):Non-US language/non-US geography/non-human study 5648. | Non-relevant outcome |
| 115 | Huckvale K, Torous J, Larsen ME. Assessment of the Data Sharing and Privacy Practices of Smartphone Apps for Depression and Smoking Cessation. JAMA Network Open 2019;2(4) (no pagination). | Non-relevant audience |
| 116 | Hunter JE, Jenkins CL, Grim V, et al. Feasibility of an app-based mindfulness intervention among women with an FMR1 premutation experiencing maternal stress. Research in Developmental Disabilities 2019;89:76-82. | Non-relevant outcome |
| 117 | Imtiaz D, Khan A, Seelye A. A Mobile Multimedia Reminiscence Therapy Application to Reduce Behavioral and Psychological Symptoms in Persons with Alzheimer's. Journal of Healthcare Engineering 2018;2018:1536316. | Non-relevant outcome |
| 118 | Isrctn. Supporting employees with insomnia and emotional regulation problems. https://trialsearch.who.int/Trial2.aspx?TrialID=ISRCTN13596153 2021. | Non-relevant outcome |
| 119 | Jain FA, Okereke O, Gitlin L, et al. Mentalizing imagery therapy to augment skills training for dementia caregivers: protocol for a randomized, controlled trial of a mobile application and digital phenotyping. Contemporary clinical trials 2022;116. | Non-relevant outcome |
| 120 | Jaworski BK, Taylor K, Ramsey KM, et al. Exploring usage of COVID coach, a public mental health app designed for the COVID-19 pandemic: Evaluation of analytics data. Journal of Medical Internet Research 2021;23(3) (no pagination). | Non-relevant outcome |
| 121 | Jessen S, Mirkovic J, Halvorsen Brendmo E, et al. Evaluating a Strengths-Based mHealth Tool (MyStrengths): Explorative Feasibility Trial. JMIR Formative Research 2021;5:Non-relevant digital solution 0572. | Non-US language/non-US geography/non-human study |
| 122 | Johansen SL, Olmert T, Chaudhary N, et al. Incorporating Digital Interventions into Mental Health Clinical Practice: a Pilot Survey of How Use Patterns, Barriers, and Opportunities Shifted for Clinicians in the COVID-19 Pandemic. Journal of Technology in Behavioral Science 2022:1-5. | Non-relevant audience |
| 123 | Johnstone JM, Ribbers A, Jenkins D, et al. Classroom-Based Mindfulness Training Reduces Anxiety in Adolescents: Acceptability and Effectiveness of a Cluster-Randomized Pilot Study. Journal of Restorative Medicine 2020;10. | Non-relevant outcome |
| 124 | Jonathan G, Goulding E, Dopke C, et al. Understand the mechanisms of behavior change in LiveWell: a smartphone intervention for bipolar disorder. Bipolar disorders 2020;22:120‐121. | Non-relevant outcome |
| 125 | Jones C, O'Toole K, Jones K, et al. Quality of Psychoeducational Apps for Military Members With Mild Traumatic Brain Injury: An Evaluation Utilizing the Mobile Application Rating Scale. JMIR mHealth and uHealth 2020;8(8):Non-US language/non-US geography/non-human study 9807. | Non-US language/non-US geography/non-human study |
| 126 | Jurigova BG, Gerdes MR, Anguera JA, et al. Sustained benefits of cognitive training in children with inattention, three-year follow-up. PLoS ONE 2021;16(2 February) (no pagination). | Non-relevant outcome |
| 127 | Kaplan A, Mannarino AP, Nickell PV. Evaluating the Impact of Freespira on Panic Disorder Patients' Health Outcomes and Healthcare Costs within the Allegheny Health Network. Applied psychophysiology and biofeedback 2020;45(3):175-181. | Non-relevant outcome |
| 128 | Kato Y, Kageyama K, Mesaki T, et al. Study protocol for a pilot randomized controlled trial on a smartphone application-based intervention for subthreshold depression: study protocol clinical trial (SPIRIT Compliant). Medicine (united states) 2020;99. | Non-US language/non-US geography/non-human study |
| 129 | Kim HM, Xu Y, Wang Y. Overcoming the Mental Health Stigma Through m-Health Apps: Results from the Healthy Minds Study. Telemedicine journal and e health : the official journal of the American Telemedicine Association. 2022;04. | Non-relevant outcome |
| 130 | Kozlov E, McDarby M, Prescott M, et al. Assessing the Care Modality Preferences and Predictors for Digital Mental Health Treatment Seekers in a Technology-Enabled Stepped Care Delivery System: Cross-sectional Study. JMIR Formative Research 2021;5:Non-relevant digital solution 0162. | Non-relevant outcome |
| 131 | Krafft J, Ong CW, Davis CH, et al. An Open Trial of Group Acceptance and Commitment Therapy With an Adjunctive Mobile App for Generalized Anxiety Disorder. Cognitive and Behavioral Practice. 2021. | Non-relevant outcome |
| 132 | Kroska EB, Hoel S, Victory A, et al. Optimizing an Acceptance and Commitment Therapy Microintervention Via a Mobile App With Two Cohorts: Protocol for Micro-Randomized Trials. JMIR Research Protocols 2020;9:Non-US language/non-US geography/non-human study 7086. | Non-relevant outcome |
| 133 | Kubo A, Kurtovich E, McGinnis M, et al. Pilot pragmatic randomized trial of mHealth mindfulness-based intervention for advanced cancer patients and their informal caregivers. Psycho Oncology. 2020. | Non-relevant outcome |
| 134 | Kwasny MJ, Schueller SM, Lattie E, et al. Exploring the Use of Multiple Mental Health Apps Within a Platform: Secondary Analysis of the IntelliCare Field Trial. JMIR Mental Health 2019;6:Non-US language/non-US geography/non-human study 1572. | Non-relevant outcome |
| 135 | Lambert SD, Duncan LR, Culos-Reed SN, et al. Feasibility, Acceptability, and Clinical Significance of a Dyadic, Web-Based, Psychosocial and Physical Activity Self-Management Program (TEMPO) Tailored to the Needs of Men with Prostate Cancer and Their Caregivers: A Multi-Center Randomized Pilot Trial. Current Oncology 2022;29(2):785-804. | Non-US language/non-US geography/non-human study |
| 136 | Lancioni GE, Singh NN, O'Reilly MF, et al. Smartphone-Based Interventions to Foster Simple Activity and Personal Satisfaction in People With Advanced Alzheimer's Disease. American Journal of Alzheimer's Disease & Other Dementias 2019;34:478-485. | Non-US language/non-US geography/non-human study |
| 137 | Li H, Yang S, Chi H, et al. Enhancing attention and memory of individuals at clinical high risk for psychosis with mHealth technology. Asian Journal of Psychiatry 2021;58 (no pagination). | Non-US language/non-US geography/non-human study |
| 138 | Lim MH, Gleeson JFM, Rodebaugh TL, et al. A pilot digital intervention targeting loneliness in young people with psychosis. Social psychiatry and psychiatric epidemiology 2020;55(7):877-889. | Non-US language/non-US geography/non-human study |
| 139 | Lim MH, Rodebaugh TL, Eres R, et al. A Pilot Digital Intervention Targeting Loneliness in Youth Mental Health. Frontiers in Psychiatry 2019;10 (no pagination). | Non-US language/non-US geography/non-human study |
| 140 | Lipschitz JM, Connolly SL, Miller CJ, et al. Patient interest in mental health mobile app interventions: Demographic and symptom-level differences. Journal of Affective Disorders 2020;263:216-220. | Non-relevant outcome |
| 141 | Lopez G, Chaoul A, Christie A, et al. Self-administered meditation application (APP) for cancer patients reporting psychosocial distress: A pilot study. Supportive Care in Cancer 2021;29(SUPPL 1):S49-S50. | Non-relevant outcome |
| 142 | Mande A, Moore SL, Banaei-Kashani F, et al. Assessment of a Mobile Health iPhone App for Semiautomated Self-management of Chronic Recurrent Medical Conditions Using an N-of-1 Trial Framework: Feasibility Pilot Study. JMIR Formative Research 2022;6:Non-relevant digital solution 4827. | Non-relevant digital therapeutic |
| 143 | McGinnis RS, McGinnis EW, Petrillo C, et al. Validation of Smartphone Based Heart Rate Tracking for Remote Treatment of Panic Attacks. IEEE Journal of Biomedical and Health Informatics 2021;25(3):656-662. | Non-relevant outcome |
| 144 | Meheli S, Sinha C, Kadaba M. Understanding People With Chronic Pain Who Use a Cognitive Behavioral Therapy-Based Artificial Intelligence Mental Health App (Wysa): Mixed Methods Retrospective Observational Study. JMIR Human Factors 2022;9:Non-relevant digital solution 5671. | Non-relevant digital therapeutic |
| 145 | Melcher J, Torous J. Smartphone Apps for College Mental Health: A Concern for Privacy and Quality of Current Offerings. Psychiatric Services 2020;71(11):1114-1119. | Non-relevant outcome |
| 146 | Mercurio M, Larsen M, Wisniewski H, et al. Longitudinal trends in the quality, effectiveness and attributes of highly rated smartphone health apps. Evidence-based mental health 2020;23(3):107-111. | Non-relevant outcome |
| 147 | Meyer N, Kerz M, Folarin A, et al. Capturing Rest-Activity Profiles in Schizophrenia Using Wearable and Mobile Technologies: Development, Implementation, Feasibility, and Acceptability of a Remote Monitoring Platform. JMIR MHealth and UHealth 2018;6:Non-US language/non-US geography/non-human study 88. | Non-US language/non-US geography/non-human study |
| 148 | Migoya-Borja M, Delgado-Gomez D, Carmona-Camacho R, et al. Feasibility of a Virtual Reality-Based Psychoeducational Tool (VRight) for Depressive Patients. Cyberpsychology, behavior and social networking 2020;23(4):246-252. | Non-US language/non-US geography/non-human study |
| 149 | Miklowitz D, Arevian A, Walshaw P. Technology-enhanced family intervention for adolescents at risk for mood disorders. Bipolar Disorders 2019;21(Supplement 1):52. | Non-relevant outcome |
| 150 | Minen MT, Gopal A, Sahyoun G, et al. The functionality, evidence, and privacy issues around smartphone apps for the top neuropsychiatric conditions. Journal of Neuropsychiatry and Clinical Neurosciences 2021;33(1):72-79. | Non-relevant audience |
| 151 | Minen MT, Jalloh A, Ortega E, et al. User design and experience preferences in a novel smartphone application for migraine management: A think aloud study of the RELAXaHEAD application. Pain Medicine (United States) 2019;20(2):369-377. | Non-relevant digital therapeutic |
| 152 | Molleda L, Bahamon M, St George SM, et al. Clinic Personnel, Facilitator, and Parent Perspectives of eHealth Familias Unidas in Primary Care. Journal of pediatric health care : official publication of National Association of Pediatric Nurse Associates & Practitioners 2017;31(3):350-361. | Non-relevant digital therapeutic |
| 153 | Morris RR, Kouddous K, Kshirsagar R, et al. Towards an Artificially Empathic Conversational Agent for Mental Health Applications: System Design and User Perceptions. Journal of medical Internet research 2018;20(6):Non-US language/non-US geography/non-human study 0148. | Non-relevant outcome |
| 154 | Morrison Wylde C, Mahrer NE, Meyer RML, et al. Mindfulness for Novice Pediatric Nurses: Smartphone Application Versus Traditional Intervention. Journal of pediatric nursing 2017;36:205-212. | Non-relevant outcome |
| 155 | Morton E, Torous J, Murray G, et al. Using apps for bipolar disorder - An online survey of healthcare provider perspectives and practices. Journal of Psychiatric Research 2021;137:22-28. | Non-US language/non-US geography/non-human study |
| 156 | Mruzek DW, McAleavey S, Loring WA, et al. A pilot investigation of an iOS-based app for toilet training children with autism spectrum disorder. Autism 2019;23(2):359-370. | Non-relevant digital therapeutic |
| 157 | Nct. Acceptability/Feasibility of IU Intervention. https://clinicaltrials.gov/show/NCT05172427 2021. | Non-relevant digital therapeutic |
| 158 | Nct. BRAVE Study: Designing and Evaluating Technologies to Promote Adolescent Mental Health. https://clinicaltrials.gov/show/NCT04979481 2021. | Non-relevant outcome |
| 159 | Nct. Culturally Responsive Stress Reduction: A Mobile Mindfulness Application for African Americans (CRSR). https://clinicaltrials.gov/show/NCT04000841 2019. | Non-relevant outcome |
| 160 | Nct. Digital Behavioral Therapy for Sleep Problems. https://clinicaltrials.gov/show/NCT04308499 2020. | Non-relevant outcome |
| 161 | Nct. Effects of Digital Stories Intervention on Psychosocial Well-being. https://clinicaltrials.gov/show/NCT03654599 2018. | Non-relevant digital therapeutic |
| 162 | Nct. Implementing mHealth for Schizophrenia in Community Mental Health Settings. https://clinicaltrials.gov/show/NCT04147897 2019. | Non-relevant outcome |
| 163 | Nct. Relaxation Breathing Training for Children. https://clinicaltrials.gov/show/NCT03271086 2017. | Non-relevant outcome |
| 164 | Nct. Resolving Psychological Stress. https://clinicaltrials.gov/show/NCT03996876 2019. | Non-relevant outcome |
| 165 | Ness SL, Bangerter A, Manyakov NV, et al. An observational study with the Janssen Autism knowledge engine (JAKE<sup></sup>) in individuals with autism spectrum disorder. Frontiers in Neuroscience 2019;13(FEB) (no pagination). | Non-relevant digital therapeutic |
| 166 | Ogrodniczuk JS, Beharry J, Oliffe JL. An Evaluation of 5-Year Web Analytics for HeadsUpGuys: A Men's Depression E-Mental Health Resource. American journal of men's health 2021;15(6):15579883211063322. | Non-relevant outcome |
| 167 | Ola C, Gonzalez E, Tran N, et al. Evaluating the Feasibility and Acceptability of the Lifestyle Enhancement for ADHD Program. Journal of pediatric psychology 2021;46(6):662-672. | Non-relevant digital therapeutic |
| 168 | Onyeaka H, Firth J, Kessler RC, et al. Use of smartphones, mobile apps and wearables for health promotion by people with anxiety or depression: An analysis of a nationally representative survey data. Psychiatry Research 2021;304 (no pagination). | Non-relevant outcome |
| 169 | Ostacher MJ, Fischer E, Bowen ER, et al. Investigation of a Capnometry Guided Respiratory Intervention in the Treatment of Posttraumatic Stress Disorder. Applied psychophysiology and biofeedback 2021;46(4):367-376. | Non-relevant outcome |
| 170 | Owens O, McDonnell K, Beer J, et al. P1.16-32 Empowering Lung Cancer Survivors and Family Members to "Breathe Easier": Adaptation and Evaluation of a M-Health Intervention. Journal of Thoracic Oncology 2019;14(10 Supplement):S599-S600. | Non-relevant digital therapeutic |
| 171 | Owens OL, Beer JM, Reyes LI, et al. Mindfulness-Based Symptom and Stress Management Apps for Adults With Chronic Lung Disease: Systematic Search in App Stores. JMIR MHealth and UHealth 2018;6:Non-US language/non-US geography/non-human study 24. | Non-relevant audience |
| 172 | Parker AM, Nelliot A, Chessare CM, et al. Usability and acceptability of a mobile application prototype for a combined behavioural activation and physical rehabilitation intervention in acute respiratory failure survivors. Australian critical care : official journal of the Confederation of Australian Critical Care Nurses 2020;33(6):511-517. | Non-relevant digital therapeutic |
| 173 | Parmar P, Ryu J, Pandya S, et al. Health-focused conversational agents in person-centered care: a review of apps. npj Digital Medicine 2022;5(1) (no pagination). | Non-relevant audience |
| 174 | Parrish EM, Filip TF, Torous J, et al. Are Mental Health Apps Adequately Equipped to Handle Users in Crisis? Crisis. 2021;27. | Non-relevant audience |
| 175 | Peris TS, Walkup JT. Improving the Treatment of Pediatric Anxiety Disorders: New Directions for Research and Practice. Journal of the American Academy of Child and Adolescent Psychiatry 2019;58(10 Supplement):S302. | Non-relevant outcome |
| 176 | Petrinec A, Wilk C, Hughes JW, et al. Delivering Cognitive Behavioral Therapy for Post-Intensive Care Syndrome-Family via a Mobile Health App. American journal of critical care : an official publication, American Association of Critical-Care Nurses 2021;30(6):451-458. | Non-relevant outcome |
| 177 | Pires IM, Marques G, Garcia NM, et al. A research on the classification and applicability of the mobile health applications. Journal of Personalized Medicine 2020;10(1) (no pagination). | Non-relevant digital therapeutic |
| 178 | Possemato K, Wu J, Greene C, et al. Web-Based Problem-solving Training With and Without Peer Support in Veterans With Unmet Mental Health Needs: pilot Study of Feasibility, User Acceptability, and Participant Engagement. Journal of medical Internet research 2022;24. | Non-relevant outcome |
| 179 | Powell AC, Bowman MB, Harbin HT. Reimbursement of Apps for Mental Health: Findings From Interviews. JMIR Mental Health 2019;6:Non-US language/non-US geography/non-human study 4724. | Non-relevant outcome |
| 180 | Pramana G, Parmanto B, Lomas J, et al. Using Mobile Health Gamification to Facilitate Cognitive Behavioral Therapy Skills Practice in Child Anxiety Treatment: Open Clinical Trial. JMIR Serious Games 2018;6:e9. | Non-relevant outcome |
| 181 | Puzia M, Laird B, Green J, et al. Parents' Perceptions of Their Children's Engagement in a Consumer-Based Meditation Mobile App: Cross-Sectional Survey Study. JMIR Pediatrics and Parenting 2020;3:Non-relevant study design4536. | Non-relevant outcome |
| 182 | Rauseo-Ricupero N, Torous J. Technology Enabled Clinical Care (TECC): Protocol for a Prospective Longitudinal Cohort Study of Smartphone-Augmented Mental Health Treatment. JMIR Research Protocols 2021;10:Non-relevant study design3771. | Non-relevant outcome |
| 183 | Reyes AT, Bhatta TR, Muthukumar V, et al. Testing the acceptability and initial efficacy of a smartphone-app mindfulness intervention for college student veterans with PTSD. Archives of psychiatric nursing 2020;34(2):58-66. | Non-relevant outcome |
| 184 | Reyes AT. The Process of Learning Mindfulness and Acceptance through the Use of a Mobile App Based on Acceptance and Commitment Therapy: A Grounded Theory Analysis. Issues in mental health nursing 2022;43(1):3-12. | Non-relevant outcome |
| 185 | Rickardsson J, Gentili C, Holmstrom L, et al. Internet-delivered acceptance and commitment therapy as microlearning for chronic pain: A randomized controlled trial with 1-year follow-up. European Journal of Pain 2021;25:1012-1030. | Non-relevant outcome |
| 186 | Rizzo S, Spruijt-Metz D, Hartholt A, et al. Mobile virtual human health care guides for young adult childhood cancer survivors. Cancer Epidemiology Biomarkers and Prevention. Conference: AACR Special Conference on Modernizing Population Sciences in the Digital Age. San Diego, CA United States 2020;29. | Non-relevant outcome |
| 187 | Robbins R, Krebs P, Jagannathan R, et al. Health App Use Among US Mobile Phone Users: Analysis of Trends by Chronic Disease Status. JMIR MHealth and UHealth 2017;5:Non-US language/non-US geography/non-human study 97. | Non-relevant outcome |
| 188 | Rosen KD, Paniagua SM, Kazanis W, et al. Quality of life among women diagnosed with breast Cancer: A randomized waitlist controlled trial of commercially available mobile app-delivered mindfulness training. Psycho-Oncology 2018;27:2023-2030. | Non-relevant outcome |
| 189 | Rotondi AJ, Grady J, Hanusa BH, et al. Key variables for effective ehealth designs for individuals with and without mental health disorders: 2 12-4 Fractional factorial experiment. Journal of Medical Internet Research 2021;23(3) (no pagination). | Non-relevant outcome |
| 190 | Rotondi AJ, Spring MR, Hanusa BH, et al. Designing eHealth Applications to Reduce Cognitive Effort for Persons With Severe Mental Illness: Page Complexity, Navigation Simplicity, and Comprehensibility. JMIR Human Factors 2017;4:Non-US language/non-US geography/non-human study . | Non-relevant outcome |
| 191 | Rubanovich CK, Mohr DC, Schueller SM. Health App Use Among Individuals With Symptoms of Depression and Anxiety: A Survey Study With Thematic Coding. JMIR Mental Health 2017;4:Non-relevant study design2. | Non-relevant outcome |
| 192 | Runyon R, Brooks A, Maizes VH, et al. Objective and subjective validation of a self-care app to improve student athlete well-being. Global Advances in Health and Medicine 2020;9:162. | Non-relevant outcome |
| 193 | Ryan KA, Babu P, Easter R, et al. A smartphone app to monitor mood symptoms in bipolar disorder: Development and usability study. JMIR Mental Health 2020;7(9) (no pagination). | Non-relevant outcome |
| 194 | Sabri B, Vroegindewey A, Hagos M. Development, feasibility, acceptability and preliminary evaluation of the internet and mobile phone-based BSHAPE intervention for Immigrant survivors of cumulative trauma. Contemporary Clinical Trials 2021;110 (no pagination). | Non-relevant outcome |
| 195 | Sasaki N, Obikane E, Vedanthan R, et al. Implementation Outcome Scales for Digital Mental Health (iOSDMH): Scale Development and Cross-sectional Study. JMIR Formative Research 2021;5:Non-relevant study design4332. | Non-US language/non-US geography/non-human study |
| 196 | Schmidt M, Babcock L, Kurowski BG, et al. Usage Patterns of an mHealth Symptom Monitoring App Among Adolescents With Acute Mild Traumatic Brain Injuries. The Journal of head trauma rehabilitation. 2022;01. | Non-relevant outcome |
| 197 | Schmidt M, Glaser N, Riedy T, et al. Learning experience design of an mHealth intervention for parents of children with epilepsy. International Journal of Medical Informatics 2022;160 (no pagination). | Non-relevant outcome |
| 198 | Scullin MK, Jones W, Benge J. Using Smartphone Personal Assistant Technology to Support Prospective Memory in Mild Cognitive Impairment: Preliminary Lessons on Training Feasibility and Phone Usability. Alzheimer's and Dementia 2019;15(7 Supplement):P169. | Non-relevant outcome |
| 199 | Scullin MK, Jones WE, Phenis R, et al. Using smartphone technology to improve prospective memory functioning: a randomized controlled trial. Journal of the American Geriatrics Society 2021. | Non-relevant digital therapeutic |
| 200 | Segal ZV, Dimidjian S, Beck A, et al. Outcomes of Online Mindfulness-Based Cognitive Therapy for Patients with Residual Depressive Symptoms: A Randomized Clinical Trial. JAMA Psychiatry 2020;77(6):563-573. | Non-relevant outcome |
| 201 | Seidman LC, Martin SR, Trant MW, et al. Feasibility and Acceptance Testing of a Mobile Application Providing Psychosocial Support for Parents of Children and Adolescents With Chronic Pain: Results of a Nonrandomized Trial. Journal of pediatric psychology 2019;44(6):645-655. | Non-relevant digital therapeutic |
| 202 | Sikka N, Shu L, Ritchie B, et al. Virtual Reality-Assisted Pain, Anxiety, and Anger Management in the Emergency Department. Telemedicine journal and e-health : the official journal of the American Telemedicine Association 2019;25(12):1207-1215. | Non-relevant outcome |
| 203 | Silk JS, Pramana G, Sequeira SL, et al. Using a Smartphone App and Clinician Portal to Enhance Brief Cognitive Behavioral Therapy for Childhood Anxiety Disorders. Behavior Therapy 2020;51(1):69-84. | Non-relevant outcome |
| 204 | Singh L, Kanstrup M, Depa K, et al. Digitalizing a Brief Intervention to Reduce Intrusive Memories of Psychological Trauma for Health Care Staff Working During COVID-19: Exploratory Pilot Study With Nurses. JMIR Formative Research 2021;5:Non-relevant study design7473. | Non-US language/non-US geography/non-human study |
| 205 | Sinha C, Cheng AL, Kadaba M. Adherence and Engagement With a Cognitive Behavioral Therapy-Based Conversational Agent (Wysa for Chronic Pain) Among Adults With Chronic Pain: Survival Analysis. JMIR Formative Research 2022;6:Non-relevant digital solution 7302. | Non-relevant outcome |
| 206 | Skirrow C, Hobbs M, Barnett JH, et al. Adherence to a Six-Week Study of Wearable Digital Mood and Cognitive Assessments in Depression: Qualitative Insights. Biological Psychiatry 2021;89(9 Supplement):S310. | Non-relevant digital therapeutic |
| 207 | Slade M, Rennick-Egglestone S, Llewellyn-Beardsley J, et al. Recorded Mental Health Recovery Narratives as a Resource for People Affected by Mental Health Problems: Development of the Narrative Experiences Online (NEON) Intervention. JMIR Formative Research 2021;5:Non-relevant study design4417. | Non-relevant outcome |
| 208 | Smith RB, Mahnert ND, Foote J, et al. Mindfulness Effects in Obstetric and Gynecology Patients During the Coronavirus Disease 2019 (COVID-19) Pandemic: A Randomized Controlled Trial. Obstetrics and gynecology 2021;137(6):1032-1040. | Non-relevant outcome |
| 209 | Sockolow P, Schug S, Zhu J, et al. At-risk adolescents as experts in a new requirements elicitation procedure for the development of a smart phone psychoeducational trauma-informed care application. Informatics for health & social care 2017;42(1):77-96. | Non-relevant outcome |
| 210 | Spahrkas SS, Looijmans A, Sanderman R, et al. Beating Cancer-Related Fatigue With the Untire Mobile App: Protocol for a Waiting List Randomized Controlled Trial. JMIR Research Protocols 2020;9:Non-US language/non-US geography/non-human study 5969. | Non-relevant outcome |
| 211 | Stahl S. Digital Monitoring of Sleep, Meals, and Physical Activity as a Preventive Intervention for Depression in Older Bereaved Adults: A Pilot Study on Feasibility, Acceptability, and Symptom Improvement. American Journal of Geriatric Psychiatry 2019;27(3 Supplement):S114. | Non-relevant outcome |
| 212 | Starblanket D, Legare M. Testing Locally Developed Language Apps to Reduce Caregiver Stress and Promote "Aging in Place" as It Relates to Dementia in Indigenous Populations. Alzheimer's and Dementia 2019;15(7 Supplement):P1450. | Non-US language/non-US geography/non-human study |
| 213 | Stiles-Shields C, Montague E, Lattie EG, et al. Exploring User Learnability and Learning Performance in an App for Depression: Usability Study. JMIR Human Factors 2017;4:Non-US language/non-US geography/non-human study 8. | Non-relevant outcome |
| 214 | Strauss C, Dunkeld C, Cavanagh K. Is clinician-supported use of a mindfulness smartphone app a feasible treatment for depression? A mixed-methods feasibility study. Internet Interventions 2021;25:100413. | Non-US language/non-US geography/non-human study |
| 215 | Tarver L, Naslund J, Swaminathan A, et al. 4.62 Evaluating the Acceptability and Outcomes of a Mobile App for Students Seeking Mental Health Care at Harvard University Health Services: A Pilot Study. Journal of the American Academy of Child and Adolescent Psychiatry 2019;58(10 Supplement):S240-S241. | Non-relevant outcome |
| 216 | Taylor CB, Ruzek JI, Fitzsimmons-Craft EE, et al. A systematic digital approach to implementation and dissemination of eating disorders interventions to large populations identified through online screening: implications for post-traumatic stress. Began with 2015 2018;4:25. | Non-relevant digital therapeutic |
| 217 | Topooco N, Fowler LA, Fitzsimmons-Craft EE, et al. Digital interventions to address mental health needs in colleges: Perspectives of student stakeholders. Internet Interventions 2022;28 (no pagination). | Non-relevant outcome |
| 218 | Tripp P, Smirnova M, Niles A, et al. Acceptability and Feasibility of A Digital Health Intervention for Posttraumatic Stress Symptoms: An Entirely Remote Study. Psychosomatic Medicine 2020;82(6):A25. | Non-relevant outcome |
| 219 | Trottier K, Monson CM, Kaysen D, et al. Development of RESTORE: an online intervention to improve mental health symptoms associated with COVID-19-related traumatic and extreme stressors. European journal of psychotraumatology 2021;12(1):1984049. | Non-relevant outcome |
| 220 | Tully L. Innovative approaches to early identification and treatment: using mobile health technology to improve outcomes in psychosis. Schizophrenia bulletin 2018;44:S3‐. | Non-relevant outcome |
| 221 | Vahabzadeh A, Keshav NU, Abdus-Sabur R, et al. Improved Socio-Emotional and Behavioral Functioning in Students with Autism Following School-Based Smartglasses Intervention: Multi-Stage Feasibility and Controlled Efficacy Study. Behavioral sciences 2018;8:20. | Non-relevant outcome |
| 222 | Vailati Riboni F, Sadowski I, Comazzi B, et al. Mindful Age and Technology: a Qualitative Analysis of a Tablet/Smartphone App Intervention Designed for Older Adults. Integrative psychological & behavioral science. 2020;29. | Non-US language/non-US geography/non-human study |
| 223 | Vincent C, Eberts M, Naik T, et al. Provider experiences of virtual reality in clinical treatment. PLoS ONE 2021;16(10 October) (no pagination). | Non-relevant outcome |
| 224 | Viswanathan A, Dodson JA, Blachman N. Gerikit: A geriatric assessment app. Journal of the American Geriatrics Society 2021;69(SUPPL 1):S199. | Non-relevant digital therapeutic |
| 225 | Viswanathan AV, Dodson JA, Blachman NL. GeriKit: a novel app for comprehensive geriatric assessment. Gerontology & geriatrics education 2022:1-8. | Non-relevant digital therapeutic |
| 226 | Wang Y, Liu M, Chi I. An Evaluation of Self-Care and Caregiving Training Curriculum Content for Chinese Immigrant Caregivers: A Participatory Design Approach. Journal of Applied Gerontology 2021;40(12):1818-1827. | Non-relevant digital therapeutic |
| 227 | Wasil AR, Gillespie S, Patel R, et al. Reassessing evidence-based content in popular smartphone apps for depression and anxiety: Developing and applying user-adjusted analyses. Journal of Consulting and Clinical Psychology 2020;88(11):983-993. | Non-relevant outcome |
| 228 | Wasil AR, Palermo EH, Lorenzo-Luaces L, et al. Is There an App for That? A Review of Popular Apps for Depression, Anxiety, and Well-Being. Cognitive and Behavioral Practice. 2021. | Non-relevant audience |
| 229 | Wasil AR, Taylor ME, Franzen RE, et al. Promoting Graduate Student Mental Health During COVID-19: Acceptability, Feasibility, and Perceived Utility of an Online Single-Session Intervention. Frontiers in Psychology 2021;12:569785. | Non-relevant outcome |
| 230 | Wasil AR, Venturo-Conerly KE, Shingleton RM, et al. A review of popular smartphone apps for depression and anxiety: Assessing the inclusion of evidence-based content. Behaviour Research and Therapy 2019;123 (no pagination). | Non-relevant audience |
| 231 | Wickwire EM. The value of digital insomnia therapeutics: What we know and what we need to know commentary on Vedaa et al. Long-term effects of an unguided online cognitive behavioral therapy for chronic insomnia. J Sleep Med. 2019;15(1):101-110. Journal of Clinical Sleep Medicine 2019;15(1):11-13. | Non-US language/non-US geography/non-human study |
| 232 | Wilhelm S, Weingarden H, Greenberg JL, et al. Development and Pilot Testing of a Cognitive-Behavioral Therapy Digital Service for Body Dysmorphic Disorder. Behavior Therapy 2020;51(1):15-26. | Non-relevant outcome |
| 233 | Wilhelm S, Weingarden H, Greenberg JL, et al. Efficacy of App-Based Cognitive Behavioral Therapy for Body Dysmorphic Disorder with Coach Support: Initial Randomized Controlled Clinical Trial. Psychotherapy and Psychosomatics. 2022. | Non-relevant outcome |
| 234 | Wilks CR, Yin Q, Zuromski KL. User Experience Affects Dropout from Internet-Delivered Dialectical Behavior Therapy. Telemedicine journal and e-health : the official journal of the American Telemedicine Association 2020;26(6):794-797. | Non-relevant outcome |
| 235 | Wintner SR, Waters SE, Peechatka A, et al. Evaluation of a scalable online videogame-based biofeedback program to improve emotion regulation: A descriptive study assessing parent perspectives. Internet Interventions 2022;28 (no pagination). | Non-relevant digital therapeutic |
| 236 | Woerner M, Sams N, Rivera Nales C, et al. Generational Perspectives on Technology's Role in Mental Health Care: A Survey of Adults With Lived Mental Health Experience. Frontiers in Digital Health 2022;4:840169. | Non-relevant outcome |
| 237 | Wong NR, Carta KE, Weintraub MJ, et al. Therapeutic alliance in family therapy and clinical outcomes among adolescents at risk for mood disorders: Therapeutic Alliance in Family Therapy. Journal of Affective Disorders 2022;300:66-70. | Non-relevant digital therapeutic |
| 238 | Wright K, Palmer G, Javaid M, et al. Psychological therapy for mood instability within bipolar spectrum disorder: A single-arm feasibility study of a dialectical behaviour therapy-informed approach. Pilot and Feasibility Studies 2020;6(1) (no pagination). | Non-relevant outcome |
| 239 | Yeager CM, Benight CC. Engagement, Predictors, and Outcomes of a Trauma Recovery Digital Mental Health Intervention: Longitudinal Study. JMIR Mental Health 2022;9(5) (no pagination). | Non-relevant audience |
| 240 | Yeager CM, Shoji K, Luszczynska A, et al. Engagement With a Trauma Recovery Internet Intervention Explained With the Health Action Process Approach (HAPA): Longitudinal Study. JMIR Mental Health 2018;5:Non-relevant study design9. | Non-relevant audience |
| 241 | Yeo CJJ, Barbieri A, Roman G, et al. Using smartphone mindfulness apps to increase trainee resilience and reduce burnout. Neurology. Conference: 71st Annual Meeting of the American Academy of Neurology, AAN 2019;92. | Non-relevant outcome |
| 242 | Yu JS, Kuhn E, Miller KE, et al. Smartphone apps for insomnia: Examining existing apps' usability and adherence to evidence-based principles for insomnia management. Translational Behavioral Medicine 2019;9(1):110-119. | Non-relevant digital therapeutic |
| 243 | Zaslavsky O, Chu F, Renn BN. Patient Digital Health Technologies to Support Primary Care Across Clinical Contexts: Survey of Primary Care Providers, Behavioral Health Consultants, and Nurses. JMIR Formative Research 2022;6:Non-relevant digital solution 2664. | Non-relevant audience |
| 244 | Zhang D, Lim J, Zhou L, et al. Breaking the Data Value-Privacy Paradox in Mobile Mental Health Systems through User-Centered Privacy Protection: A Web-Based Survey Study. JMIR Mental Health 2021;8(12) (no pagination). | Non-relevant outcome |
| 245 | Zingg A, Carter L, Rogith D, et al. Mobile Health Applications for Postpartum Depression Management: A Theory-Informed Analysis of Change-Use-Engagement (CUE) Criteria in the Digital Environment. Studies in health technology and informatics 2022;290:844-848. | Non-relevant audience |
| 246 | Zingg A, Rogith D, Refuerzo JS, et al. Digilego for Peripartum Depression: A Novel Patient-Facing Digital Health Instantiation. Amia .. 2020;Annual Symposium proceedings. AMIA Symposium. 2020:1421-1430. | Non-relevant outcome |
| 247 | Zingg A, Singh T, Myneni S. Towards Digestible Digital Health Solutions: Application of a Health Literacy Inclusive Development Framework for Peripartum Depression Management. Amia .. 2021;Annual Symposium proceedings. AMIA Symposium. 2021:1274-1283. | Non-relevant audience |

**Abbreviations:** SLR, systematic literature review; US, United States.

Supplementary Table 10. Publications deprioritized at the full text review stage in the SLR

| **#** | **Reference** | **Reason for deprioritization** |
| --- | --- | --- |
| 1 | Actrn. A randomised controlled trial of a wellbeing app in the general public. https://trialsearch.who.int/Trial2.aspx?TrialID=ACTRN12621001014842 2021. | Protocol |
| 2 | Adams Z, Grant M, Hupp S, et al. Acceptability of an mHealth App for Youth With Substance Use and Mental Health Needs: Iterative, Mixed Methods Design. JMIR Formative Research 2021;5:e30268. | App-specific |
| 3 | Aguilera A, Figueroa CA, Hernandez-Ramos R, et al. MHealth app using machine learning to increase physical activity in diabetes and depression: clinical trial protocol for the DIAMANTE Study. BMJ open 2020;10. | Protocol |
| 4 | Alberts N, Leisenring W, Whitton J, et al. Respiratory monitoring and feedback for chronic pain in adult survivors of childhood cancer: A mobile health pilot study from the childhood cancer survivor study. Pediatric Blood and Cancer. Conference: 52th Congress of the International Society of Paediatric Oncology, SIOP. Virtual. 2020;67. | App-specific |
| 5 | Alon N, Stern AD, Torous J. Assessing the Food and Drug Administration's Risk-Based Framework for Software Precertification With Top Health Apps in the United States: Quality Improvement Study. JMIR mHealth and uHealth 2020;8(10):e20482. | Review of apps against framework |
| 6 | Avalos LA, Aghaee S, Kurtovich E, et al. A mobile health mindfulness intervention for women with moderate to moderately severe postpartum depressive symptoms: Feasibility study. JMIR Mental Health 2020;7(11) (no pagination). | App-specific |
| 7 | Baumel A, Tinkelman A, Mathur N, et al. Digital Peer-Support Platform (7Cups) as an Adjunct Treatment for Women With Postpartum Depression: Feasibility, Acceptability, and Preliminary Efficacy Study. JMIR MHealth and UHealth 2018;6:e38. | App-specific |
| 8 | Beer JM, Smith KN, Kennedy T, et al. A Focus Group Evaluation of Breathe Easier: A Mindfulness-Based mHealth App for Survivors of Lung Cancer and Their Family Members. American journal of health promotion : AJHP 2020;34(7):770-778. | App-specific |
| 9 | Ben-Zeev D, Brian RM, Aschbrenner KA, et al. Video-based mobile health interventions for people with schizophrenia: Bringing the "pocket therapist" to life. Psychiatric rehabilitation journal 2018;41(1):39-45. | App-specific |
| 10 | Ben-Zeev D, Chander A, Tauscher J, et al. A smartphone intervention for people with serious mental illness: Fully remote randomized controlled trial of CORE. Journal of Medical Internet Research 2021;23(11) (no pagination). | App-specific |
| 11 | Bruehlman-Senecal E, Hook CJ, Pfeifer JH, et al. Smartphone app to address loneliness among college students: Pilot randomized controlled trial. JMIR Mental Health 2020;7(10) (no pagination). | App-specific |
| 12 | Buck B, Nguyen J, Porter S, et al. FOCUS mHealth Intervention for Veterans with Serious Mental Illness in an Outpatient Department of Veterans Affairs Setting: Feasibility, Acceptability, and Usability Study. JMIR Mental Health 2022;9(1) (no pagination). | App-specific |
| 13 | Caldeira C, Chan L, Pham V, et al. Mobile apps for mood tracking: an analysis of features and user reviews. Amia .. 2017;Annual Symposium proceedings. AMIA Symposium. 2017:495-504. | Review of apps against framework |
| 14 | Callan JA, Dunbar Jacob J, Siegle GJ, et al. CBT MobileWork<sup>©</sup>: User-Centered Development and Testing of a Mobile Mental Health Application for Depression. Cognitive Therapy and Research 2021;45(2):287-302. | App-specific |
| 15 | Canter K, McIntyre R, Babb R, et al. Results from a community-focused pilot test of the electronic surviving cancer competently intervention program (eSCCIP). Psycho-Oncology 2021;30(SUPPL 1):14-15. | App-specific |
| 16 | Canter KS, McIntyre R, Babb R, et al. A community-based trial of a psychosocial eHealth intervention for parents of children with cancer. Pediatric Blood and Cancer 2022;69(1) (no pagination). | App-specific |
| 17 | Carley I, Vest-Wilcox E, Liebrecht C, et al. Life goals self-management mobile application for bipolar disorder: Consumer acceptability and perceptions. Bipolar Disorders 2021;23(SUPPL 1):97-98. | App-specific |
| 18 | Carpenter-Song E, Jonathan G, Brian R, et al. Perspectives on mobile health versus clinic-based group interventions for people with serious mental illnesses: A qualitative study. Psychiatric Services 2020;71(1):49-56. | App-specific |
| 19 | Carr AL, Jones J, Mikulich Gilbertson S, et al. Impact of a Mobilized Stress Management Program (Pep-Pal) for Caregivers of Oncology Patients: Mixed-Methods Study. JMIR Cancer 2019;5:e11406. | App-specific |
| 20 | Carter L, Rogith D, Franklin A, et al. NewCope: A Theory-Linked Mobile Application for Stress Education and Management. Studies in health technology and informatics 2019;264:1150-1154. | App-specific |
| 21 | Choi SK, Yelton B, Ezeanya VK, et al. Review of the Content and Quality of Mobile Applications About Alzheimer's Disease and Related Dementias. Journal of Applied Gerontology 2020;39(6):601-608. | Review of apps against framework |
| 22 | Chow PI, Drago F, Kennedy EM, et al. A novel mobile phone app intervention with phone coaching to reduce symptoms of depression in survivors of women's cancer: Pre-post pilot study. JMIR Cancer 2020;6(1) (no pagination). | App-specific |
| 23 | Chow PI, Showalter SL, Gerber M, et al. Use of mental health apps by patients with breast cancer in the united states: Pilot pre-post study. JMIR Cancer 2020;6(1) (no pagination). | App-specific |
| 24 | Chow PI, Showalter SL, Gerber MS, et al. Use of Mental Health Apps by Breast Cancer Patients and Their Caregivers in the United States: Protocol for a Pilot Pre-Post Study. JMIR Research Protocols 2019;8:e11452. | Protocol |
| 25 | Cox CE, Hough CL, Jones DM, et al. Effects of mindfulness training programmes delivered by a self-directed mobile app and by telephone compared with an education programme for survivors of critical illness: a pilot randomised clinical trial. Thorax 2019;74:33‐42. | App-specific |
| 26 | Crookston BT, West JH, Hall PC, et al. Mental and Emotional Self-Help Technology Apps: Cross-Sectional Study of Theory, Technology, and Mental Health Behaviors. JMIR Mental Health 2017;4:e45. | App-specific |
| 27 | Dimeff LA, Jobes DA, Koerner K, et al. Using a tablet-based app to deliver evidence-based practices for suicidal patients in the emergency department: Pilot randomized controlled trial. JMIR Mental Health 2021;8. | App-specific |
| 28 | Dubov A, Fraenkel L, Goldstein Z, et al. Development of a smartphone app to predict and improve the rates of suicidal ideation among transgender persons (translife): Qualitative study. Journal of Medical Internet Research 2021;23(3) (no pagination). | App-specific |
| 29 | Fleming JB, Hill YN, Burns MN. Usability of a Culturally Informed mHealth Intervention for Symptoms of Anxiety and Depression: Feedback From Young Sexual Minority Men. JMIR Human Factors 2017;4:e22. | App-specific |
| 30 | Gershkovich M, Middleton R, Hezel DM, et al. Integrating Exposure and Response Prevention With a Mobile App to Treat Obsessive-Compulsive Disorder: Feasibility, Acceptability, and Preliminary Effects. Behavior Therapy 2021;52(2):394-405. | App-specific |
| 31 | Glover AC, Schueller SM, Winiarski DA, et al. Automated Mobile Phone-Based Mental Health Resource for Homeless Youth: Pilot Study Assessing Feasibility and Acceptability. JMIR Mental Health 2019;6:e15144. | App-specific |
| 32 | Gordon JS, Sbarra D, Armin J, et al. Use of a Guided Imagery Mobile App (See Me Serene) to Reduce COVID-19-Related Stress: Pilot Feasibility Study. JMIR Formative Research 2021;5:e32353. | App-specific |
| 33 | Graham AK, Kwasny MJ, Lattie EG, et al. Targeting subjective engagement in experimental therapeutics for digital mental health interventions. Internet Interventions 2021;25 (no pagination). | App-specific |
| 34 | Green J, Neher T, Puzia M, et al. Pregnant women's use of a consumer-based meditation mobile app: A descriptive study. Digital Health 2022;8:20552076221089098. | App-specific |
| 35 | Grubbs KM, Abraham TH, Pyne JM, et al. Enhancing Problem-Solving Therapy With Smartphone Technology: A Pilot Randomized Controlled Trial. Psychiatric services (Washington, D.C.) 2022:appips201900254. | App-specific |
| 36 | Hantsoo L, Criniti S, Khan A, et al. A Mobile Application for Monitoring and Management of Depressed Mood in a Vulnerable Pregnant Population. Psychiatric services (Washington, D.C.) 2018;69:104‐107. | App-specific |
| 37 | Heffner JL, Watson NL, Serfozo E, et al. A Behavioral Activation Mobile Health App for Smokers With Depression: Development and Pilot Evaluation in a Single-Arm Trial. JMIR Formative Research 2019;3:e13728. | App-specific |
| 38 | Heinz AJ, Wiltsey-Stirman S, Jaworski BK, et al. Feasibility and preliminary efficacy of a public mobile app to reduce symptoms of postdisaster distress in adolescent wildfire survivors: Sonoma rises. Psychological services. 2021;15. | App-specific |
| 39 | Holtz BE, McCarroll AM, Mitchell KM. Perceptions and Attitudes Toward a Mobile Phone App for Mental Health for College Students: Qualitative Focus Group Study. JMIR Formative Research 2020;4:e18347. | App-specific |
| 40 | Howarth A, Quesada J, Donnelly T, et al. The development of 'Make One Small Change': an e-health intervention for the workplace developed using the Person-Based Approach. Digital Health 2019;5:2055207619852856. | App-specific |
| 41 | Huang HY, Bashir M. Users' Adoption of Mental Health Apps: Examining the Impact of Information Cues. JMIR MHealth and UHealth 2017;5:e83. | Review of apps against framework |
| 42 | Huberty J, Eckert R, Larkey L, et al. Experiences of Using a Consumer-Based Mobile Meditation App to Improve Fatigue in Myeloproliferative Patients: Qualitative Study. JMIR Cancer 2019;5:e14292. | App-specific |
| 43 | Huberty J, Green J, Glissmann C, et al. Efficacy of the Mindfulness Meditation Mobile App "Calm" to Reduce Stress Among College Students: Randomized Controlled Trial. JMIR MHealth and UHealth 2019;7:e14273. | App-specific |
| 44 | Huberty J, Puzia M, Eckert R, et al. Cancer patients' and survivors' perceptions of the calm app: Cross-sectional descriptive study. JMIR Cancer 2020;6(1) (no pagination). | App-specific |
| 45 | Jallo N, Thacker LR, Menzies V, et al. A Stress Coping App for Hospitalized Pregnant Women at Risk for Preterm Birth. Mcn 2017;The American journal of maternal child nursing. 42(5):257-262. | App-specific |
| 46 | Jonathan GK, Dopke CA, Michaels T, et al. A smartphone-based self-management intervention for bipolar disorder (livewell): User-centered development approach. JMIR Mental Health 2021;8(4) (no pagination). | App-specific |
| 47 | Jonathan GK, Dopke CA, Michaels T, et al. A smartphone-based self-management intervention for individuals with bipolar disorder (livewell): Qualitative study on user experiences of the behavior change process. JMIR Mental Health 2021;8(11) (no pagination). | App-specific |
| 48 | Kaveladze BT, Wasil AR, Bunyi JB, et al. User Experience, Engagement, and Popularity in Mental Health Apps: Secondary Analysis of App Analytics and Expert App Reviews. JMIR Human Factors 2022;9:e30766. | Review of apps against framework |
| 49 | Kennard BD, Goldstein T, Foxwell AA, et al. As Safe as Possible (ASAP): a Brief App-Supported Inpatient Intervention to Prevent Postdischarge Suicidal Behavior in Hospitalized, Suicidal Adolescents. American journal of psychiatry 2018;175:864‐872. | App-specific |
| 50 | Kim B, Lee D, Min A, et al. PuzzleWalk: A theory-driven iterative design inquiry of a mobile game for promoting physical activity in adults with autism spectrum disorder. PLoS ONE 2020;15(9 September 2020) (no pagination). | App-specific |
| 51 | Kizakevich PN, Eckhoff R, Brown J, et al. PHIT for Duty, a Mobile Application for Stress Reduction, Sleep Improvement, and Alcohol Moderation. Military medicine 2018;183(1 Supplement):353-363. | App-specific |
| 52 | Kozlov E, Bantum E, Pagano I, et al. The reach, use, and impact of a free mHealth mindfulness app in the general population: Mobile data analysis. JMIR Mental Health 2020;7(11) (no pagination). | App-specific |
| 53 | Kozlov E, McDarby M, Pagano I, et al. The feasibility, acceptability, and preliminary efficacy of an mHealth mindfulness therapy for caregivers of adults with cognitive impairment. Aging & mental health 2021:1-8. | App-specific |
| 54 | Kreyenbuhl J, Record EJ, Himelhoch S, et al. Development and Feasibility Testing of a Smartphone Intervention to Improve Adherence to Antipsychotic Medications. Clinical Schizophrenia and Related Psychoses 2019;13(1):152-167. | App-specific |
| 55 | Lagan S, D'Mello R, Vaidyam A, et al. Assessing mental health apps marketplaces with objective metrics from 29,190 data points from 278 apps. Acta Psychiatrica Scandinavica 2021;144(2):201-210. | Review of apps against framework |
| 56 | Lagan S, Lamont E, Ramakrishnan A, et al. Digital health developments and drawbacks: a review and analysis of top-returned apps for bipolar disorder. International Journal of Bipolar Disorders 2020;8(1) (no pagination). | Review of apps against framework |
| 57 | Laird B, Puzia M, Larkey L, et al. A Mobile App for Stress Management in Middle-Aged Men and Women (Calm): Feasibility Randomized Controlled Trial. JMIR Formative Research 2022;6:e30294. | App-specific |
| 58 | Latour C, O'Byrne L, McCarthy M, et al. Improving mental health in U.S. Veterans using mHealth tools: A pilot study. Health informatics journal 2020;26(4):3201-3214. | App-specific |
| 59 | Lattie EG, Cohen KA, Hersch E, et al. Uptake and effectiveness of a self-guided mobile app platform for college student mental health. Internet Interventions 2022;27 (no pagination). | App-specific |
| 60 | Lau N, O'Daffer A, Yi-Frazier JP, et al. Popular Evidence-Based Commercial Mental Health Apps: Analysis of Engagement, Functionality, Aesthetics, and Information Quality. JMIR mHealth and uHealth 2021;9(7):e29689. | Review of apps against framework |
| 61 | Lehto RH, Heeter C, Allbritton M, et al. Hospice and Palliative Care Provider Experiences With Meditation Using Mobile Applications. Oncology nursing forum 2018;45(3):380-388. | App-specific |
| 62 | Levin ME, Haeger J, Pierce B, et al. Evaluating an Adjunctive Mobile App to Enhance Psychological Flexibility in Acceptance and Commitment Therapy. Behavior modification 2017;41(6):846-867. | App-specific |
| 63 | Levin ME, Hicks ET, Krafft J. Pilot evaluation of the stop, breathe & think mindfulness app for student clients on a college counseling center waitlist. Journal of American college health : J of ACH 2022;70(1):165-173. | App-specific |
| 64 | Ludwig KA, Browne JW, Nagendra A, et al. Horyzons USA: A moderated online social intervention for first episode psychosis. Early Intervention in Psychiatry 2021;15(2):335-343. | App-specific |
| 65 | Malik T, Ambrose AJ, Sinha C. Evaluating User Feedback for an Artificial Intelligence-Enabled, Cognitive Behavioral Therapy-Based Mental Health App (Wysa): Qualitative Thematic Analysis. JMIR Human Factors 2022;9:e35668. | App-specific |
| 66 | McCall T, Ali MO, Yu F, et al. Development of a Mobile App to Support Self-management of Anxiety and Depression in African American Women: Usability Study. JMIR Formative Research 2021;5:e24393. | App-specific |
| 67 | McCallum M, Ho AS, Mitchell ES, et al. Feasibility, Acceptability, and Preliminary Outcomes of a Cognitive Behavioral Therapy-Based Mobile Mental Well-being Program (Noom Mood): Single-Arm Prospective Cohort Study. JMIR Formative Research 2022;6:e36794. | App-specific |
| 68 | McDonnell KK, Owens OL, Beer JM, et al. Empowering lung cancer survivors and family members to "breathe easier": Adaptation and evaluation of a m-health intervention. Journal of Clinical Oncology. Conference 2019;37. | App-specific |
| 69 | McGinnis E, O'Leary A, Gurchiek R, et al. A Digital Therapeutic Intervention Delivering Biofeedback for Panic Attacks (PanicMechanic): Feasibility and Usability Study. JMIR Formative Research 2022;6:e32982. | App-specific |
| 70 | Meyer D, Jayawardana MW, Muir SD, et al. Promoting Psychological Well-Being at Work by Reducing Stress and Improving Sleep: Mixed-Methods Analysis. Journal of Medical Internet Research 2018;20:e267. | App-specific |
| 71 | Moffitt-Carney KM, Duncan AB. Evaluation of a mindfulness-based mobile application with college students: A pilot study. Journal of American college health : J of ACH 2021;69(2):208-214. | App-specific |
| 72 | Mueller NE, Panch T, Macias C, et al. Using Smartphone Apps to Promote Psychiatric Rehabilitation in a Peer-Led Community Support Program: Pilot Study. JMIR Mental Health 2018;5:e10092. | App-specific |
| 73 | Nct. A Mightier Healthcare System. https://clinicaltrials.gov/show/NCT04732806 2021. | Protocol |
| 74 | Nct. App-Based Mindfulness Meditation for People of Color Who Experience Race-Related Stress. https://clinicaltrials.gov/show/NCT05027113 2021. | Protocol |
| 75 | Nct. CALM Pregnancy: Feasibility of Calm for Pregnant Women. https://clinicaltrials.gov/show/NCT04264910 2020. | Protocol |
| 76 | Nct. Examining the Feasibility of a Mobile Mental Health Application in Psychotherapy. https://clinicaltrials.gov/show/NCT04200170 2019. | App-specific |
| 77 | Nct. Reducing Challenging Behaviors in Children With Autism Through Digital Health. https://clinicaltrials.gov/show/NCT05277194 2022. | App-specific |
| 78 | Nct. Remotely Resolving Psychological Stress (Remote RePS). https://clinicaltrials.gov/show/NCT03196999 2017. | Protocol |
| 79 | Nct. Spinal Cord Injury Mental Health Functional Outcomes Improved by Mindfulness. https://clinicaltrials.gov/show/NCT04972773 2021. | Protocol |
| 80 | Nuske HJ, Goodwin MS, Kushleyeva Y, et al. Evaluating commercially available wireless cardiovascular monitors for measuring and transmitting real-time physiological responses in children with autism. Autism Research 2022;15(1):117-130. | App-specific |
| 81 | O'Loughlin K, Neary M, Adkins EC, et al. Reviewing the data security and privacy policies of mobile apps for depression. Internet Interventions 2019;15:110-115. | Review of apps against framework |
| 82 | Pacella-LaBarbara ML, Suffoletto BP, Kuhn E, et al. A Pilot Randomized Controlled Trial of the PTSD Coach App Following Motor Vehicle Crash-related Injury. Academic Emergency Medicine 2020;27(11):1126-1139. | App-specific |
| 83 | Pensak NA, Joshi T, Simoneau T, et al. Development of a Web-Based Intervention for Addressing Distress in Caregivers of Patients Receiving Stem Cell Transplants: Formative Evaluation With Qualitative Interviews and Focus Groups. JMIR Research Protocols 2017;6:e120. | App-specific |
| 84 | Possemato K, Kuhn E, Johnson EM, et al. Development and refinement of a clinician intervention to facilitate primary care patient use of the PTSD Coach app. Translational Behavioral Medicine 2017;7(1):116-126. | App-specific |
| 85 | Purkayastha S, Addepally SA, Bucher S. Engagement and Usability of a Cognitive Behavioral Therapy Mobile App Compared With Web-Based Cognitive Behavioral Therapy Among College Students: Randomized Heuristic Trial. JMIR Human Factors 2020;7:e14146. | App-specific |
| 86 | Ragavan MI, Ferre V, Bair-Merritt M. Thrive: A Novel Health Education Mobile Application for Mothers Who Have Experienced Intimate Partner Violence. Health promotion practice 2020;21(2):160-164. | App-specific |
| 87 | Ramachandran M, Suharwardy S, Leonard SA, et al. 74: Acceptability of postnatal mood management through a smartphone-based automated conversational agent. American Journal of Obstetrics and Gynecology 2020;222(1 Supplement):S62. | App-specific |
| 88 | Ramos G, Aguilera A, Montoya A, et al. App-Based Mindfulness Meditation for People of Color Who Experience Race-Related Stress: Protocol for a Randomized Controlled Trial. JMIR Research Protocols 2022;11:e35196. | App-specific |
| 89 | Reyes AT, Muthukumar V, Bhatta TR, et al. Promoting Resilience Among College Student Veterans Through an Acceptance-and-Commitment-Therapy App: An Intervention Refinement Study. Community mental health journal 2020;56(7):1206-1214. | App-specific |
| 90 | Reyes AT, Serafica R, Sojobi A. College student veterans' experience with a mindfulness- and acceptance-based mobile app intervention for PTSD: A qualitative study. Archives of psychiatric nursing 2020;34(6):497-506. | App-specific |
| 91 | Ryan KA, Smith SN, Yocum AK, et al. The Life Goals Self-Management Mobile App for Bipolar Disorder: Consumer Feasibility, Usability, and Acceptability Study. JMIR Formative Research 2021;5:e32450. | App-specific |
| 92 | Sahin NT, Keshav NU, Salisbury JP, et al. Second Version of Google Glass as a Wearable Socio-Affective Aid: Positive School Desirability, High Usability, and Theoretical Framework in a Sample of Children with Autism. JMIR Human Factors 2018;5:e1. | App-specific |
| 93 | Salsman JM, McLouth LE, Cohn M, et al. A Web-Based, Positive Emotion Skills Intervention for Enhancing Posttreatment Psychological Well-Being in Young Adult Cancer Survivors (EMPOWER): Protocol for a Single-Arm Feasibility Trial. JMIR Research Protocols 2020;9:e17078. | Protocol |
| 94 | Schueller SM, Glover AC, Rufa AK, et al. A Mobile Phone-Based Intervention to Improve Mental Health Among Homeless Young Adults: Pilot Feasibility Trial. JMIR MHealth and UHealth 2019;7:e12347. | App-specific |
| 95 | Schure MB, Howard M, Bailey SJ, et al. Exploring Perceptions of a Computerized Cognitive Behavior Therapy Program in a U.S. Rural Western State. Rural Mental Health 2018;42:174-183. | App-specific |
| 96 | Smith SK, Somers TJ, Kuhn E, et al. A SMART approach to optimizing delivery of an mHealth intervention among cancer survivors with posttraumatic stress symptoms. Contemporary clinical trials 2021;110:106569. | App-specific |
| 97 | Stoll RD, Pina AA, Gary K, et al. Usability of a Smartphone Application to Support the Prevention and Early Intervention of Anxiety in Youth. Cognitive and Behavioral Practice 2017;24(4):393-404. | App-specific |
| 98 | Su Z, Figueiredo MC, Jo J, et al. Analyzing Description, User Understanding and Expectations of AI in Mobile Health Applications. Amia .. 2020;Annual Symposium proceedings. AMIA Symposium. 2020:1170-1179. | App-specific |
| 99 | Suffoletto B, Goldstein T, Gotkiewicz D, et al. Acceptability, Engagement, and Effects of a Mobile Digital Intervention to Support Mental Health for Young Adults Transitioning to College: Pilot Randomized Controlled Trial. JMIR Formative Research 2021;5:e32271. | App-specific |
| 100 | Szigethy E, Solano F, Wallace M, et al. A study protocol for a non-randomised comparison trial evaluating the feasibility and effectiveness of a mobile cognitive-behavioural programme with integrated coaching for anxious adults in primary care. BMJ Open 2018;8(1) (no pagination). | App-specific |
| 101 | Van Dam L, Rietstra S, Van der Drift E, et al. Can an Emoji a Day Keep the Doctor Away? An Explorative Mixed-Methods Feasibility Study to Develop a Self-Help App for Youth With Mental Health Problems. Frontiers in psychiatry Frontiers Research Foundation 2019;10:593. | App-specific |
| 102 | Van Til K, McInnis MG, Cochran A. A comparative study of engagement in mobile and wearable health monitoring for bipolar disorder. Bipolar disorders 2019. | App-specific |
| 103 | Wambach KA, Davis AM, Nelson EL, et al. momHealth: A Feasibility Study of a Multibehavioral Health Intervention for Pregnant and Parenting Adolescent Mothers. Kansas Journal of Medicine 2021;14:176-181. | App-specific |
| 104 | Wang X, Markert C, Sasangohar F. Investigating Popular Mental Health Mobile Application Downloads and Activity During the COVID-19 Pandemic. Human factors 2021:18720821998110. | Review of apps against framework |
| 105 | Weekly T, Walker N, Beck J, et al. A Review of Apps for Calming, Relaxation, and Mindfulness Interventions for Pediatric Palliative Care Patients. Children 2018;5:26. | Review of apps against framework |
| 106 | Weintraub MJ, Ichinose MC, Zinberg J, et al. App-enhanced transdiagnostic CBT for adolescents with mood or psychotic spectrum disorders. Journal of Affective Disorders 2022;311:319-326. | App-specific |
| 107 | Wisniewski H, Liu G, Henson P, et al. Understanding the quality, effectiveness and attributes of top-rated smartphone health apps. Evidence-based mental health 2019;22(1):4-9. | Review of apps against framework |
| 108 | Wu DTY, Xin C, Bindhu S, et al. Clinician Perspectives and Design Implications in Using Patient-Generated Health Data to Improve Mental Health Practices: Mixed Methods Study. JMIR Formative Research 2020;4:e18123. | Review of apps against framework |
| 109 | Zervogianni V, Fletcher-Watson S, Herrera G, et al. A framework of evidence-based practice for digital support, co-developed with and for the autism community. Autism 2020;24(6):1411-1422. | Review of apps against framework |

**Abbreviations:** SLR, systematic literature review.

Supplementary Table 11. Quality assessment of quantitative components of included studies using the CASP cohort study appraisal checklist

| **Study name** | **Did the study address a clearly focused issue?** | **Was the cohort recruited in an acceptable way?** | **Was the exposure accurately measured to minimize bias?** | **Was the outcome accurately measured to minimize bias?** | **Have the authors identified all important confounding factors?** | **Have the authors taken account of confounding factors in the design and/or analysis?** | **Was follow up of subjects complete enough?** | **Was the follow up of subjects long enough?** | **Are results clearly reported?** | **How precise are the results?** | **Are results believable?** | **Can results be applied to the local population?** | **Do results of this study fit with other available evidence?** | **What are the implications of the study for practice?** |
| --- | --- | --- | --- | --- | --- | --- | --- | --- | --- | --- | --- | --- | --- | --- |
| Afra 2018 | **Y** | **U** | **Y** | **N** | NA | NA | NA | NA | **Y** | NA | **Y** | **Y** | **Y** | **Y** |
| Beard 2019 | **Y** | **Y** | **Y** | **Y** | NA | NA | NA | NA | **Y** | NA | **Y** | **Y** | **Y** | **Y** |
| Borghouts 2022 | **Y** | **Y** | **Y** | **N** | NA | NA | NA | NA | **Y** | NA | **Y** | **Y** | **U** | **Y** |
| Buck 2021a | **Y** | **Y** | **Y** | **Y** | NA | NA | NA | NA | **Y** | NA | **Y** | **Y** | **U** | **Y** |
| Buck 2021b | **Y** | **Y** | **Y** | **Y** | NA | NA | **U** | **U** | **Y** | NA | **Y** | **N** | **Y** | **Y** |
| Forma 2022 | **Y** | **U** | **Y** | **N** | NA | NA | NA | NA | **Y** | **Y** | **Y** | **Y** | **Y** | **Y** |
| Hoffman 2019 | **Y** | **U** | **Y** | **N** | NA | NA | NA | NA | **Y** | NA | **Y** | **Y** | **Y** | **Y** |
| Kern 2018 | **Y** | **Y** | **Y** | **N** | NA | NA | NA | NA | **Y** | NA | **Y** | **Y** | **Y** | **Y** |
| Lipschitz 2019 | **Y** | **Y** | **Y** | **Y** | NA | NA | NA | NA | **Y** | NA | **Y** | **N** | **Y** | **Y** |
| Mata-Greve 2021 | **Y** | **N** | **N** | **Y** | **Y** | **N** | NA | NA | **Y** | NA | **Y** | **N** | **Y** | **Y** |
| Melcher 2020 | **Y** | **Y** | **Y** | **N** | NA | NA | NA | NA | **Y** | NA | **Y** | **N** | **Y** | **Y** |
| Schueller 2018 | **Y** | **N** | **Y** | **N** | NA | NA | NA | NA | **Y** | NA | **Y** | **N** | **U** | **Y** |
| Torous 2018 | **Y** | **Y** | **Y** | **Y** | NA | NA | NA | NA | **Y** | NA | **Y** | **Y** | **Y** | **Y** |
| Zhou 2020 | **Y** | **Y** | **Y** | **N** | NA | NA | NA | NA | **Y** | **Y** | **Y** | **N** | **Y** | **Y** |

**Abbreviations:** CASP, Critical Appraisal Skills Programme; N, no; NA, not applicable; U, unclear; Y, yes.

Supplementary Table 12. Quality assessment of qualitative components of included studies using the CASP qualitative appraisal checklist

| **Study name** | **Was there a clear statement of the aims of the research?** | **Is a qualitative methodology appropriate?** | **Was the research design appropriate to address the aims of the research?** | **Was the recruitment strategy appropriate to the aims of the research?** | **Was the data collected in a way that addressed the research issue?** | **Has the relationship between researcher and participants been adequately considered?** | **Have ethical issues been taken into consideration?** | **Was the data analysis sufficiently rigorous?** | **Is there a clear statement of findings?** | **Is the research valuable?** |
| --- | --- | --- | --- | --- | --- | --- | --- | --- | --- | --- |
| Borghouts 2022 | **Y** | **Y** | **Y** | **Y** | **Y** | **U** | **Y** | **Y** | **Y** | **Y** |
| Boster 2018 | **Y** | **Y** | **Y** | **Y** | **Y** | **U** | **U** | **N** | **Y** | **Y** |
| Carpenter-Song 2018 | **Y** | **Y** | **Y** | **Y** | **Y** | **U** | **Y** | **Y** | **Y** | **N** |
| Casarez 2019 | **Y** | **Y** | **Y** | **Y** | **Y** | **U** | **Y** | **Y** | **Y** | **Y** |
| Connolly 2018 | **Y** | **Y** | **Y** | **Y** | **Y** | **U** | **Y** | **Y** | **Y** | **Y** |
| Cummings 2019 | **Y** | **Y** | **Y** | **Y** | **Y** | **U** | **Y** | **Y** | **Y** | **Y** |
| Dinkel 2021 | **Y** | **Y** | **Y** | **Y** | **Y** | **U** | **Y** | **Y** | **Y** | **Y** |
| Hoffman 2019 | **Y** | **Y** | **U** | **U** | **Y** | **U** | **U** | **U** | **Y** | **Y** |
| Huberty 2022 | **Y** | **Y** | **U** | **Y** | **Y** | **U** | **Y** | **Y** | **Y** | **Y** |
| Knapp 2021 | **Y** | **Y** | **Y** | **Y** | **Y** | **U** | **Y** | **Y** | **Y** | **Y** |
| Kornfield 2022 | **Y** | **Y** | **Y** | **Y** | **Y** | **U** | **U** | **Y** | **Y** | **Y** |
| Mata-Greve 2021 | **Y** | **Y** | **Y** | **N** | **N** | **U** | **Y** | **U** | **N** | **Y** |
| Melcher 2020 | **Y** | **Y** | **Y** | **N** | **N** | **U** | **Y** | **N** | **Y** | **N** |
| Schueller 2018 | **Y** | **Y** | **Y** | **N** | **Y** | **U** | **Y** | **Y** | **Y** | **Y** |
| Schueller 2021 | **Y** | **Y** | **Y** | **Y** | **Y** | **Y** | **Y** | **Y** | **Y** | **Y** |
| Stiles-Sheilds 2017 | **Y** | **Y** | **Y** | **N** | **N** | **U** | **Y** | **N** | **N** | **Y** |
| Storm 2021 | **Y** | **Y** | **U** | **Y** | **Y** | **U** | **Y** | **Y** | **Y** | **Y** |
| Zhou 2020 | **Y** | **Y** | **Y** | **Y** | **Y** | **U** | **Y** | **N** | **Y** | **Y** |

**Abbreviations:** CASP, Critical Appraisal Skills rogramme; N, no; U, unclear; Y, yes.

Supplementary Figure 1. PRISMA flow diagram for publications identified in the SLR


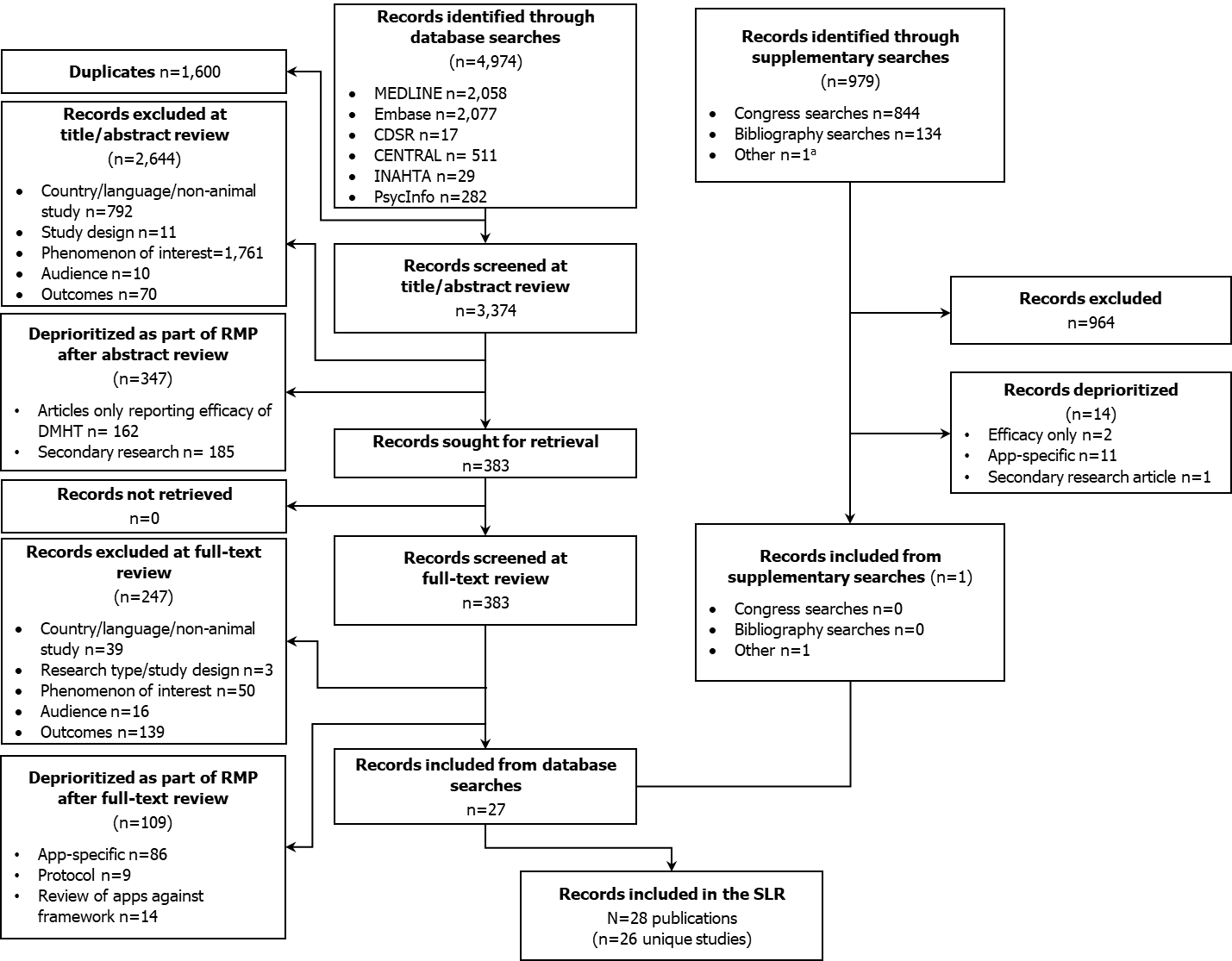


**Footnotes**: ^a^This article was identified as a linked publication to an already included study.
**Abbreviations**: CDSR, Cochrane Database of Systematic Reviews; CENTRAL, Cochrane Central Register of Randomized Controlled Trials; DMHT, digital mental health technologies; INAHTA, International Network of Agencies for Health Technology Assessment; PRISMA, Preferred Reporting Items for Systematic Reviews and Meta-Analyses; RMP, risk management plan; SLR, systematic literature review.
